# Whole-central nervous system functional imaging in larval *Drosophila*

**DOI:** 10.1038/ncomms8924

**Published:** 2015-08-11

**Authors:** William C. Lemon, Stefan R. Pulver, Burkhard Höckendorf, Katie McDole, Kristin Branson, Jeremy Freeman, Philipp J. Keller

**Affiliations:** 1 Howard Hughes Medical Institute, Janelia Research Campus, 19700 Helix Drive, Ashburn, Virginia 20147, USA

## Abstract

Understanding how the brain works in tight concert with the rest of the central nervous system (CNS) hinges upon knowledge of coordinated activity patterns across the whole CNS. We present a method for measuring activity in an entire, non-transparent CNS with high spatiotemporal resolution. We combine a light-sheet microscope capable of simultaneous multi-view imaging at volumetric speeds 25-fold faster than the state-of-the-art, a whole-CNS imaging assay for the isolated *Drosophila* larval CNS and a computational framework for analysing multi-view, whole-CNS calcium imaging data. We image both brain and ventral nerve cord, covering the entire CNS at 2 or 5 Hz with two- or one-photon excitation, respectively. By mapping network activity during fictive behaviours and quantitatively comparing high-resolution whole-CNS activity maps across individuals, we predict functional connections between CNS regions and reveal neurons in the brain that identify type and temporal state of motor programs executed in the ventral nerve cord.

## Abstract (web summary)

To understand how neuronal networks function, it is important to measure neuronal network activity at the systems level. Here Lemon *et al*. develop a framework that combines a high-speed multi-view light-sheet microscope, a whole-CNS imaging assay and computational tools to demonstrate simultaneous functional imaging across the entire isolated *Drosophila* larval CNS.

## Graphical abstract

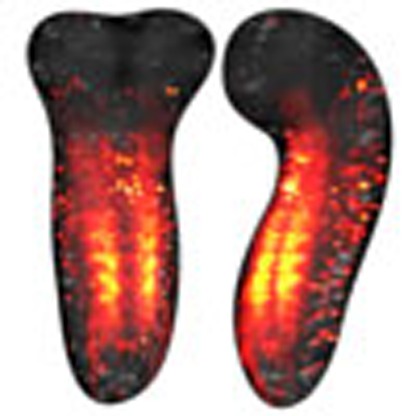

In recent years, advances in neurogenetics and live imaging technology have reshaped the way neurobiologists approach long-standing questions. A new generation of genetically encoded calcium indicators^1^,^2^ in conjunction with sophisticated systems for targeted expression of transgenes allow for non-invasive recording of activity across large populations of neurons^3^,^4^,^5^. In parallel, development of advanced imaging technology such as light-sheet microscopy and light-field microscopy have facilitated high-speed, high-resolution three-dimensional (3D) imaging of large neural tissue volumes^6^,^7^,^8^,^9^,^10^,^11^,^12^,^13^. Together, these technologies bring into reach the possibility of recording from every part of a nervous system during behaviours. Not only would the ability to simultaneously record activity everywhere within an entire central nervous system (CNS) enable measurements of large-scale network dynamics and provide a way to discover and map functional connections between remote CNS regions^14^; whole-CNS functional imaging, that is, simultaneous imaging of both brain and nerve cord, would also afford researchers with opportunities to comprehensively record from motor circuitry while simultaneously imaging activity across the brain. Such a method would thus make it possible to systematically study how brain and nerve cord interact to generate behaviour.

Owing to fundamental technical challenges^15^, however, it has so far not been possible to perform whole-CNS functional imaging in a nervous system larger than that of *Caenorhabditis elegans*^11^. Even in fairly transparent specimens, such as the larval zebrafish, large-scale functional imaging has been limited to the brain^7^,^8^,^16^. There are three major challenges to overcome these limitations: development of imaging technology that tracks activity at physiologically relevant timescales while also maximizing physical coverage and spatial resolution throughout the CNS; development of preparations that allow imaging of neural activity in all parts of the CNS during behaviours; and development of computational methods for interpreting complex patterns of activity at the whole-CNS level and for comparing results across individuals.

The larval fruit fly *Drosophila melanogaster* offers an attractive opportunity for realizing functional imaging of an entire, complex CNS. The larval fruit fly is a genetically tractable model with a sophisticated toolkit for controlling transgene expression^5^,^17^,^18^,^19^ and a CNS that retains the ability to produce locomotor patterns in isolation from sensory input^20^,^21^. However, similar to most model organisms used for functional imaging studies, *Drosophila* larvae also have a fairly opaque nervous system that represents a fundamental problem for comprehensive imaging with light microscopy.

The objective of this paper is to address the three central challenges outlined above and introduce a complete methodological framework—from sample preparation and functional imaging to image analysis—for discovering and analysing functional relationships throughout the entire, non-transparent CNS of larval *Drosophila*. Our methodological framework combines three novel components, each of which is designed to overcome one of the three limitations.

First, we present a light-sheet microscope that enables high-speed multi-view functional imaging with one- or two-photon excitation. Multi-view imaging is essential for optimizing physical coverage of non-transparent specimens^22^,^23^,^24^,^25^, but state-of-the-art technology^22^,^23^,^26^ is by one to two orders of magnitude too slow to resolve the sub-second timescales relevant for functional imaging with calcium indicators. In order to advance imaging performance beyond this critical threshold, we developed a new multi-view imaging technique, termed high-speed simultaneous multi-view (hs-SiMView) microscopy, that improves temporal resolution in *Drosophila*-sized samples by 25-fold.

Second, we integrated these capabilities with a sample preparation strategy and imaging assay for the isolated larval CNS, and demonstrate, for the first time, to our knowledge, functional imaging of neural activity in the entire, non-transparent CNS of a higher invertebrate. We used this assay to image coordinated motor activity patterns throughout the CNS for up to an hour, acquiring 20,000 volumes per sample at a sustained volumetric imaging speed of 5 Hz.

Third, we developed a computational framework for processing and analysing multi-view calcium imaging data produced by hs-SiMView microscopy at a rate of several terabytes per experiment. This framework facilitates mapping functional relationships across the CNS during fictive behaviours and allows quantitative comparison of high-resolution, 3D neural activity maps across multiple nervous systems.

We use our combined system to characterize large-scale patterns of brain activity associated with different locomotor states in the nerve cord, predict functional connections between disparate CNS regions and identify neurons with activity patterns that are representative of the types and temporal states of different motor programs.

## Results

### Light-sheet microscopy for whole-CNS functional imaging

Light-sheet-based functional imaging has proven to be highly effective for observing activity in large populations of neurons. Existing microscopes offer single-view imaging capability suitable for monitoring neural activity in the brain of transparent zebrafish larvae^7^,^8^,^9^ or in surface regions of mammalian neural tissues^6^,^10^,^13^. In a non-transparent, highly light-scattering CNS, such as that of *D. melanogaster*, this approach can only partially penetrate the specimen for fluorescence excitation and detection. Single-view imaging thus gives access to only a small fraction—approximately one quarter—of the *Drosophila* CNS, and coverage of the entire CNS critically requires multi-view imaging capability with at least four complementary views (Supplementary Fig. 1). However, state-of-the-art microscopes with such multi-view imaging capability, such as SiMView light-sheet microscopy, were designed for imaging applications in developmental biology^22^,^23^,^26^, and are by at least one to two orders of magnitude too slow for system-level functional imaging.

**Figure 1:**
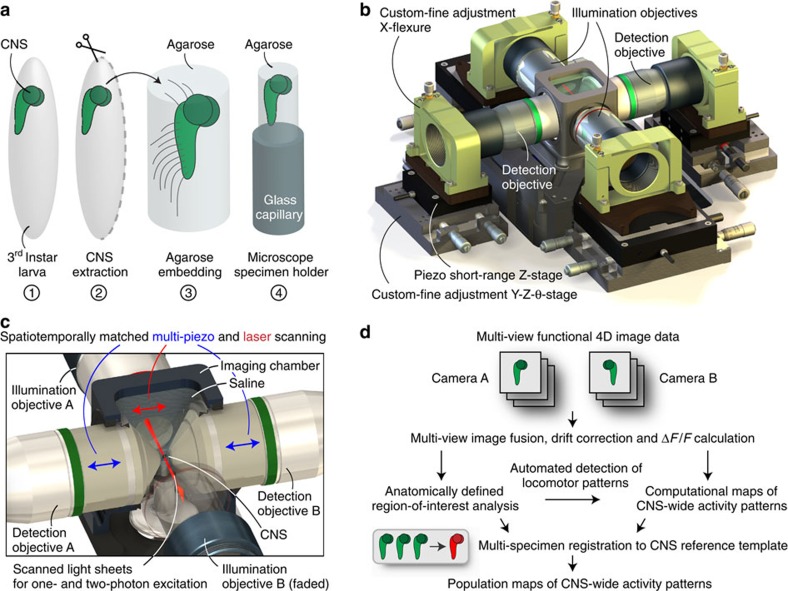
Light-sheet microscopy and computational tools for whole-CNS functional imaging. (**a**) For optimal optical access, the CNS of a *Drosophila* third (3rd) instar larva is extracted by surgery and embedded in a soft, transparent agarose cylinder supported by a glass capillary for mounting in the hs-SiMView light-sheet microscope. (**b**) The CNS explant is then transferred to the microscope's specimen chamber filled with physiological saline. The illustration shows the hs-SiMView microscope core for SiMView functional imaging, including the central specimen chamber, two illumination objectives for bi-directional fluorescence excitation with scanned laser light sheets and two opposing detection objectives mounted on high-speed piezo stages. The 3D volumes covered by the two piezo-operated detection objectives are matched with a precision of a few micrometres using custom Y-Z-θ fine adjustment stages and objective X-flexures. (**c**) In conventional multi-view light-sheet microscopy, the specimen is physically moved through the light sheet for volumetric imaging, which fundamentally constrains imaging speed. In contrast, high-speed multi-view volumetric imaging in hs-SiMView is achieved by keeping the specimen stationary and instead co-translating the focal planes of the two opposing detection systems using piezos. Simultaneously, the thin specimen volume at the location of the focal planes is illuminated bi-directionally with light sheets translated by spatiotemporally matched laser scanning. (**d**) Using a custom processing pipeline for hs-SiMView functional imaging data, the geometry of the specimen is automatically determined from multi-view image data, image foreground is stored using lossless compression in a custom block-based file format, multiple camera views are spatially registered, multi-view image data are fused, specimen drift is compensated locally and globally across the time-lapse experiment, and a Δ*F*/*F* representation of the hs-SiMView time-lapse data set is computed. Using a custom data analysis pipeline, locomotor activity patterns are automatically detected and classified, and high-resolution computational maps of CNS-wide activity timing are constructed for multiple fictive behaviours. In order to quantitatively compare CNS-wide activity maps across multiple nervous systems, a CNS template is constructed from all data sets and subsequently used to transform all image data into a common reference coordinate system using nonlinear spatial registration.

Here we advance SiMView microscopy and present a light-sheet microscope capable of simultaneous multi-view imaging at the speed required for calcium imaging (Fig. 1). This hs-SiMView microscope offers functional imaging with both one- and two-photon excitation, and is the result of four advances spanning microscope design, microscope control and real-time data handling. These concepts are briefly summarized in the following and described in detail in the Methods section (Light-sheet microscope for multi-view functional imaging and Supplementary Fig. 2).

First, we redesigned the SiMView microscope (Fig. 1b, Supplementary Fig. 2 and Supplementary Data 1). Conventional SiMView light-sheet imaging relies on maintaining a stationary optical arrangement and moving the specimen through the light sheets with motorized samples, which fundamentally limits volumetric imaging speed^22^. In hs-SiMView, light sheets and detection focal planes are moved in synchrony relative to the stationary specimen location for 3D optical sectioning, that is, objective positions are continuously adjusted via piezo positioners^7^, light sheets are rapidly translated with galvanometer scanners and the specimen itself is kept stationary (Fig. 1c). This concept is crucial for improving speed fundamentally beyond the limit imposed by the conventional SiMView design but it also introduces several challenges for multi-view imaging. High-speed piezo positioning requires the use of high-stiffness piezos with short travel range on the order of no more than 100–250 μm. Thus, positions and orientations of the 3D fields-of-view associated with each of the four optical arms must be precisely matched to maximize the overall size of the specimen volume accessible by all arms. At the same time, the translational and rotational degrees of freedom required for this purpose must be implemented through a design that minimizes the mechanical footprint and added load on the piezo positioners. These requirements rule out commercially available multi-axis positioning technology, as the relatively large mass of such components would significantly impact piezo performance and thereby slow down the imaging process. We therefore developed custom four-axis stage and flexure systems for the key mechanical positioning tasks in the microscope (Fig. 1b and Supplementary Fig. 2), ensuring robust 3D volume matching with micrometre precision without compromising piezo performance. To enable the reproduction of the imaging technology described here, we provide detailed technical drawings of these critical custom parts of the hs-SiMView microscope (Supplementary Data 1).

Second, we developed a control/acquisition software framework for high-speed piezo-based multi-view imaging. This framework also eliminates previous limitations in raw image acquisition speed^7^,^22^, thereby enabling acquisition of up to 10 terabytes of image data at a sustained rate of 1 GB per second.

Third, we developed a strategy for multi-view microscope alignment that improves spatial overlap of the four focal planes and light sheets throughout the specimen volume. In order to reduce degradation of image quality resulting from spatially varying shifts between focal planes and light sheets (resulting from non-planar optical interfaces along the light path and heterogeneous optical sample properties), hs-SiMView adjusts relative positions of light sheets and detection focal planes, respectively, across the specimen volume on the basis of manual measurements at the onset of the imaging experiment.

Fourth, we developed a multi-view functional imaging assay using two-photon excitation that complements the high-speed imaging mode using one-photon excitation and provides an improved signal-to-background ratio at a reduced imaging speed (180 frames per second with two-photon hs-SiMView versus 370 frames per second with one-photon hs-SiMView).

As a result of the improvements outlined above, hs-SiMView is capable of multi-view functional imaging of a third instar larval CNS-sized volume of 500 × 200 × 200 μm^3^ at 5 Hz with one-photon excitation (Supplementary Movies 1 and 2) and at 2 Hz with two-photon excitation (Supplementary Movies 3,4,5), that is, 25-fold faster than volumetric imaging of similar-sized specimens in previous multi-view light-sheet microscopes^22^,^23^ (see Methods section and Supplementary Methods).

### Estimating spatial resolution across the CNS of larval *Drosophila*

We systematically estimated spatial resolution as a function of location within CNS preparations (see ‘Spatial resolution analysis' in Supplementary Methods section). This analysis takes advantage of fluorescent markers expressed in the nuclei of all cells and provides detailed 3D maps of spatial resolution for various developmental stages (Supplementary Figs 3–6 and Supplementary Movies 6,7,8). These maps show that our light-sheet-based imaging method achieves cellular or even subcellular resolution throughout non-neuropil regions of the isolated first and second instar larval CNS (Supplementary Figs 4 and 5, and Supplementary Movies 6 and 7), and for a large fraction of non-neuropil regions of the isolated third instar larval CNS (Supplementary Fig. 6 and Supplementary Movie 8). We found that, at a regional level, average resolution is highest in the ventral nerve cord (VNC), whereas in the brain (brain lobes and suboesophageal ganglion, SOG) relative resolution is reduced as a result of the longer average illumination and detection path lengths: in first and second instar stages, lateral and axial resolution are on average 22 and 8%, respectively, better in VNC versus brain; and in the third instar stage, lateral and axial resolution are on average 14 and 44%, respectively, better in VNC versus brain. Our estimates of average lateral/axial resolution throughout non-neuropil regions are 1.0/2.5 μm for the first instar stage, 1.1/3.2 μm for the second instar stage and 1.6/5.9 μm for the third instar stage (for detailed statistics, please see ‘Spatial resolution analysis' in Supplementary Methods section).

### Functional imaging of the isolated *Drosophila* CNS

**Figure 2:**
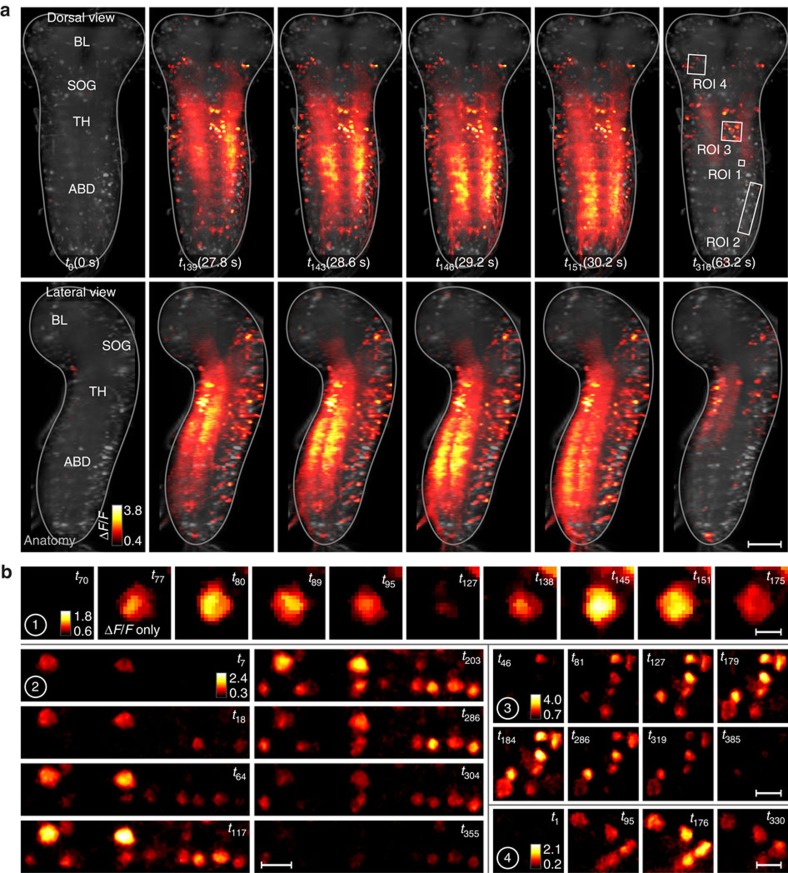
Whole-CNS functional imaging of the isolated *Drosophila* larval nervous system. (**a**) Whole-CNS functional imaging at 5 Hz of a *Drosophila* third instar larval CNS expressing 57C10-GAL4,UAS-GCaMP6s, using hs-SiMView light-sheet microscopy. Imaging was performed with one-photon excitation at 488 nm, maintaining a constant imaging speed of 370 frames per second (491 MB per second) for a period of 1 h. Image panels show maximum-intensity projections of Δ*F*/*F* (colour look-up-table) and CNS anatomy (grey, gamma-corrected GCaMP6s baseline fluorescence) from dorsal (top) and lateral (bottom) views, for six time points during a backward locomotor sequence. Outline indicates CNS boundary. Longer image sequences from this data set are shown in Supplementary Movies 1 and 2. Complementary data sets recorded with two-photon excitation are shown in Supplementary Movies 3,4,5. (**b**) Image sequences showing changes in Δ*F*/*F* for cell bodies in optical sections taken from ROIs in abdomen (ROIs 1 and 2), thorax (ROI 3) and brain (ROI 4) indicated by white rectangles in panel **a**. ROI 1 shows an example of rapid changes in Δ*F*/*F* across two locomotor waves. ROIs 2–4 show examples of slow changes in Δ*F*/*F* across a bout of locomotor waves. Images are median filtered. ABD, abdomen; BL, brain lobes; SOG, suboesophageal ganglion; TH, thorax. Scale bars, 5 μm (**b**, ROI 1), 10 μm (**b**, ROIs 2–4) and 50 μm (**a**).

**Figure 3:**
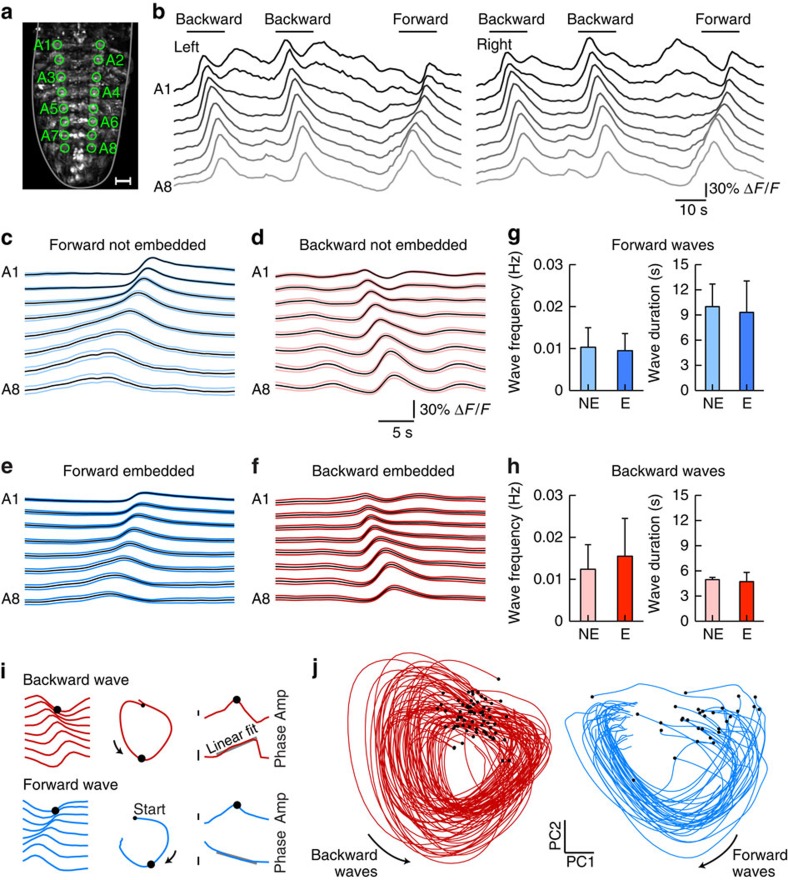
Fictive locomotion in the *Drosophila* larval CNS. (**a**) Neural activity in all VNC hemisegments was captured using 16 3D ROIs. Segments are labelled as A1–A8 from anterior to posterior. (**b**) Activity in hemisegments during example locomotor waves (two backward and one forward), shown separately for the left and right sides of the VNC. Traces show Δ*F*/*F*. Shading indicates segments A1–A8. (**c**,**d**) Average forward (**c**) and backward (**d**) waves in a CNS in physiological saline (black: average, light blue: s.e.m.; *n*=20 waves). The plots show Δ*F*/*F* averaged over left and right sides in a representative preparation. (**e**,**f**) Same as in **c**,**d**, but for an isolated CNS embedded in agarose (*n*=20 waves), which represents the sample preparation used for imaging with hs-SiMView microscopy. (**g**,**h**) Average frequency and duration of forward waves (**g**) and backward waves (**h**) in unembedded (NE; *n*=5 preparations) and embedded preparations (E; *n*=6 preparations). Error bars show s.d. No significant differences in wave frequency or duration were observed between embedded and unembedded preparations (*P*≥0.53 for all comparisons, unpaired *t*-test). (**i**) Illustration of principles underlying our computational module for automated detection and classification of locomotor waves. Left panels: example backward and forward waves; traces show Δ*F*/*F*, with the average response across hemisegments subtracted, to emphasize relative differences. Middle panels: in a 2D space recovered using PCA, locomotor waves are rotations in different directions. Right panels: amplitude (Amp) and phase are computed in the same 2D space; waves are detected as peaks in amplitude and linear ramps in phase. (**j**) Embedding of all detected waves from one specimen in the 2D space recovered by PCA. Each trace is a wave; left, backward; right, forward. Small black dots indicate wave start times. Scale bar, 25 μm (**a**).

We expressed the genetically encoded calcium indicator GCaMP6s panneuronally in third instar larvae^1^ and imaged fluorescence changes associated with neural activity in the isolated CNS using hs-SiMView microscopy with one-photon excitation (Fig. 2 and Supplementary Movies 1 and 2) or two-photon excitation (Supplementary Movies 3,4,5), respectively. Before imaging, CNS explants were embedded in low-melting-point agarose and immersed in physiological saline (Fig. 1a–c). These embedded preparations produced a range of segmentally coordinated activity patterns including waves of anterior progressing and posterior progressing activity in the VNC for up to an hour (Figs 2 and 3, and Supplementary Movies 1,2,3,4,5). These waves represent fictive forward and backward crawling motor patterns, respectively^20^,^21^,^27^.

We compared frequency and duration of forward and backward waves in embedded preparations to those in agarose-free controls (Fig. 3c–h). In embedded preparations, wave durations were 9.3±3.7 and 4.7±1.1 s, and wave frequencies were 9.5±4.1 and 15.5±9.0 mHz for forward and backward waves, respectively (mean±s.d., *n*=6 preparations). In agarose-free preparations, wave durations were 10.0±2.7 and 5.0±0.3 s, and wave frequencies were 10.3±4.6 and 12.4±5.9 mHz for forward and backward waves, respectively (mean±s.d., *n*=5 preparations). Overall, no significant differences in wave frequency or duration were observed between embedded and agarose-free preparations (*P*≥0.53 for all comparisons, unpaired *t*-test), suggesting that sample physiology was well preserved.

Imaging activity in the entire volume of the third instar larval CNS generated large and information-rich data sets. We sampled the volume of the larval CNS using 25–50 million voxels and typically acquired 10 volumes per second (5 volumes per second per camera, 370 images per second), which produced sustained data streams of 500–1,000 MB per second for typically 1 h. For each CNS with an estimated 12,000–15,000 neurons^28^, we thus recorded and analysed several terabytes of multi-view functional image data (see Methods section and Supplementary Methods).

### Computational methods for whole-CNS image analysis

To address the wide spectrum of data processing and data analysis needs arising from our multi-view, whole-CNS functional imaging experiments, we developed a computational framework for hs-SiMView image data (see Fig. 1d, Methods section and Supplementary Data 2). This framework covers all essential image processing and data analysis tasks, including high-throughput lossless compression of the automatically detected image foreground, multi-view registration and fusion of the image foreground, local and global compensation of specimen drift across the time-lapse experiment, computation of a Δ*F*/*F* representation of the functional image data, automated detection and classification of fictive behaviours, and high-resolution mapping of activity timing relationships across the CNS during fictive behaviours. The latter two data analysis methods are explained in more detail in the context of the respective biological analyses presented in the following sections. Further details are provided in the Methods section.

We furthermore implemented a strategy for quantitative comparison of whole-CNS activity timing maps across multiple nervous systems. This method takes advantage of existing nonlinear image registration techniques for neural tissues^29^,^30^ to create a reference template of the *Drosophila* larval nervous system. Using this template, we spatially registered and compared whole-CNS activity maps obtained from different specimens.

For efficient large-scale hs-SiMView data management, our framework automatically determines the geometry of the nervous system from multi-view image data and stores only voxels located in the image foreground containing the specimen, using our custom block-based KLB file format for lossless data compression. These algorithms reduce data size >10-fold.

All of our software modules are highly memory efficient and were designed for processing multi-terabyte data sets in a high-throughput setting. As a result of these design goals, all computations can be rapidly performed on a single computer workstation. The open source code and documentation of all software modules are provided in Supplementary Data 2.

### Mapping whole-CNS activity patterns during fictive behaviours

On first inspection, large numbers of neurons and neuropil regions throughout the CNS showed complex activity patterns during fictive forward and backward crawling. As a first step towards understanding how higher-order control centres (brain lobes and SOG) coordinate the activity of primary larval motor centres (thorax and abdomen), we examined activity timing relationships between presumed higher-order regions and motor centres in *Drosophila* larvae. Specifically, we wanted to determine whether we could identify regions or individual cells in higher-order centres that exhibit distinct and robust activity signatures for specific types of locomotor behaviours or during specific phases of a motor programme.

**Figure 4:**
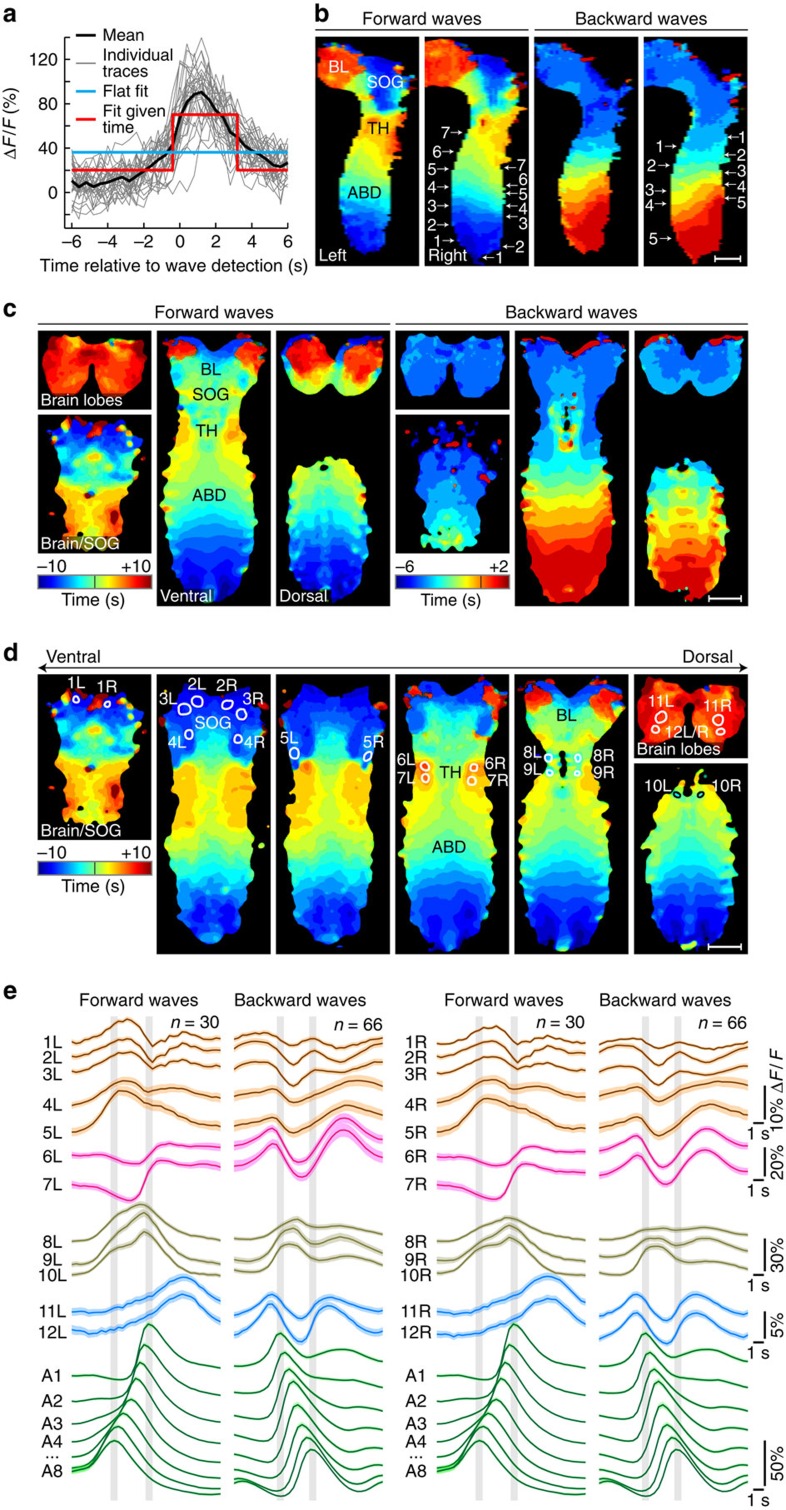
Mapping whole-CNS activity during locomotor behaviours. (**a**) Illustration, for data from one example voxel, of Δ*F*/*F* fits performed for purpose of mapping activity timing during locomotor wave windows. Plots show activity traces in all waves sampled (thin, grey), the mean of these traces (black), the rectangular time-based function fit (red) and the flat, time-independent fit (blue). In this example, the time-based fit is much better than the flat fit. This analysis is performed independently for all voxel locations in the image volume. (**b**,**c**) Whole-CNS activity timing maps for forward and backward waves (**b**, dorsoventral slices; **c**, lateral slices). The statistic shown in **a** was used to capture relative timing of activity across all detected wave events in one specimen (forward: *n*=30, backward: *n*=70). Intuitively, this map shows, for each part for the CNS, the time during locomotor wave windows when activity increases. Arrows in **b** mark relative progression of locomotor waves on dorsal/ventral sides of the VNC (ascending numbers). Forward and backward wave window sizes were defined as [−10 s, 10 s] and [−6 s, 2 s] (centred on waves in VNC) to ensure wave propagation was captured throughout VNC and overlap of events was avoided. (**d**) Lateral slices from 3D whole-CNS activity timing map of fictive forward locomotor waves (*n*=30), with ROIs outlined in white and black (bottom right panel). ROIs include bilaterally symmetrical regions in SOG with increased activity occurring at beginning of forward locomotor waves (1L/R–5L/R), regions in the thorax and at boundary between thorax and abdomen with increased activity occurring during (8L/R–10L/R) or towards the end (6L/R and 7L/R) of forward waves, and regions at dorsal end of brain lobes with increased activity occurring after execution of forward waves (11L/R and 12L/R). (**e**) Δ*F*/*F* traces of ROIs 1L–12L (left two columns) and 1R–12R (right two columns) indicated in panel **a**, during forward (*n*=30) and backward (*n*=66) locomotor waves. Four backward waves were excluded to avoid temporal overlap. For reference, Δ*F*/*F* traces of segments A1–A8 in the VNC are shown below data for ROIs 1L/R–12L/R. Dark lines indicate average activity, light areas indicate s.e.m. L, left; R, right; ABD, abdomen; BL, brain lobes; SOG, suboesophageal ganglion; TH, thorax. Scale bars, 50 μm (**b**–**d**).

The larval CNS produced multiple types of segmentally coordinated motor activity. We decided to focus on forward and backward fictive crawling as they have the clearest relation to larval behaviour. To automatically detect and distinguish forward and backward waves, we used principal component analysis (PCA) for dimensionality reduction of Δ*F*/*F* signals extracted from abdominal neuropil regions (Fig. 3i). Based on features of these trajectories through PCA space, we systematically extracted event times for forward and backward waves in each experiment (see Fig. 3j, Methods section and Supplementary Data 2). By generating manual ground truth annotations for two complete data sets, we determined that our computational module for detecting and classifying motor activity produced, on average, 94.5% correctly classified hits and 5.5% false alarms (*n*=152; see Methods section). We then used these event times as triggers and computed a mapping of activity timing to locations in the entire CNS for all data sets (see Methods section). This analysis uses machine learning to compute, on a voxel-by-voxel basis, the improvement in fit of a time-locked activity model over a flat response model during network events (Fig. 4a and Supplementary Data 2). On an intuitive level, this method provides a measure, per voxel, of when and to what extent activity increases in the locomotor event window. Such voxel-level information represents a useful complement to analyses based on annotated and segmented structures (presented in the next section), especially when manually vetted annotation is difficult owing to the scale or complexity of the data, and when neuronal response properties need to be mapped across diverse neuronal morphologies.

We used this activity timing analysis to first examine how activity propagates through the entire volume of the larval CNS during forward and backward fictive crawling. By plotting only the timing information provided by this analysis, activity progression can be charted and easily followed through the larval CNS through a large dynamic range of local changes in activity (Fig. 4b,c). Previous anatomical work has determined that dorsal regions of the larval VNC contain primarily motor neurons and premotor circuitry, whereas ventral regions of the VNC contain primarily sensory terminals and interneurons involved in sensory processing^31^,^32^. Given this anatomical organization, we predicted that waves of activity would be most prominent and wave propagation would be fastest in the dorsal regions of the larval VNC. Indeed, wave propagation during both forward and backward waves was faster in dorsal regions versus ventral regions (Fig. 4b and Supplementary Movies 9 and 10): during forward waves, propagation of peak activity from segments 8 to 3 was 29% faster dorsally versus ventrally (speed measured from median activity timing of *n*=30 waves); during backward waves, propagation of peak activity from abdominal segments 1 to 6 was 60% faster dorsally versus ventrally (*n*=70 waves). Moreover, during forward waves, peaks in dorsal regions significantly preceded those in ventral regions in all abdominal segments (*P*<10^−5^, sign test, *n*=30 per segment); during backward waves, peaks in dorsal regions significantly preceded those in ventral regions in abdominal segments 8 through 3 (*P*<10^−13^ for all segments, sign test, *n*=70 per segment), whereas activity timing for the first two segments was indistinguishable in dorsal and ventral regions. Increase in activity in dorsal regions was higher than in VNC regions by a factor of 3.9±1.1 during forward waves (mean±s.d., *n*=227) and by a factor of 3.1±1.2 during backward waves (mean±s.d., *n*=495).

Interestingly, as activity propagated forward through the VNC, activity also propagated from the SOG to the dorsal-most regions of the brain (Fig. 4b,c, left panels; Supplementary Movie 9). Statistical comparison of activity peak times confirmed that peaks in SOG regions significantly preceded those in ventro–caudal brain lobe regions (*P*<0.001, sign test, *n*=30), which in turn significantly preceded those in dorsal brain lobe regions (*P*<0.001, sign test, *n*=30).

Our timing analysis also revealed local hot spots with distinct activity patterns, in particular, in the thorax and SOG (Fig. 4c and Supplementary Movies 9 and 10). In order to resolve the fine temporal structure of activity in these regions of interest (ROIs), we extracted fluorescence traces from 24 ROIs within the thorax, SOG and brain lobes that corresponded to boundaries and distinct regions in the activity timing maps (Fig. 4d,e). Peak activity in ROIs corresponded to average timing assignments in the voxel map with a precision exceeding the temporal sampling of the image data (0.15±0.14 s, mean±s.d., *n*=47), confirming that our activity timing maps provided accurate information on relative timing of events in different parts of the larval CNS not only at the voxel level but also at a regional level. Across our set of ROI annotations, we found bilaterally symmetric sites with activity peaks occurring during initiation (before peak in leading abdominal segment), execution (between A1 and A8 peaks) and/or termination (after peak in final abdominal segment) phases of locomotor waves. Activity in SOG ROIs 1–5 (L/R) peaked during the execution phase of forward waves and was maximally suppressed during the execution phase of backward waves. Activity in thoracic ROIs 6–7 (L/R) was bimodal during backward waves, with peaks occurring during initiation and termination phases. Activity in thoracic ROIs 8–10 (L/R) peaked during execution phases of both forward and backward waves. Activity in brain ROIs 11–12 (L/R) was strikingly different during forward and backward waves: activity peaked during the termination phase of forward waves, whereas bimodal activity with peaks during initiation and termination phases was observed for backward waves (Fig. 4e).

### Large-scale profiling of single-neuron activity during behaviours

**Figure 5:**
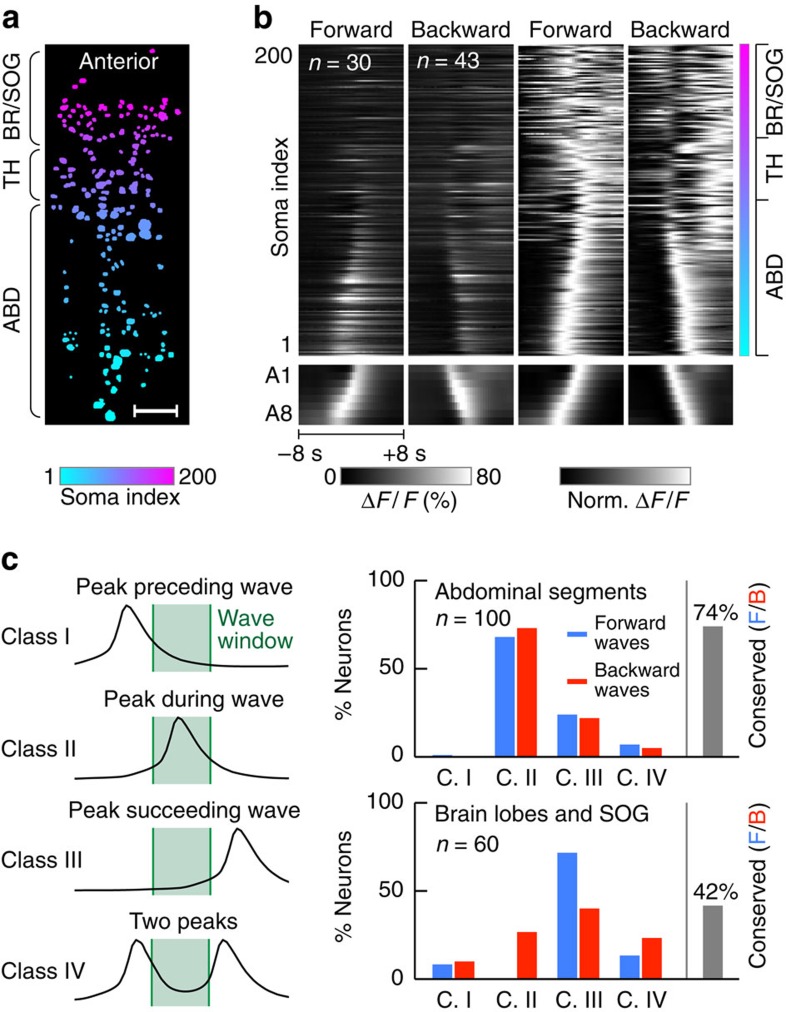
Large-scale profiling of single-neuron activity traces during locomotor behaviours. (**a**) Projection of manually annotated 3D somatic ROIs across the CNS used for extracting signals at the single-neuron level (*n*=200). (**b**) Neuronal activity, averaged across all detected wave time windows. From left to right: raw Δ*F*/*F* for forward waves, raw Δ*F*/*F* for backward waves, normalized (Norm.) Δ*F*/*F* for forward waves, normalized Δ*F*/*F* for backward waves. Greyscale indicates Δ*F*/*F*. Matrices above show somatic responses (colour bar to the right indicates location as shown in panel **a**). Matrices below show corresponding VNC signals (A1–A8, averaged across left and right hemisegments). The global window size was defined as [−8 s, 8 s] (centred on locomotor waves in the VNC) to ensure that wave propagation was captured throughout the VNC, and that comparable statistics were obtained for forward and backward waves without overlap of adjacent event windows. (**c**) Classification of activity traces of all annotated somas. Left: conceptual illustration of the four classes distinguished in this analysis, including peak activity occurring before wave propagation (defined as the time interval marked by peak activity in abdominal segments A1 and A8, class 1), peak activity occurring during wave propagation (class 2), peak activity occurring after wave propagation (class 3) and two activity peaks separated by a phase of quiescence (defined as a ≥50% reduction in Δ*F*/*F* relative to both peaks, class 4). Right: percentages of somas in each class (blue, forward waves; red, backward waves), shown separately for somas in abdominal segments (top) and somas in brain (bottom). Grey bars to the right indicate the percentages of somas with the same classification in forward and backward waves. ABD, abdomen; BR, brain; SOG, suboesophageal ganglion; TH, thorax. Scale bar, 50 μm (**a**).

After mapping information flow through the CNS during forward and backward waves at a coarse level, we examined activity patterns at the level of individual neurons during these two fictive behaviours. We identified and annotated cell body locations in two preparations imaged with one-photon hs-SiMView (Fig. 5) and two-photon hs-SiMView (Supplementary Fig. 7), respectively.

During forward waves (Fig. 5b,c), most annotated cells in the VNC reached peak activity during wave propagation (defined as the time interval marked by peak activity in abdominal segments 1 and 8): 73% of neurons reached peak activity during wave propagation, whereas 22% reached peak activity after the end of wave propagation (*n*=100). A small fraction (5%) of neurons showed two activity peaks separated by a period of quiescence as forward waves propagated through abdominal segments. In the SOG and brain lobes, activity patterns were more diverse, with a large number of cells becoming active at various phases of the motor programme: 10% of neurons reached peak activity before the start of wave propagation, 27% during wave propagation and 40% after the end of wave propagation (*n*=60). Interestingly, a relatively large fraction of annotated neurons in SOG and brain lobes (23%) showed two activity peaks separated by a period of quiescence as forward waves propagated through abdominal segments.

During backward waves (Fig. 5b,c), neurons in the VNC were largely active with locomotor waves in phase relationships similar to those observed for forward waves: 68% of neurons reached peak activity during wave propagation, 24% after the end of wave propagation and 7% showed two activity peaks separated by a period of quiescence during wave propagation (*n*=100). In contrast, in the SOG and brain lobes, activity patterns were quite distinct from those observed during forward waves: the vast majority of cells (72%) were active after the end of the backward wave propagation, and 8% of cells were active before the initiation of the backward wave (*n*=60). Thirteen per cent of neurons showed two activity peaks separated by a period of quiescence as the backward wave propagated through abdominal segments.

A comparison of the classification of peak activity in each neuron during forward and backward waves (Fig. 5c) revealed conserved activity signatures for in total 74% of annotated neurons in abdominal segments (*n*=100) and 42% of annotated neurons in the brain lobes and SOG (*n*=60).

### Neurons in the brain identify type and state of motor programs

**Figure 6:**
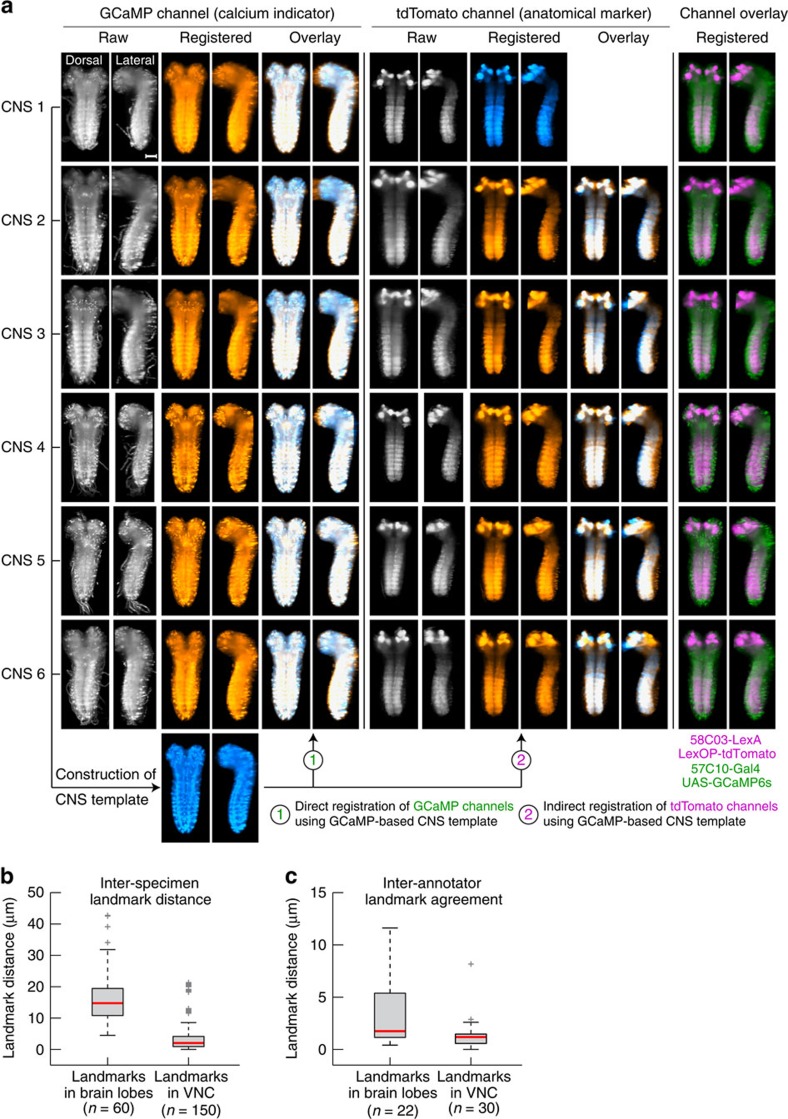
Spatial registration of nervous systems. (**a**) All CNS explants used for functional imaging expressed GCaMP panneuronally (57C10-GAL4,UAS-GCaMP6s) and tdTomato in anatomically defined regions within the larval CNS (58B03-LexA,LexOP-tdTomato; expression in mushroom bodies and neuropil regions). Columns 1–3 show maximum-intensity projections of unregistered GCaMP images (column 1), spatially registered GCaMP images (column 2) and respective overlays (column 3) for six different *Drosophila* third instar larval CNS explants. To register nervous systems, we first constructed a CNS template (shown at the bottom of column 2) from GCaMP reference image stacks representing each of the six independent time-lapse experiments (see Methods section). We then registered image data from each experiment to the CNS reference template using nonlinear spatial registration techniques (see Methods section). Overlays of registered image stacks (orange) and CNS reference template (blue) in column 3 show good anatomical correspondence. Since the CNS reference template used for spatial registration of all nervous systems was constructed exclusively from GCaMP images, we used the tdTomato channel (column 4) to evaluate how well an independent expression pattern can be overlaid across multiple nervous system using our registration workflow. In this control analysis, spatial correspondence depends on quality of the spatial registration and biological variability of the expression pattern. Registration of the tdTomato channels of the six explants to the CNS reference template (column 5) resulted in good overlap of expression patterns in CNS 1–5 (column 6), which appeared to highlight the same biological structures in the CNS. The expression pattern in CNS 6 looked qualitatively different from the others, possibly due to transvection of the LexAop-myr::tdTomato transgene^51^. (**b**) To quantify registration accuracy, two annotators independently determined the 3D positions and relative distances of several CNS landmarks (including landmark locations in abdominal segments and mushroom bodies) in the spatially registered specimens. VNC landmarks were annotated in GCaMP images (obtained by direct registration), whereas brain lobe landmarks were annotated in tdTomato images (indirect registration). The latter measurements thus reflect both accuracy of the registration procedure and biological variability. (**c**) Inter-annotator agreement of landmark positions independently annotated in the same nervous systems. Scale bar, 50 μm (**a**).

Our initial mapping of activity timing throughout the CNS identified small regions within the SOG that showed activity that was strongly synchronized with activity in distant abdominal regions. Closer examination revealed that these regions were often identifiable as cell bodies. However, variations in CNS orientation and shape across preparations made it difficult to assay whether the locations of cell bodies were conserved from animal to animal. To address this problem, we constructed a global CNS anatomy template using GCaMP image data obtained from six nervous systems, following the symmetric group-wise normalization approach (SyGN)^30^, and registered all image data and activity timing maps to this template using B-spline diffeomorphic registration^32^ (see Fig. 6a and Methods section). We used several orthogonal approaches to determine registration accuracy (see Methods section). The average volume overlap between registered, individual nervous systems and global template was 95.2±1.1 % (mean±s.d., *n*=6). The average distance between corresponding, manually annotated landmarks in the registered nervous systems was 4.5±5.9 μm (mean±s.d., *n*=150) for landmarks in the VNC and 16.3±8.6 μm (mean±s.d., *n*=60) for landmarks in the brain (Fig. 6b,c).

**Figure 7:**
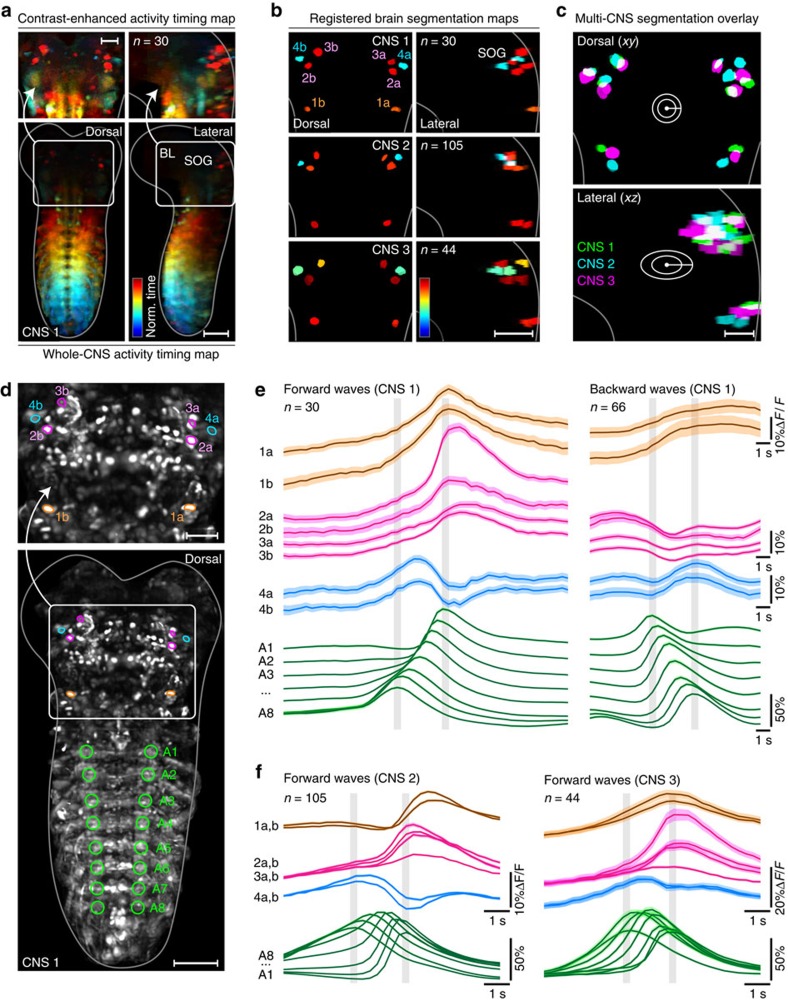
Neurons in the brain identify type and state of motor programs. (**a**) Dorsal (left) and lateral (right) maximum-intensity projections of whole-CNS activity timing map for forward waves (*n*=30). Colour encodes time point of maximum activity. Luminance encodes improvement in activity fit given time (see Methods section). Top panels show enlarged views of brain/SOG, using a luminance look-up-table restricted to dynamic range of data in this region for better visibility. (**b**) Top: maximum-intensity projections of eight manually segmented single-soma ROIs from brain/SOG region highlighted in **a**. Middle and bottom: segmented brain/SOG maps from two additional specimens (CNS 2 and CNS 3), based on 105 and 44 forward waves, respectively. All three nervous systems were spatially co-registered as described in Methods section. Colour code as in **a**. (**c**) Overlay of the three brain/SOG segmentation maps shown in **b**, demonstrating close spatial correspondence of segmented soma locations. Colours indicate specimen identity (green/cyan/magenta: CNS 1/2/3). White circles (*xy*) and ellipses (*xz*) in centre of images indicate average accuracy of the spatial registration procedure (inner circle/ellipse: mean, outer circle/ellipse: mean+1 s.d.). Accuracy in brain is 7.8±4.6 μm (mean±s.d., *n*=60) along *x*- and *y*-directions and 13.0±9.5 μm (mean±s.d., *n*=60) along *z*-direction. (**d**) Dorsal maximum projection of deconvolved image data of CNS 1 (serving as anatomical reference), superimposed with manually segmented single-soma ROIs shown in **b** (top) and hemisegment ROIs for tracking locomotor wave propagation in the VNC (green, segments are labelled as A1–A8). (**e**) Activity traces for eight manually annotated single-soma regions in CNS 1 shown in **b** (top) and **d**, averaged over all forward (left) and backward (right) time windows. Dark lines indicate average activity, light areas indicate s.e.m. (forward: *n*=30, backward: *n*=66). Vertical grey bars indicate time of peak activity in A1 and A8. (**f**) As in **e**, but for forward waves in CNS 2 (*n*=105) and CNS 3 (*n*=44). Data for backward waves are not shown due to low statistics for this fictive behaviour in these two specimens (*n*=6 and 5, respectively). BL, brain lobes; SOG, suboesophageal ganglion. Scale bars, 25 μm (**a**, top; **c** and **d**, top), 50 μm (**a**, bottom; **b** and **d**, bottom).

In three spatially registered nervous systems that each yielded good statistics for locomotor waves (*n*≥30 per specimen), we identified four neurons located ventro-laterally on either side of the SOG with interesting features; across the three preparations, these neurons had highly conserved cell body positions (with an average pairwise distance of 13.8±5.8 μm across preparations, mean±s.d., *n*=24 cell pairs; Fig. 7a–c), strong bilateral symmetry and distinct patterns of activity timing (Fig. 7d–f).

Type 1 neurons were located in the posterior SOG (Fig. 7d). During forward waves, their activity slowly ramped up over a period of several seconds then peaked shortly after the wave had passed the first abdominal segment, marking the end of forward wave propagation (*P*<10^−7^, sign test, *n*=60). During backward waves, activity in these cells slowly ramped up, at a rate similar to their initial behaviour during forward waves, but activity then remained elevated for ∼5 s following the termination of the wave (Fig. 7e).

Activity in type 2 and 3 neurons during forward waves was similar to that observed in type 1 neurons; however, during backward waves, activity slowly decreased over a period of several seconds, then rebounded after the locomotor wave had passed the first three abdominal segments (*P*<10^−11^, sign test, *n*=264; Fig. 7e).

Activity in type 4 neurons during forward waves ramped up and peaked when the wave reached abdominal segment A6 (*P*<10^−5^, sign test, *n*=60), then quickly decreased and rebounded when the wave had passed the first abdominal segment (*P*<10^−12^, sign test, *n*=60). During backward waves, activity in type 4 neurons largely tracked activity observed in abdominal segment A8 (Fig. 7e).

Similar results were obtained in corresponding cell body pairs during forward waves in two other preparations (Fig. 7f). Each cell showed robust, reproducible responses over multiple trials and was information-rich in that the dynamics of activity within each cell reflected both the direction and progression of locomotor waves through the VNC (Fig. 7e,f).

## Discussion

The imaging framework described here represents the first method capable of recording neural activity at near cellular resolution throughout the CNS of a higher invertebrate. By applying this framework to the *Drosophila* larval CNS, we found that representations of fictive locomotor activity are present in presumed higher-order centres at both a regional level and at the level of individual neurons, and that these representations change depending on the mode of fictive locomotion.

At the foundation of our approach is our new hs-SiMView multi-view light-sheet microscope capable of high-speed functional imaging with one- or two-photon excitation. Whole-CNS calcium imaging in a nervous system as large as that of *Drosophila* critically required the design of a microscope with substantially improved temporal resolution, from tens of seconds offered by state-of-the-art multi-view light-sheet microscopy to a few tenths of seconds achieved in hs-SiMView. To enable the reproduction of the imaging technology presented here, we provide detailed technical drawings for all critical custom parts of the hs-SiMView microscope (Supplementary Data 1). From a technical perspective, we found that multi-view imaging at high spatiotemporal resolution is crucial to capture the propagation of activity through the entire *Drosophila* CNS and to systematically identify individual neurons with distinct activity patterns during fictive behaviours. At the same time, the low light exposure and long-term imaging capability provided by our method are essential to collect enough data from a single specimen to build statistically meaningful whole-CNS activity maps. When comparing whole-CNS functional imaging with one-photon and two-photon excitation, we found that the former approach offers superior imaging speed and experiment length (Supplementary Fig. 8), whereas the latter offers superior signal-to-background levels and thereby simplifies large-scale identification of cell bodies (Supplementary Fig. 7).

In order to use this imaging technique to study neural network dynamics at the scale of the entire CNS, we developed a suite of computational tools. In previous work, we and others devised tools for the analysis of large-scale functional imaging data^7^,^8^,^16^,^33^,^34^,^35^. Here, we explored several new directions essential to tackle the challenges encountered when analysing multi-view calcium imaging data in general and motor activity at the scale of an entire CNS in particular. We present approaches for processing and management of multi-view calcium imaging data recorded with hs-SiMView microscopy, automated detection of coordinated motor activity and high-resolution mapping of functional relationships across the CNS during fictive motor behaviours. The methods facilitate efficient processing and analysis of multi-terabyte hs-SiMView image data sets on a single computer workstation. At the same time, many of our tools are also capable of leveraging the strengths of computer clusters, for example, for rapid exploration, screening and comparison of different data processing strategies. Our software modules, including open source code and a software user guide, are provided as Supplementary Data 2.

The overall strength of our combined experimental/computational approach is that it enables the simultaneous, unbiased examination of activity relationships among disparate but functionally connected CNS regions. Thereby, whole-CNS activity patterns are mapped in a single preparation, providing information on multiple fictive behaviours and with cellular resolution for a large fraction of the CNS. In order to identify anatomical regions and individual neurons with conserved activity patterns from these whole-CNS activity maps, we furthermore took advantage of methods for spatial registration of neural tissues^30^,^32^. By combining nonlinear image registration techniques with independent genetic expression systems in *Drosophila*, we implemented and validated a strategy for the quantitative comparison of whole-CNS activity maps across multiple nervous systems. This approach is robust even in the face of relatively large disparities in tissue size, orientation and topology, and may therefore present a useful strategy that could be applied to other animal models as well.

We used our method to chart activity within the entire *Drosophila* larval CNS during multiple fictive behaviours. At the regional scale, as activity progressed from abdominal to lateral thoracic regions during forward waves, activity also progressed from the SOG to the dorsal-most brain regions. These results suggest that at a regional level, spatiotemporal gradients of activity correlated with directional motor patterns may be present in the larval brain. To our knowledge, no previous studies have demonstrated this phenomenon in the CNS of an invertebrate.

Large areas of the larval SOG appear to be active during the initiation phase of forward waves. In contrast, dorsal areas of the brain become active during the termination phase of forward waves. This raises the possibility that the SOG as a region may play a role in the initiation or monitoring of forward locomotion, whereas dorsal brain regions may play a role in its termination. At a coarse spatial level, activity in the SOG did appear to have distinct overall timing relationships relative to other CNS regions. It is important to note, however, that local regions within the SOG showed very strong but heterogeneous activity patterns during motor programs; likewise, thoracic regions also contained several such ‘hot spots'. Based on their distinct timing relationship with the motor programs, some of these regions could be involved in the initiation, termination and/or monitoring of motor activity^36^,^37^. Interestingly, these results also make specific predictions about long-range coarse connectivity between CNS regions. Two testable hypotheses for future study are that strong functional connections exist between the larval SOG and posterior abdominal segments and between dorsal regions of the brain and lateral thoracic regions.

Our analyses were also able to identify single neurons within the SOG exhibiting patterns of activity that, to varying degrees, were representative of the type and temporal state of motor programs. Neurons that encode features of motor behaviours (that is, velocity and acceleration of limbs, as well as activation of muscles) have been identified in motor control centres of the vertebrate brain^38^. Descending neurons in the SOG of the leech, *Hirudo medicinalis*, also exhibit patterns of activity that reflect distinct behavioural states (for example, crawling). When stimulated, these neurons trigger changes in body posture and behavioural state^39^; however, behavioural choice in the leech appears to result from both dynamics of neuronal populations and the actions of a select few neurons that can strongly influence decision making^40^. The variety of candidate neurons, SOG regions and brain lobe areas with network state-related activity profiles discovered in our systematic search may thus provide a foundation for future functional studies aimed at understanding how the larval CNS generates and selects between distinct motor patterns.

In *Drosophila* larvae, a coordinated effort aimed at constructing a complete connectome is currently underway^41^, and the exciting prospect of coupling imaging-based identification of candidate neurons, and whole-CNS imaging in general, to connectomics data is thus on the immediate horizon. Integrating the circuit maps resulting from this work with the functional imaging assay presented here may allow for detailed examination of how behaviour is produced as a function of both neural network architecture and dynamics of activity within interconnected neurons.

Two complementary directions for further research are centred on technology development. On the one hand, further improvement of spatial resolution will generally be desirable, in particular, in applications that require comprehensive single-cell coverage throughout the third instar larval CNS. Possible strategies to this end include the integration of wide-field adaptive optics in the microscope's detection arms^42^, and automated, adaptive rotation and translation of light sheets to compensate for local specimen-induced light refraction in the illumination process^43^. Moreover, dual use of the microscope's four optical arms for light-sheet illumination as well as fluorescence detection may present a very effective approach to improve axial resolution^44^, and could even enable the development of large-scale functional imaging assays with isotropic spatial resolution in the future. On the other hand, to make the most of this new technology, it will be important to also develop strategies for imaging activity in large neural networks simultaneously with optogenetic manipulation. Thereby, the functional role of individual neurons could be investigated in the network context. Moreover, functional imaging assays could be further enriched by taking advantage of advances in voltage indicators^45^, availability of multiple independent genetic expression systems^46^,^47^,^48^,^49^ and the wealth of cell-type specific driver lines that are becoming available for *Drosophila*^18^,^19^.

The method presented here for the isolated larval CNS can be readily adapted to other developmental stages, including *Drosophila* pupae and adults, and may also be suitable for *in vivo* whole-animal functional imaging of *Drosophila* embryos and first instar larvae. We note that this latter approach introduces new challenges in the domain of image processing and data analysis, and will greatly benefit from the development of spatial registration techniques for moving specimens and specimens with dynamically changing topologies. Our hs-SiMView microscope and computational image analysis framework should also be suitable for the study of other biological model organisms. Possible models include not only other invertebrates with a compact CNS but also vertebrates, such as zebrafish embryos and larvae, that benefit greatly from multi-view imaging with light-sheet microscopy^25^, as well as partial CNS explants of animal models with larger nervous systems. The main limitation of our imaging technique concerns sample size, which is constrained by the travel range of the piezo stages and the need for optical access to the sample from multiple directions. The maximum volume accessible with the microscope configuration presented here is 800 × 800 × 250 μm^3^. Larger volumes can be covered by simply switching to different detection objectives and piezo stages with longer travel range; for example, using C-Apochromat × 10/0.45 objectives (Carl Zeiss) in combination with P-629.1CD Hera piezo stages (Physik Instrumente, Karlsruhe) the multi-view imaging volume can be increased to 1,300 × 1,300 × 1,500 μm^3^. The considerably larger field-of-view of this modified microscope design comes at the expense of imaging speed, which is reduced as a result of the lower stiffness of the piezo stages.

It is becoming increasingly clear that to understand how neuronal networks function, it is important to measure neuronal network activity at the system level^14^,^33^. Our method enables, for the first time, the imaging of activity within the entire CNS of a widely used genetically tractable model organism as it generates multiple fictive behaviours. This work not only opens the door to large-scale circuit analyses in *Drosophila* in particular but also lays the foundation for a wider set of future studies by providing an integrated strategy for large-scale functional imaging and analysis of neural activity.

## Methods

### Genetic constructs and transgenic lines

In live imaging experiments for spatial resolution estimation, a genetic construct in which the histone-2Av gene was fused to the gene encoding enhanced green fluorescent protein was used to fluorescently label the nuclei of all larval cells. In all calcium imaging experiments, the GAL4-UAS system^48^ was used to drive expression of the genetically encoded calcium indicator^1^ GCaMP6s. In previous work, a fragment of the promoter for the gene encoding the synaptic protein synaptobrevin was used to create a GAL4 line with panneuronal expression^18^. This GAL4 line (57C10-GAL4) was used to drive expression of GCaMP6s. The LexA-LexOP system^49^ was used to independently drive expression of the red fluorophore tdTomato in anatomically defined regions within the larval CNS. The LexA line (58B03) drove expression in the larval mushroom bodies as well as neuropil regions in the larval CNS.

### Animal care and sample preparation

Fly larvae were raised on standard cornmeal-based food. First, second and third instar animals were selected for use in live imaging experiments for spatial resolution estimation. Feeding third instar animals were used for all calcium imaging experiments. In all experiments, individual larvae were dissected in physiological saline containing (in mM) NaCl (135), KCl (5), CaCl_2_ (2), MgCl2 (4), TES (5) and sucrose (36). After being pinned dorsal side up in Sylgard-lined Petri dishes, a dorsal incision was made along the larval body with fine scissors. The body wall was pinned flat and internal organs were removed. The isolated *Drosophila* CNS was then dissected away and embedded in 1% low-melting temperature agarose in physiological saline at 36 °C. The agarose containing the CNS was drawn into a glass capillary with 1.4 mm inner diameter and 2.0 mm outer diameter, where the agarose quickly cooled to room temperature, forming a soft gel. The agarose cylinder was extruded from the capillary so that the CNS was optically accessible outside of the glass.

### Wide-field fluorescence imaging

Using wide-field fluorescence microscopy, activity patterns in isolated nervous systems embedded in agarose and physiological saline were compared with activity patterns in isolated nervous systems exposed only to physiological saline. Preparations were imaged on an Olympus BX51wi upright compound microscope (Olympus, Center Valley, PA) equipped with an OptoLED light source (Cairn Research, Kent, UK) and an Andor DU897 EMCCD camera (Andor Technologies, Belfast, UK). Images were captured at 10 Hz using Andor IQ software and analysed using custom scripts in Matlab (MathWorks, Natick, MA).

### Light-sheet microscope for multi-view functional imaging

We used the conceptual optical arrangement underlying SiMView light-sheet microscopy^22^ as a starting point for designing our high-speed microscope for multi-view functional imaging with one-photon and two-photon excitation (Supplementary Fig. 2). The realization of whole-CNS multi-view functional imaging experiments at up to 5 Hz (capturing 10 sample volumes per second, with up to 40 images per volume) is the result of four essential advances of the overall design, hardware and software of this earlier microscope, which was capable of multi-view imaging only at up to 0.04 Hz (capturing 0.16 sample volumes per second, with up to 100 images per volume).

First, and in contrast to single-view functional imaging^7^, multi-view volumetric imaging using piezo positioning of the microscope's detection objectives requires a finely adjustable (micrometre precision) and highly robust (multi-day operation of the imaging system) mechanical solution for relative position and orientation alignment of the detection piezos as well as for optimally matching piezo scan ranges to maximize the microscope's shared multi-view volumetric imaging range. We developed a custom two-part mechanical objective positioning system that serves (1) as an adjustable adapter between detection objectives and the piezo stages and (2) as an adjustable base for mounting piezo stages on the optical table (Fig. 1b,c and Supplementary Fig. 2). Technical drawings of these custom parts are provided as Supplementary Data 1. Our custom positioning and rotation adjustment system provides micrometre precision for four key degrees of freedom: *x-y-z*-translation of the detection objective and rotation of the piezo base around the normal vector to the optical table. The custom flexures are manufactured from aluminium and designed to minimize mass and maximize stiffness resulting in minimal added load on the piezo stage (thus optimizing speed). The *y*-translation, *z*-translation and rotation degrees of freedom are incorporated in a highly dimensionally stable stainless steel base and are facilitated by low-friction jewel-bearing guides. Precise movement is achieved through (1) two differentially adjustable sapphire-tipped micrometres opposing a ruby-tipped spring mechanism for *y*-translation and rotation adjustment, and (2) a micrometre-positioned low-friction ceramic wedge for *z*-translation. The *x*-translation degree of freedom is incorporated in the flexure using a precision adjustment screw opposing both gravity and a spring preload, and a highly robust one-piece aluminium frame manufactured by wire electrical discharge machining designed to minimize tilt of the optical axis resulting from the objective's weight distribution. Together, these parts allow fine-tuning of the relative position and orientation of the microscope's four objectives with micrometre precision. The ability to perfectly match the limited scan range (250 μm) of fast piezo stages with high stiffness is furthermore crucial to maximize the sample volume that can be simultaneously imaged in both detection systems. High-speed imaging with these piezo-stage detection arrangements also requires an imaging chamber design that supports fast synchronized movements of multiple detection objectives while maintaining integrity of the water seal at all objective ports. To this end, we designed a specimen chamber with high-precision custom-moulded silicone rubber seals. These silicone seals are sufficiently thin and flexible to allow unimpeded rapid objective movement across the 250-μm piezo travel range while protecting the objective ports from leakage of the physiological saline.

Second, and in contrast to imaging experiments with transparent samples^7^, multi-view functional imaging of a non-transparent sample, with a heterogeneous refractive index distribution that differs significantly from that of the surrounding medium, requires a software framework that accommodates changes in relative position of light sheets and detection objectives as a function of imaging depth into the sample. For this purpose, we introduced a new imaging assay for four-arm functional imaging that allows optimizing microscope alignment on the sample using nonlinear, spatially adaptive, time-dependent operation of all optical arms. This assay enables volumetric multi-view imaging by controlling the piezos that move both detection objectives in synchrony with the galvanometer scanners that move both light sheets. The control software's graphical user interface (GUI) allows the user to define a set of reference planes throughout the sample, which are each associated with a unique set of core alignment parameters (specifically, offsets of the two light sheets and positions of the two detection piezos) such that focal shifts resulting from the sample's refractive index distribution and light-sheet refraction resulting from refractive index mismatches at the buffer-to-agarose and agarose-to-sample interfaces are mitigated through a locally adaptive microscope alignment. The microscope's electronics framework has furthermore been extended to compensate for the differences in piezo response behaviour in a high-speed imaging setting at the limit of the piezos' operating regime. In this high-speed regime, position-versus-voltage vectors vary from one piezo to the next and are furthermore affected by each unit's individual tuning (for achieving maximum speed while minimizing positional fluctuations and time-consuming over-/undershoots at the end points of the volumetric scan range). These differences in response behaviour were measured using an oscilloscope, and compensatory adjustments of each unit's voltage wave forms were introduced through the microscope's control framework.

Third, we improved the electronics framework and developed a new microscope control software and image acquisition workflow for piezo-based multi-view imaging, enabling high-speed data acquisition at sustained rates of 370 images per second and data streams of up to 1,000 MB per second. Image data are furthermore streamed in a header-less binary format to a high-performance RAID system. These improvements, together with the changes outlined above, lead to an increase of volumetric imaging speed from 0.8 to 5 Hz, while additionally providing twice as many view angles of the sample. These advances are crucial to match imaging speed to the timescales of activity in *Drosophila* larval motor circuits and to achieve good physical coverage of the strongly light-scattering *Drosophila* CNS at the same time.

Fourth, we developed a multi-view functional imaging assay using two-photon excitation, which complements the high-speed imaging mode using one-photon excitation. In this two-photon mode, the microscope offers a maximum volumetric imaging speed of 2 Hz for the entire *Drosophila* CNS at high signal-to-noise ratio (Supplementary Movies 3,4,5), although higher speeds are in principle possible (up to 5 Hz) if the respective reduction in signal-to-noise ratio is acceptable. The primary advantages of the two-photon imaging mode are an improvement in signal-to-background ratio and the shift in illumination wavelength from 488 to 940 nm, at which biological tissues generally appear more transparent. In addition, two-photon excitation may be helpful in applications involving concurrent use of genetically encoded calcium indicators and optogenetic effectors.

For detailed information about the components of the hs-SiMView light-sheet microscopy, please see Supplementary Methods.

### High-speed volumetric functional imaging

For volumetric calcium imaging of the *D. melanogaster* larval CNS with light-sheet microscopy, the agarose-embedded larval CNS was transferred to the saline-filled imaging chamber and positioned and oriented using a four-axis mechanical stage that held the specimen stationary in front of the four objectives (Supplementary Fig. 2). The detection arms of the multi-view light-sheet microscope were configured with Nikon × 16/0.8 numerical aperture (NA) water immersion objectives with a working distance of 3 mm. The objectives were positioned with PI Hera piezoelectric nano-positioners with a 250-μm travel range (Physik Instrumente). For one-photon excitation, the specimen was illuminated with two scanned light sheets at 488 nm, focused by Olympus × 4/0.28 NA air illumination objectives through custom-designed curved glass windows on the sides of the specimen chamber. At the waist of the light sheet, its thickness was set to 1.5 μm, and the thickness diverged to 3.0 μm in the most remote locations of the field-of-view corresponding to the lateral edges of the specimen (Supplementary Fig. 3). For two-photon excitation, the specimen was illuminated with scanned light sheets at 940 nm, using Nikon × 10/0.3 NA water-dipping illumination objectives. The light sheets were aligned with the focal planes of the two detection objectives, allowing the two sCMOS cameras to simultaneously obtain images of the illuminated plane of the specimen.

For detailed information about hs-SiMView light-sheet microscope configuration and operation for all high-speed volumetric functional imaging experiments, please see Supplementary Methods.

### Spatial resolution analysis

To quantitatively assess spatial resolution as a function of the spatial location within the specimen, we imaged nervous systems of first, second and third instar animals with a ubiquitous nuclear label (w; His2Av-enhanced green fluorescent protein; +), using the same microscope configuration as for imaging the GCaMP6s expressing nervous systems. By labelling all cell nuclei in the animal, the nuclear marker effectively provided blob-like targets suitable for lateral and axial resolution measurements throughout all non-neuropil regions of the CNS, and thus allowed us to estimate spatial resolution *in situ* under relevant experimental conditions. Volumetric imaging was performed with an axial step size of 2.0 μm. Three-dimensional spatial resolution maps were then computationally reconstructed from the volumetric images using the automated procedure described in the Supplementary Methods.

### Multi-view fusion, processing of calcium imaging data and hs-SiMView data management

We developed a computational pipeline for efficient processing and management of large-scale hs-SiMView image data sets using Matlab (MathWorks, Inc.). All software modules follow a memory-efficient design and take full advantage of multi-core processing architectures, so that typical terabyte-scale hs-SiMView data sets can be rapidly processed on a single computer workstation. By modelling the 3D geometry of the specimen from raw images, image data were first separated into foreground and background voxels. In order to maximize processing performance throughout the computational framework and reduce data size >10-fold, background voxels were discarded and foreground voxels were compressed using our custom block-based lossless compression file format. Multi-view fusion of functional imaging data recorded with the hs-SiMView microscope's two sCMOS cameras was performed by (1) aligning image foreground in multi-view image stacks using a rigid transformation (to correct for physical mismatch of the two cameras^22^), and (2) blending the image content by using the geometrical model of the specimen to determine which camera view provided the, respectively, shortest detection path length for each voxel. This procedure ensured that the final image stacks contained the highest-quality image content from each camera view. The image stacks of each time-lapse experiment were subsequently spatially registered to the image stack acquired at the midpoint of the respective time-lapse recording to correct for specimen drift during the recording. 25-percentile (one-photon imaging) and 10-percentile (two-photon imaging) intensity levels were computed in a 70-time point sliding window throughout each time-lapse data set. The resulting reference stacks were used as a baseline for calculating Δ*F*/*F* for every voxel in the time-lapse data set.

All of our software, including source code for high-speed lossless hs-SiMView data compression, multi-view image registration, multi-view image fusion, spatial registration of time-lapse image data and Δ*F*/*F* calculation are provided as Supplementary Data 2. This archive also contains a user guide with detailed instructions for using the Matlab code.

### Wave detection from ROI time series

To extract basic features of segmentally coordinated activity in isolated preparations, we measured changes in fluorescence within 16 3D ROIs placed within regions that corresponded to abdominal hemisegments (eight left and eight right). Changes in fluorescence (as Δ*F*/*F*) were extracted through spatial averaging within each ROI.

Activity typically alternated between periods of minimal responsiveness, and ‘bouts' including waves of calcium responses either forward (posterior to anterior) or backward (anterior to posterior). These waves could be identified by examining either movies of activity (Fig. 2 and Supplementary Movies 1,2,3,4,5) or segment traces (for example, Fig. 3a,b). We developed an automated procedure to detect the timing and direction of these waves in a standardized fashion across multiple data sets.

First, the following preprocessing steps were applied to the 16 segment time series. The first 120 s of data were ignored to avoid response non-stationarities at the beginning of the recording. Each time series was *z*-scored (subtracting the mean and dividing by the s.d.). The average response across all segments was subtracted from each of the individual time series. This step normalizes for the overall increase in activity across all segments that occurs during waves, and instead highlights relative changes across the different segments. Finally, to focus on symmetric patterns, signals from left and right hemisegments were averaged, yielding eight preprocessed time series.

The detection of waves proceeds in two steps. First, we embed the 8D time series in a 2D space using the singular value decomposition (or PCA). Then, we detect simple features indicative of waves in the resulting 2D space.

More specifically, given a matrix ***X*** that is 8 × *T*, where *T* is the number of time points, we compute the singular value decomposition as:


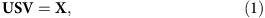


where ***U*** and ***V*** are orthogonal matrices containing the left and right singular vectors and ***S*** is a diagonal matrix with the singular values. The first two columns of ***V***, **v**_1_ and **v**_2_, are *T* × 1 vectors containing a 2D basis for the data. In the resulting 2D space, we found that waves correspond to rotations away from and back toward the origin; forward and backward waves correspond to rotations in different directions (Fig. 3i,j). Thus, it was natural to use amplitude and phase within this 2D space as a means for detecting the presence and direction of waves. Specifically, we computed the amplitude and phase as:


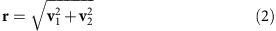



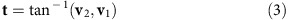


We detected candidate local peaks in the vector **r** (where a local peak is defined as a time point with an amplitude larger than that of its two neighbours, larger than a provided threshold, and separated by at least 3 s). For each candidate peak, we computed the slope of the (unwrapped) phase vector **t** within a window of 2 s. Forward waves were defined as candidate peaks where the slope was positive and greater than a threshold, and backward waves were defined as peaks where the slope was negative and less than a threshold. There are thus only two primary parameters underlying the procedure: a threshold on amplitude, and a threshold on phase slope. Thresholds of 0.04 and 0.125 were found to produce reasonable results across all data sets, and changing these slightly did not qualitatively affect the detection results.

As a control for detection accuracy, in two data sets, wave times were manually annotated by direct inspection of the segment time series. For each annotated wave, counting the presence of an automatically detected wave (or not) was used to define a hit rate. For each automatically detected wave, the absence of an annotated wave (or not) was used to define a false alarm rate. For two data sets, the rates (combined across forward and backward waves) were 93% hits and 3% false alarms, and 96% hits and 8% false alarms, respectively.

The Matlab source code of our software for automatically detecting and classifying forward and backward waves is included in Supplementary Data 2.

### Mapping of whole-CNS activity timing

Several statistical approaches, both exploratory and more targeted, were used to generate computational maps of information flow throughout the CNS, relative to the timing of locomotor waves. In these approaches, statistics are computed on a voxel-by-voxel basis to separately capture the magnitude or informativeness of the response (akin to a measure of signal-to-noise), and the timing of the response in each voxel relative to wave events (detected as described above). Initial surveys of the data were based on simple statistics, such as the s.d. of the response averaged across waves, and the time point of the peak response.

For a more refined analysis, we developed a statistic to measure when and to what extent activity peaks are synchronized with locomotor waves. For each voxel, we create a data set of *N* time series each of length *T*, triggered around the detection of forward or backward locomotor waves. We then measure how well these activity traces are fit using information about the time relative to the wave detection, in comparison to without this information about time. More specifically, we compare how well the activity traces are fit by a rectangular function of fixed radius *r*:


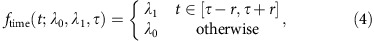


*λ*_0_≤*λ*_1_, to how well they are fit by a flat function:


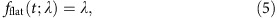


where *t* signifies time. We find the optimal parameters for these regression fits according to mean-squared error. Supplementary Fig. 4a shows an example of activity traces for a given voxel and the corresponding fits *f*_time_ and *f*_flat_.

The optimal fits and their errors can be computed efficiently based on the means and variances of the traces over all waves, and using convolutions. As sample means and variances can be computed efficiently with online updates, this allows these fits to be computed online with minimal memory required.

For the time-based fit, the optimal rectangle location *τ* is the peak of the convolution of the mean activity trace with a box filter of length 2*r*+1:


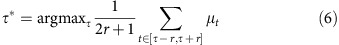


where *μ*_*t*_ is the mean activity over all *N* waves at time *t*. The error of the time-based fit can be computed efficiently as:


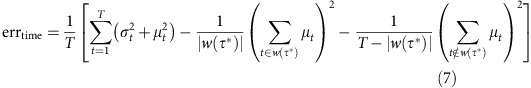


where 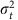 is the sample variance of the traces at time *t* and *w*(τ*)=[τ*−*r*, τ^* +^*r*] is the time interval.

For the time-independent, flat fit, the error is simply the variance:


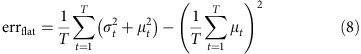


The Matlab source code of our software for mapping whole-CNS activity timing is included in Supplementary Data 2.

### Spatial registration of nervous systems

The *Drosophila* larval CNS template was constructed following the SyGN^30^ using the open source framework Advanced Normalization Tools (https://github.com/stnava/ANTs). Multiple consecutive time points of GCaMP recordings from six independent CNS preparations were averaged to obtain reference volumes for each data set. The segmental nerves were manually masked to focus the image registration on VNC and brain. During SyGN, the images are first aligned by an affine transform to obtain an initial template.

Both template and individual registrations are then iteratively optimized by a deformable registration, such that the deformation of each individual CNS is minimized^30^. We performed SyGN using B-spline diffeomorphic image registration^32^ with a gradient step of 0.1 and five subsampling factors (in pixels: 10, 6, 4, 2, full sampling) and cross-correlation with a neighbourhood radius of 6 as similarity metric.

Registration quality was assessed using the distance of manually annotated, 3D landmarks in VNC and brain, measured with ImageJ^50^ by two annotators. Landmarks in abdominal segments were annotated using the averaged GCaMP reference volumes. Anatomically corresponding landmarks in the brain lobes, specifically equivalent locations within the mushroom bodies, were annotated using 58B03-LexA,LexOP-tdTomato expression patterns recorded for all specimens (Fig. 6a). The average distance of landmarks in the brain lobes resulted as 16.3±8.6 μm (s.d., *n*=60) and the average distance of landmarks in the VNC resulted as 4.5±5.9 μm (s.d., *n*=150). We also determined the inter-annotator agreement for the same landmarks and measured average distances of 3.2±3.0 μm (s.d., *n*=22) in the brain lobes and 1.4±1.5 μm (s.d., *n*=30) in the VNC for the positions labelled by annotators 1 and 2.

Complementing this distance analysis, we also determined the average overlap of the volumes occupied by each individual CNS in the registered GCaMP reference stacks and the volume occupied by the CNS template, respectively. Volume overlap was measured as the fraction of common foregound voxels in the image stacks after applying an adaptive threshold for foreground segmentation (using a threshold of 0.04 times the mean stack intensity for all data sets). The average volume overlap for the six registered CNS preparations resulted as 95.2±1.1% (mean±s.d., *n*=6).

## Additional information

**How to cite this article:** Lemon, W. C. *et al*. Whole-central nervous system functional imaging in larval *Drosophila*. *Nat. Commun*. 6:7924 doi: 10.1038/ncomms8924 (2015).

## Supplementary Material

Supplementary Information Supplementary Figures 1-8 and Supplementary Methods and Supplementary References

Supplementary Data 1 Technical drawings of individual components and complete assemblies of custom four-axis stage and flexure systems in the hs-SiMView light-sheet microscope

Supplementary Data 2 Source code for processing multi-view functional imaging data, detecting and classifying fictive locomotor behaviors, and mapping activity timing across the central nervous system

Supplementary Movie 1 Whole-CNS functional imaging with one-photon multi-view lightsheet microscopy (ΔF/F overlay)
Whole-CNS functional imaging at 5 Hz of a Drosophila 3rd instar larva expressing 57C10- GAL4,UAS-GCaMP6s, using high-speed simultaneous multi-view light-sheet microscopy. Panels show maximum-intensity projections of ΔF/F (color look-up-table) and CNS anatomy (grey) from dorsal (left), lateral (bottom right) and frontal views (top right). The CNS anatomy shown in grey represents GCaMP6s baseline fluorescence, which was gamma-corrected to reduce image contrast and instead highlight overall CNS morphology. Imaging was performed with one-photon excitation at 488 nm, maintaining a constant imaging speed of 370 frames per second (491 MB/s) over a period of one hour. The Movie shows several representative motor sequences (forward and backward waves) recorded during this one-hour observation period. Time is shown relative to the onset of imaging. The average GCaMP baseline intensity level in this recording is shown as a function of time in Supplementary Figure 8 (blue).

Supplementary Movie 2 Whole-CNS functional imaging with one-photon multi-view lightsheet microscopy (raw data and ΔF/F)
Maximum-intensity projections of raw image data (left) and ΔF/F (right) for the one-photon Drosophila whole-CNS functional imaging experiment shown in Supplementary Movie 1. Image data shown to the left was background-corrected using a long-range Gauss filter (100-pixel radius) to reduce the degradation of image contrast resulting from data projection. Time is shown relative to the onset of imaging.

Supplementary Movie 3 Whole-CNS functional imaging with two-photon multi-view lightsheet microscopy (ΔF/F overlay)
Whole-CNS functional imaging at 2 Hz of a Drosophila 3rd instar larva expressing 57C10- GAL4,UAS-GCaMP6s, using high-speed simultaneous multi-view light-sheet microscopy. Panels show maximum-intensity projections of ΔF/F (color look-up-table) and CNS anatomy (grey) from dorsal (left), lateral (bottom right) and frontal views (top right). The CNS anatomy shown in grey represents GCaMP6s baseline fluorescence, which was gamma-corrected to reduce image contrast and instead highlight overall CNS morphology. Imaging was performed with two-photon excitation at 940 nm, maintaining a constant imaging speed of 180 frames per second (354 MB/s) over a period of one hour. The Movie shows several representative motor sequences (forward and backward waves) recorded during this one-hour observation period. Time is shown relative to the onset of imaging. The average GCaMP baseline intensity level in this recording is shown as a function of time in Supplementary Figure 8 (red).

Supplementary Movie 4 Whole-CNS functional imaging with two-photon multi-view lightsheet microscopy (raw data and ΔF/F)
Maximum-intensity projections of raw image data (left) and ΔF/F (right) for the two-photon Drosophila whole-CNS functional imaging experiment shown in Supplementary Movie 3. Image data shown to the left was background-corrected using a long-range Gauss filter (100-pixel radius) to reduce the degradation of image contrast resulting from data projection. Time is shown relative to the onset of imaging.

Supplementary Movie 5 Whole-CNS functional imaging with two-photon multi-view lightsheet microscopy (volume rendering)
Three-dimensional, rotating volume rendering of two backward wave sequences from the twophoton Drosophila whole-CNS functional imaging experiment shown in Supplementary Movie 3. The CNS anatomy shown in grey represents GCaMP6s baseline fluorescence, which was gamma-corrected to artificially reduce image contrast and highlight overall CNS morphology. ΔF/F data is superimposed on CNS anatomy and shown in a color look-up-table. Volume rotation speeds were slightly reduced for the two backward wave sequences in order to better highlight spatiotemporal ΔF/F dynamics during these time intervals. Time is shown relative to the onset of imaging. Cell size in the background grid is 50 μm.

Supplementary Movie 6 Spatial resolution maps for the Drosophila 1st instar larval CNS
Left: Slice-by-slice image sequence of a three-dimensional image stack of a Drosophila 1st instar larval CNS expressing the ubiquitous nuclear label His2Av-eGFP, recorded with our light-sheet microscope for multi-view functional imaging. Middle/right: Slice-by-slice image sequence of the corresponding three-dimensional spatial resolution maps estimating lateral (middle) and axial (right) resolution as a function of spatial location in non-neuropil regions of the 1st instar larval CNS. Resolution was estimated locally for each voxel by analyzing the intensity profile measured for the respective closest cell nucleus, following the procedure described in Section "Spatial Resolution Analysis" in the Supplementary Methods. The position label indicates distance from the ventral surface. The maps were constructed from n = 2,315 (lateral resolution map) and 2,291 (axial resolution map) nuclei measurements, respectively, across non-neuropil regions of the 1st instar larval CNS.

Supplementary Movie 7 Spatial resolution maps for the Drosophila 2nd instar larval CNS
Slice-by-slice image sequence of a three-dimensional image stack of a Drosophila 2nd instar larval CNS expressing the ubiquitous nuclear label His2Av-eGFP (left), as well as corresponding three-dimensional spatial resolution maps estimating lateral (middle) and axial (right) resolution as a function of spatial location in non-neuropil regions of the 2nd instar larval CNS. Please see the caption of Supplementary Movie 6 and Supplementary Methods for more details. The maps were constructed from n = 3,328 (lateral resolution map) and 3,388 (axial resolution map) nuclei measurements, respectively, across non-neuropil regions of the 2nd instar larval CNS.

Supplementary Movie 8 Spatial resolution maps for the Drosophila 3rd instar larval CNS
Slice-by-slice image sequence of a three-dimensional image stack of a Drosophila 3rd instar larval CNS expressing the ubiquitous nuclear label His2Av-eGFP (left), as well as corresponding three-dimensional spatial resolution maps estimating lateral (middle) and axial (right) resolution as a function of spatial location in non-neuropil regions of the 3rd instar larval CNS. Please see the caption of Supplementary Movie 6 and Supplementary Methods for more details. The maps were constructed from n = 12,884 (lateral resolution map) and 14,938 (axial resolution map) nuclei measurements, respectively, across non-neuropil regions of the 3rd instar larval CNS.

Supplementary Movie 9 Whole-CNS activity timing map for forward locomotor waves
Slice-by-slice image sequence of the three-dimensional whole-CNS activity timing map computed for fictive forward locomotor waves (n = 30). For clarity, timing data and the improvement in activity fit given time (referred to as “information gain” or “info. gain” for simplicity) are shown as separate panels. The timing data panel (left, color look-up-table) shows the time point of maximum activity for each voxel, measured relative to the time frame of the forward locomotor wave. The information gain panel (right, greyscale look-up-table) shows the corresponding improvement in root-mean-squared error of the activity fit given time over a flat response. Intuitively, this analysis provides a measure, per voxel, of when (timing) and to what extent (information gain) activity increases at a particular time during the locomotor waves. The spatial resolution of this timing map is limited by the resolution of the underlying image data (Supplementary Fig. 6), i.e. color and information gain assignments for each voxel are representative of the average activity in the respective local spatial neighborhood contributing to this voxel. This movie shows the complete three-dimensional data set underlying the subset of panels shown in Figs. 3b-d and 7a. Note that in Fig. 7a timing and information gain data were combined into a single panel by encoding timing data as color and information gain as luminance (using a squared look-up-table to enhance contrast). Please see Section “Mapping of whole-CNS activity timing” in the Methods for a detailed description of the computations performed to generate this map.

Supplementary Movie 10 Whole-CNS activity timing map for backward locomotor waves
Slice-by-slice visualization of the three-dimensional whole-CNS activity timing map as in Supplementary Movie 9, but for fictive backward locomotor waves (n = 70).
