# Supplementary material for: Whole-central nervous system functional imaging in larval Drosophila
Source: Nat Commun. 2015 Aug 11;6:7924. doi: 10.1038/ncomms8924 (PMC4918770; doi:10.1038/ncomms8924)
Supplement: Supplementary Information — Supplementary Figures 1-8 and Supplementary Methods and Supplementary References [file ncomms8924-s1.pdf]

Whole central nervous system functional imaging in larval *Drosophila*

Supplementary Information

William C. Lemon[1,2], Stefan R. Pulver[1,2], Burkhard Höckendorf[1], Katie McDole[1], Kristin Branson[1], Jeremy Freeman[1] and Philipp J. Keller[1]

[1] *Howard Hughes Medical Institute, Janelia Research Campus, Ashburn, Virginia, USA*

[2] *These authors contributed equally to this work.*

Correspondence should be addressed to P.J.K. (kellerp@janelia.hhmi.org) and S.R.P. (pulvers@janelia.hhmi.org).

Supplementary Figure 1 | Multi-view imaging is essential for optimizing physical coverage of the *Drosophila* CNS

Dorsoventral (first row) and lateral (second row) maximum-intensity projections as well as single image planes (third to fifth rows) from a multi-view image data set of a 3rd instar *Drosophila* CNS with fluorescently labeled cell nuclei. The images captured by each camera for each light sheet are shown separately in the first four columns, and the combined data set produced by both cameras and both light sheets is shown in the fifth column. Strong light scattering and absorption in the *Drosophila* CNS limit the penetration depth of the illuminating light sheets into the specimen. Likewise, fluorescence signal is increasingly difficult to collect at high contrast with increasing imaging depth into the specimen. Camera A has direct optical access to the dorsal side of the CNS and thus captures the dorsal parts of the VNC and brain lobes in high quality. Camera B has direct optical access to the ventral side of the CNS and thus captures the central parts of the VNC and brain lobes in high quality, i.e. the two cameras provide complementary physical coverage of the specimen. Similarly, light sheet A is able to maintain a high-quality profile across one lateral side of the specimen, whereas light sheet B provides good coverage of the other lateral half.

The data shown here represents CNS coverage using one-photon excitation. We note that the situation is effectively the same using two-photon excitation: Fluorescence detection occurs at the same wavelengths when using two-photon excitation, and, by design, the bi-directional light sheets also need to be restricted to one lateral side of the specimen each in order to optimize two-photon excitation efficiency and spatial resolution[1,2].

Scale bars, 50 µm.

Supplementary Figure 1

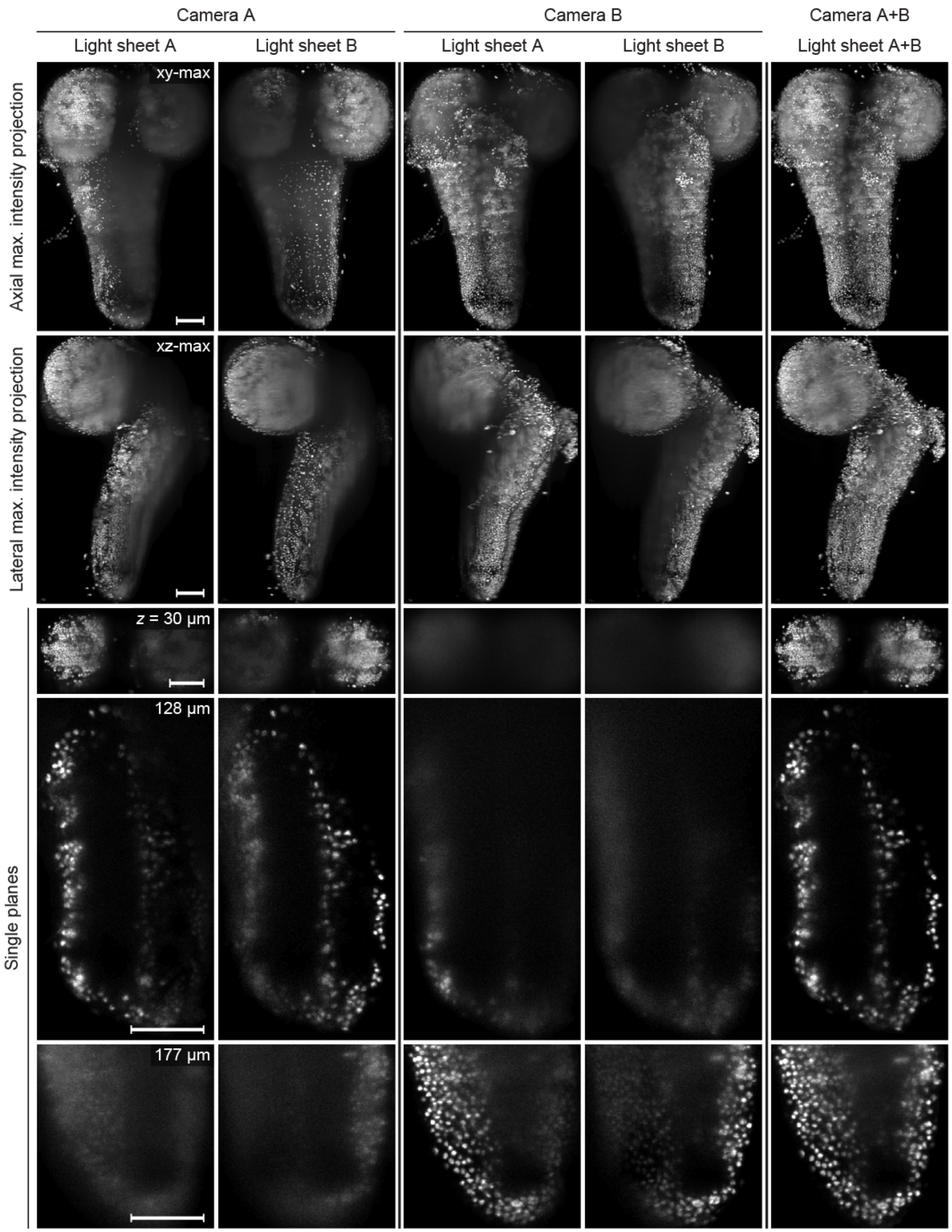

Supplementary Figure 2 | hs-SiMView light-sheet microscope for high-speed multi-view functional imaging

Top panel: Technical design of the high-speed simultaneous multi-view (hs-SiMView) light-sheet microscope for piezo-based functional imaging. Fluorescence excitation is achieved by scanned light-sheet illumination from two opposite directions. Fluorescence detection is performed by orthogonal, bi-directional wide-field detection using two opposing high-numerical-aperture detection objectives and fast sCMOS cameras. Simultaneous volumetric imaging of the stationary specimen from two directions is performed by rapidly sweeping the two scanned light sheets across the sample volume using laser scanners. The focal planes of the detection objectives are co-translated during the volumetric sweep by using spatiotemporally matched high-speed piezo scanning. Thereby, the positions of the two focal planes and the two light sheets are matched throughout the volumetric scan and a complete multi-view data set is obtained with a single imaging pass of the specimen volume.

Bottom panel: Detail view of the microscope core for simultaneous multi-view functional imaging. Each detection arm is equipped with a piezo-stage detection arrangement for fast volumetric imaging. Each arrangement consists of a high-speed piezo stage and a high-numerical-aperture detection objective, as well as a custom Y-Z-θ fine adjustment stage and a custom X-flexure for optimizing three-dimensional position and angular orientation of the 3D imaging volume covered by the respective piezo-positioned detection objective. The four (X-Y-Z-θ) spatial degrees of freedom required for this purpose are distributed over two mechanical sub-systems in order to minimize the load carried by the piezo stage. This load minimization is achieved by positioning the Y-Z-θ fine adjustment stage directly underneath the high-speed piezo stage. The detail view also shows the two illumination objectives for one- or two-photon fluorescence excitation and the respective support stages used to match their optical axes and optimize relative light sheet waist positions (**Supplementary Fig. 3**).

Supplementary Figure 2

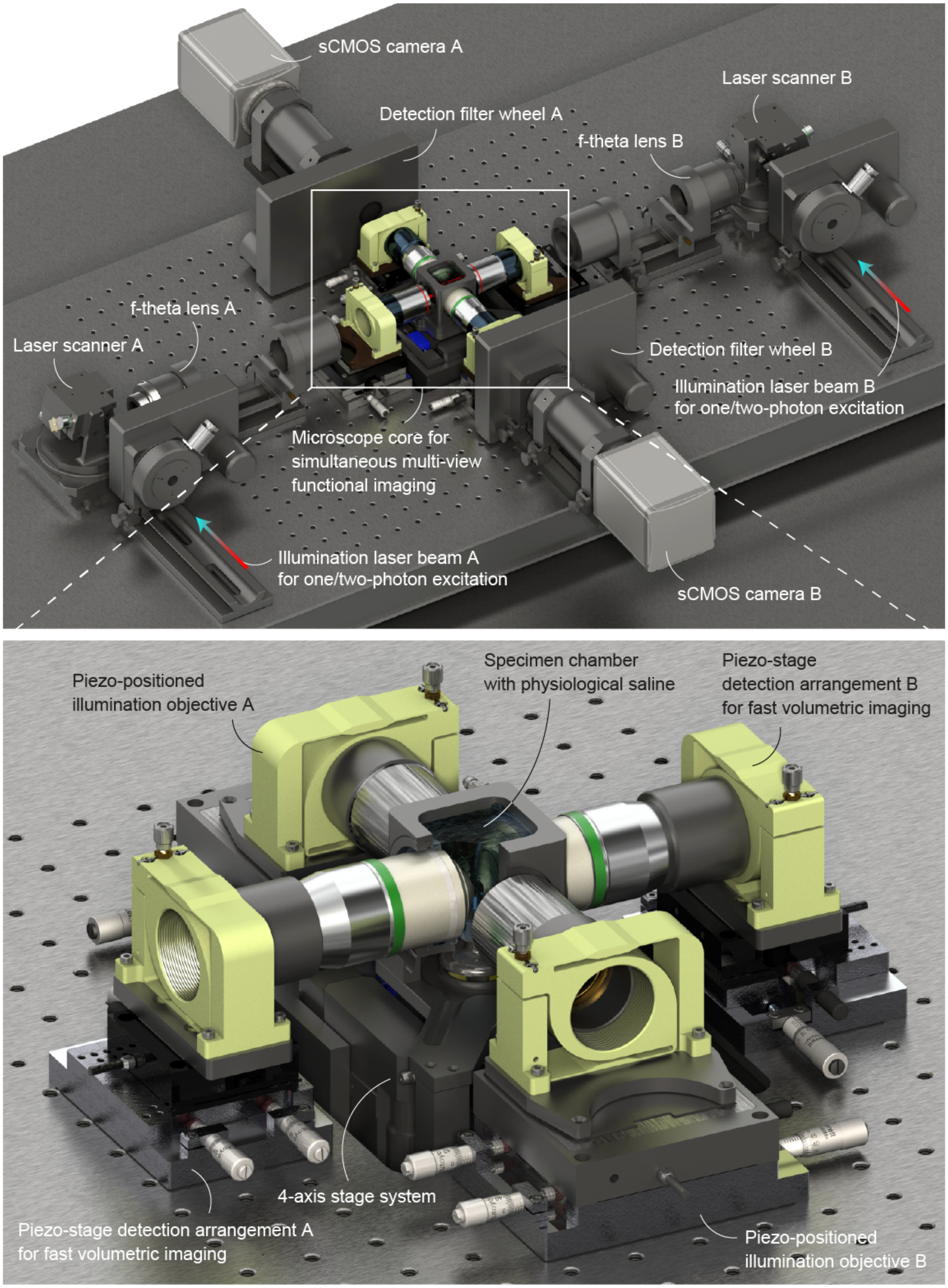

Supplementary Figure 3 | Light-sheet configuration for whole-CNS functional imaging

The lateral dimensions of the field-of-view required for whole-CNS functional imaging in a 3rd instar *Drosophila* larva are approximately 500 µm × 200 µm if the anteroposterior and mediolateral axes of the specimen are oriented parallel to the edges of the rectangular camera field and the dorsoventral axis is oriented perpendicular to the image plane. The combination of this field-of-view configuration with light-sheet illumination along the mediolateral axis of the CNS explant optimizes image quality, imaging speed and physical coverage of the specimen, since it (1) minimizes the sum of illumination and detection light path lengths within the CNS (thus minimizing the effects of light scattering and absorption), (2) favors shorter detection path lengths over shorter illumination path lengths (which is crucial because of the much higher numerical apertures employed in the detection systems), (3) minimizes the depth of the imaging volume, i.e. the dimension along which high-speed piezo scanning is required (thus maximizing volumetric imaging speed) and (4) implements features 1-3 for a specimen orientation that minimizes depth of field along the illumination axis (thus maximizing spatial resolution across the field-of-view). The light-sheet configurations shown in this figure represent theoretically optimal microscope settings for minimizing average light-sheet thickness across the specimen volume in this imaging experiment. In practice, light scattering leads to a significant reduction in spatial resolution compared to this theoretically optimal scenario, and this effect has been analyzed across all non-neuropil regions of the CNS in **Supplementary Figs. 4-6**. The effective axial resolution measurements obtained from these latter experiments provide the data required for determining the optimal trade-off between imaging speed and effective resolution across the CNS.

(**a**) Experimental measurement and fit of light-sheet thickness in water for a theoretically optimal light sheet configuration across a 100 µm wide field-of-view, i.e. across the distance a single light sheet needs to be optimized for in order to cover the size of the entire *Drosophila* CNS with two bi-directional light sheets. Light sheet thickness across the field-of-view ranges from 1.5 to 3.0 µm, i.e. the beam waist was engineered slightly thinner than dictated by the Rayleigh criterion, in order to account for the non-rectangular outline of the *Drosophila* CNS in the optimization process.

(**b**) Dual light-sheet configuration for imaging a field-of-view with a width equivalent to the lateral size of the entire *Drosophila* CNS (200 µm). The minor spherical aberrations evident in the experimental data are the result of limited production quality of the laser fiber collimators and have negligible impact on effective spatial resolution across the field-of-view.

Supplementary Figure 3

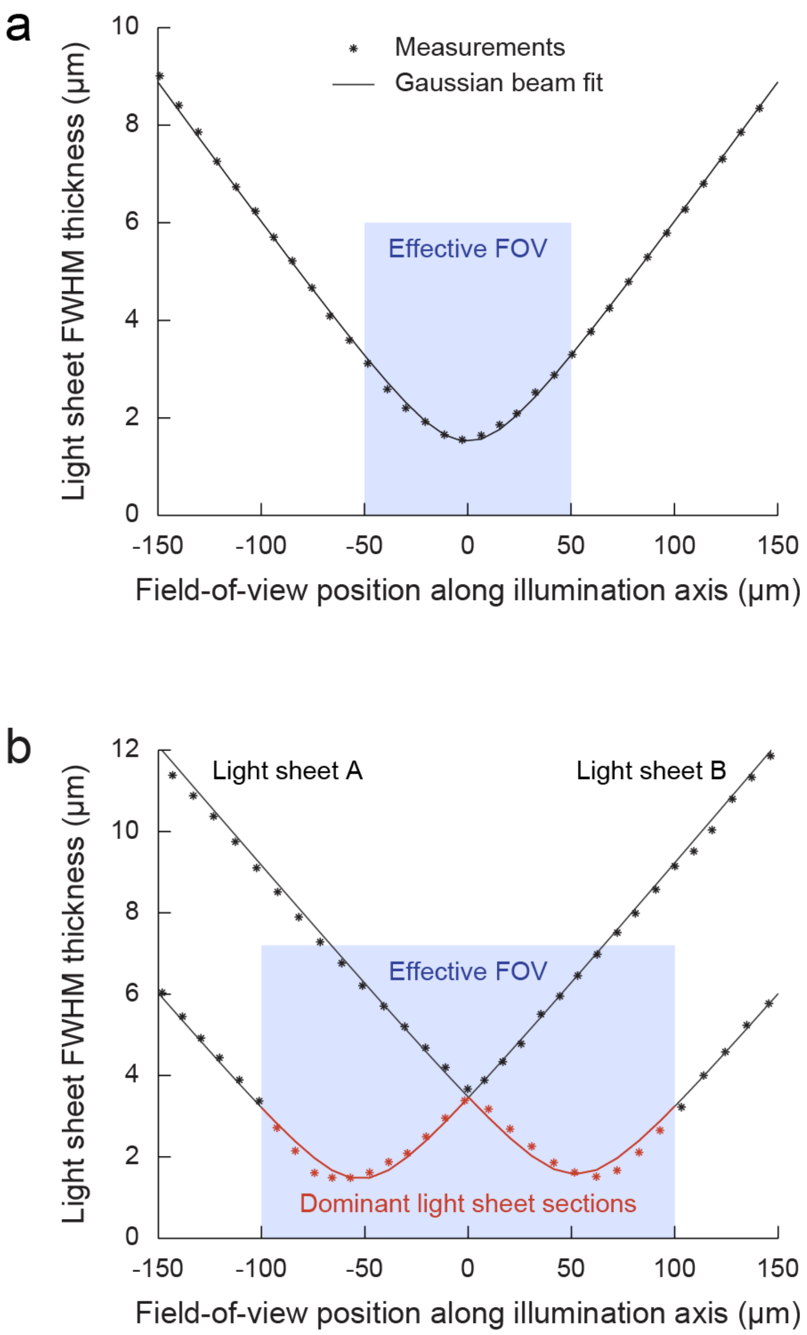

Supplementary Figure 4 | Spatial resolution maps for the *Drosophila* 1st instar larval CNS

(**a**) Maximum-intensity projection of an image stack of a *Drosophila* 1st instar larval CNS expressing the ubiquitous nuclear label His2Av-eGFP, recorded with our light-sheet microscope for multi-view functional imaging. The image was gamma-corrected in order to compensate for heterogeneous expression levels in this transgenic line and to better visualize overall anatomy.
(**b**,**c**) Individual image slices of brain lobes (b) and ventral nerve cord (c) taken from the same image stack visualized in (a). Labels indicate distance of the current image slice from the ventral surface.
(**d**) Estimation of lateral resolution as a function of spatial location in non-neuropil regions of the 1st instar larval CNS. Resolution was estimated locally for each voxel by analyzing the intensity profile measured for the respective closest cell nucleus, following the procedure described in Section "*Spatial Resolution Analysis*" in the **Supplementary Methods**. The images shown here represent slices of the resulting three-dimensional resolution map at different axial positions in the imaging volume (labels indicate distance from the ventral surface).
(**e**) Histogram of lateral resolution achieved throughout non-neuropil regions of the 1st instar larval CNS. Each bar indicates the total volume fraction covered at the respective lateral resolution. Based on the 6.5 µm pixel pitch of the camera chip and the 16× detection magnification, the sampling-limited lateral resolution of 0.81 µm represents the lower physical limit in this resolution map. The average lateral resolution is 1.00 ± 0.41 µm (S.D., n = 2,315 nuclei data points).
(**f**,**g**) Slices (f) and histogram (g) of the respective axial resolution map. The average axial resolution across non-neuropil regions of the 1st instar larval CNS is 2.52 ± 1.34 µm (S.D., n = 2,291 nuclei data points).
Scale bars, 25 µm (a-c), 50 µm (d,f).

Supplementary Figure 4

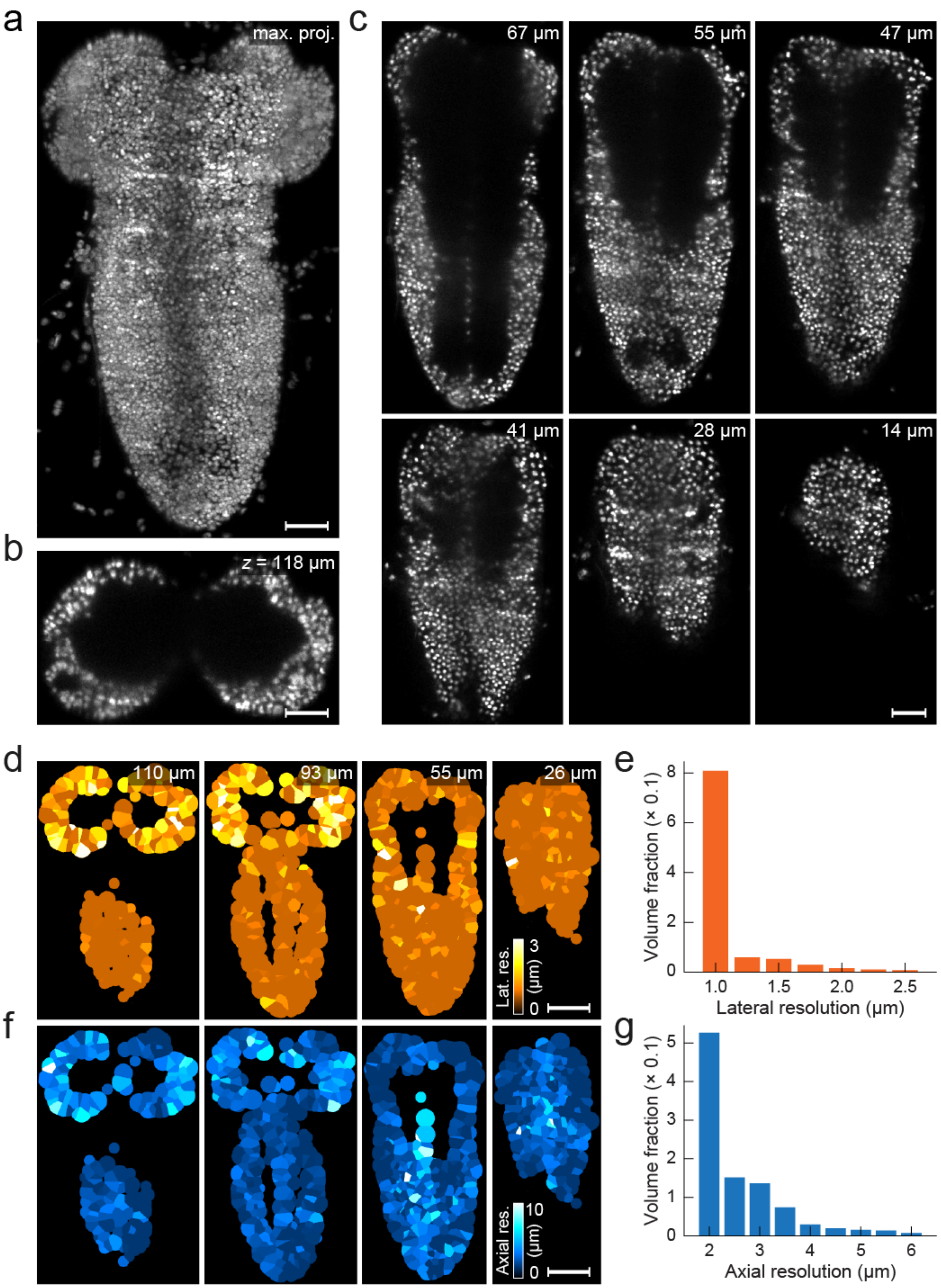

Supplementary Figure 5 | Spatial resolution maps for the *Drosophila* 2nd instar larval CNS

(**a**) Maximum-intensity projection of an image stack of a *Drosophila* 2nd instar larval CNS expressing the ubiquitous nuclear label His2Av-eGFP, recorded with our light-sheet microscope for multi-view functional imaging. The image was gamma-corrected in order to compensate for heterogeneous expression levels in this transgenic line and to better visualize overall anatomy.
(**b**,**c**) Individual image slices of ventral nerve cord (b) and brain lobes (c) taken from the same image stack visualized in (a). Labels indicate distance of the current image slice from the ventral surface.
(**d**) Estimation of lateral resolution as a function of spatial location in non-neuropil regions of the 2nd instar larval CNS. Resolution was estimated locally for each voxel by analyzing the intensity profile measured for the respective closest cell nucleus, following the procedure described in Section "*Spatial Resolution Analysis*" in the **Supplementary Methods**. The images shown here represent slices of the resulting three-dimensional resolution map at different axial positions in the imaging volume (labels indicate distance from the ventral surface).
(**e**) Histogram of lateral resolution achieved throughout non-neuropil regions of the 2nd instar larval CNS. Each bar indicates the total volume fraction covered at the respective lateral resolution. Based on the 6.5 μm pixel pitch of the camera chip and the 16× detection magnification, the sampling-limited lateral resolution of 0.81 μm represents the lower physical limit in this resolution map. The average lateral resolution is 1.05 ± 0.49 μm (S.D., n = 3,328 nuclei data points).
(**f**,**g**) Slices (f) and histogram (g) of the respective axial resolution map. The average axial resolution across non-neuropil regions of the 2nd instar larval CNS is 3.15 ± 1.66 μm (S.D., n = 3,388 nuclei data points).
Scale bars, 50 μm (a-d, f).

Supplementary Figure 5

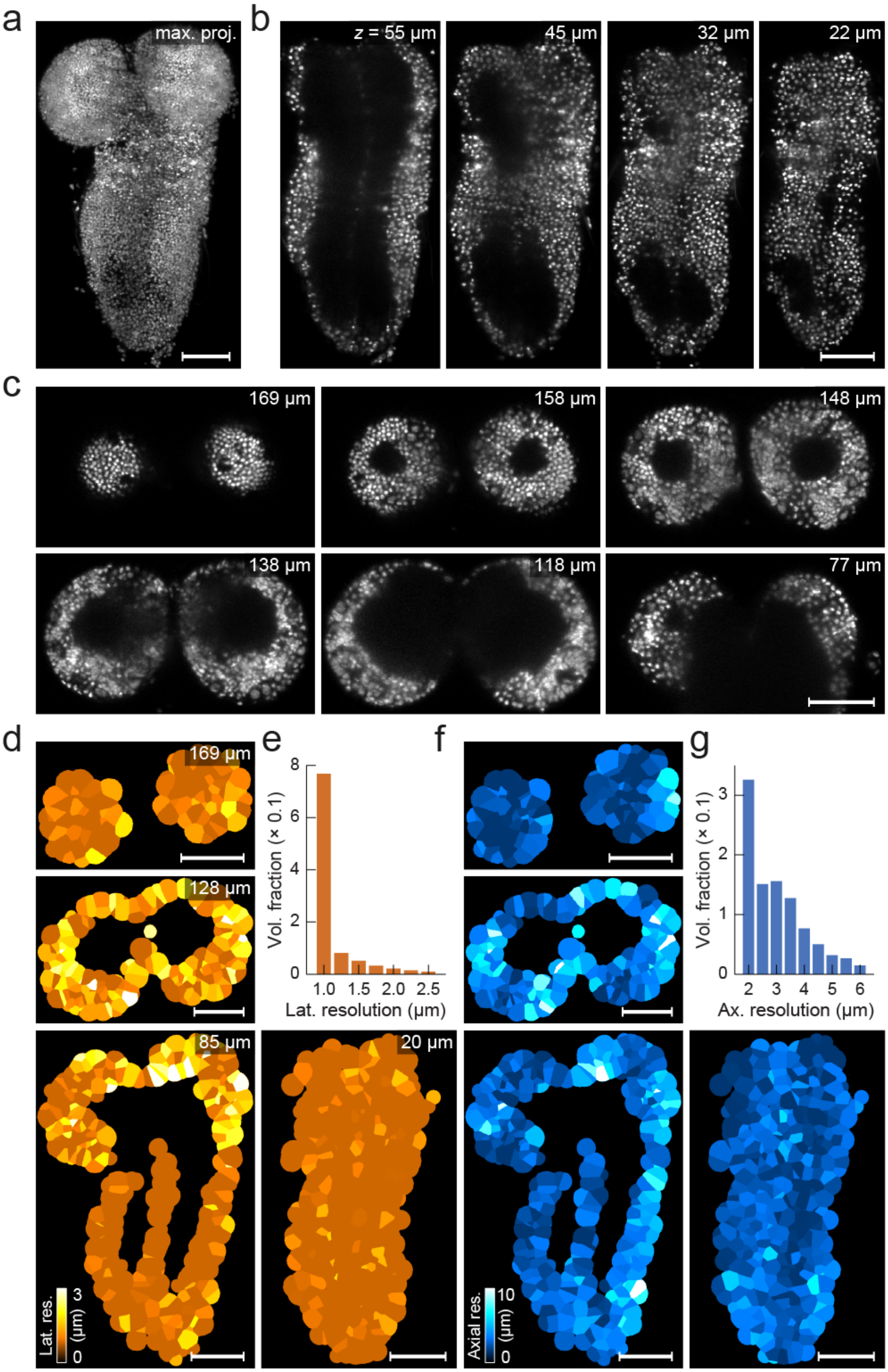

Supplementary Figure 6 | Spatial resolution maps for the *Drosophila* 3rd instar larval CNS

(**a**) Maximum-intensity projection of an image stack of a *Drosophila* 3rd instar larval CNS expressing the ubiquitous nuclear label His2Av-eGFP, recorded with our light-sheet microscope for multi-view functional imaging. The image was gamma-corrected in order to compensate for heterogeneous expression levels in this transgenic line and to better visualize overall anatomy.

(**b-d**) Individual image slices of one of the brain lobes (b) and the ventral nerve cord (c,d) taken from the same image stack visualized in (a). Labels indicate distance of the current image slice from the ventral surface in the top row of panel (b) and in panel (c), and distance from the dorsal surface in the bottom row of panel (b) and in panel (d).

(**e**) Estimation of lateral resolution as a function of spatial location in non-neuropil regions of the 3rd instar larval CNS. Resolution was estimated locally for each voxel by analyzing the intensity profile measured for the respective closest cell nucleus, following the procedure described in Section "*Spatial Resolution Analysis*" in the **Supplementary Methods**. The images shown here represent slices of the resulting three-dimensional resolution map at different axial positions in the imaging volume (labels indicate distance from the ventral surface).

(**f**) Histogram of lateral resolution achieved throughout non-neuropil regions of the 3rd instar larval CNS. Each bar indicates the total volume fraction covered at the respective lateral resolution. Based on the 6.5 µm pixel pitch of the camera chip and the 16× detection magnification, the sampling-limited lateral resolution of 0.81 µm represents the lower physical limit in this resolution map. The average lateral resolution is 1.57 ± 0.94 µm (S.D., n = 12,884 nuclei data points).

(**g,h**) Slices (f) and histogram (g) of the respective axial resolution map. The average axial resolution across non-neuropil regions of the 3rd instar larval CNS is 5.91 ± 3.58 µm (S.D., n = 14,938 nuclei data points).

Scale bars, 25 µm (b-d), 50 µm (a,e,g).

Supplementary Figure 6

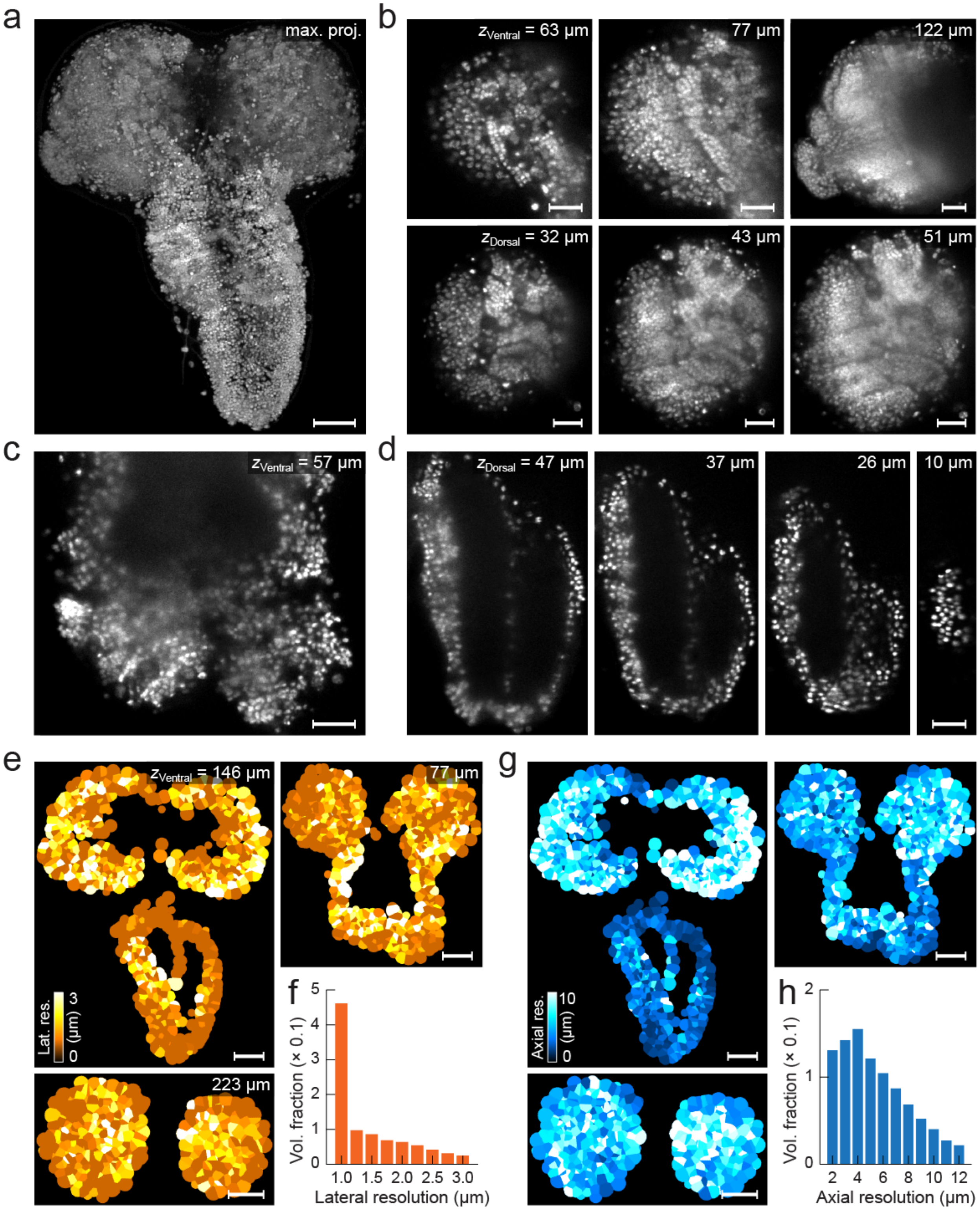

Supplementary Figure 7 | Single-neuron activity traces during backward locomotor waves

(**a**) Projection of manually annotated three-dimensional somatic regions of interest in the larval CNS shown in **Supplementary Movies 3-5**. Functional imaging in this experiment was performed with two-photon multi-view light-sheet microscopy. Soma annotations throughout the CNS were used for extracting signals during backward locomotor waves (n = 26) at the single-neuron level (panel (b), n = 688). Traces of neurons from the three groups highlighted in yellow outlines (1a to 1c, 2a to 2c and 3a to 3c) are shown in panel (c).
(**b**) Neuronal activity of the 688 somas shown in panel (a), averaged across all detected backward wave time windows. Left/right panels shown raw/normalized $\Delta F/F$ (grayscale) for a 20-second time window centered on the locomotor waves in the VNC. Matrices above show somatic responses (color bar to the right indicates location as shown in panel (a)). Matrices below show corresponding VNC signals (A1 to A8, averaged across left and right hemisegments).
(**c**) Single-neuron traces for all 26 wave events for the three groups of neurons highlighted in panel (a). Left/right panels in each of the three columns show raw/normalized $\Delta F/F$ (grayscale) for a 20-second time window centered on the locomotor waves in the VNC. Group 1 (neurons 1a to 1c, first column) shows neurons at the posterior end of the VNC with a distinct peak in activity at the end of the locomotor waves. Group 2 (neurons 2a to 2c, second column) shows neurons slightly more anterior with strong increase in activity towards the beginning of the locomotor waves and subsequent maintenance of the high activity level for an extended period of time. Group 3 (neurons 3a to 3c, third column) shows neurons at the boundary between abdomen and thorax with strong reduction in activity at around the middle of the locomotor waves.
Scale bar, 25 μm (a).

Supplementary Figure 7

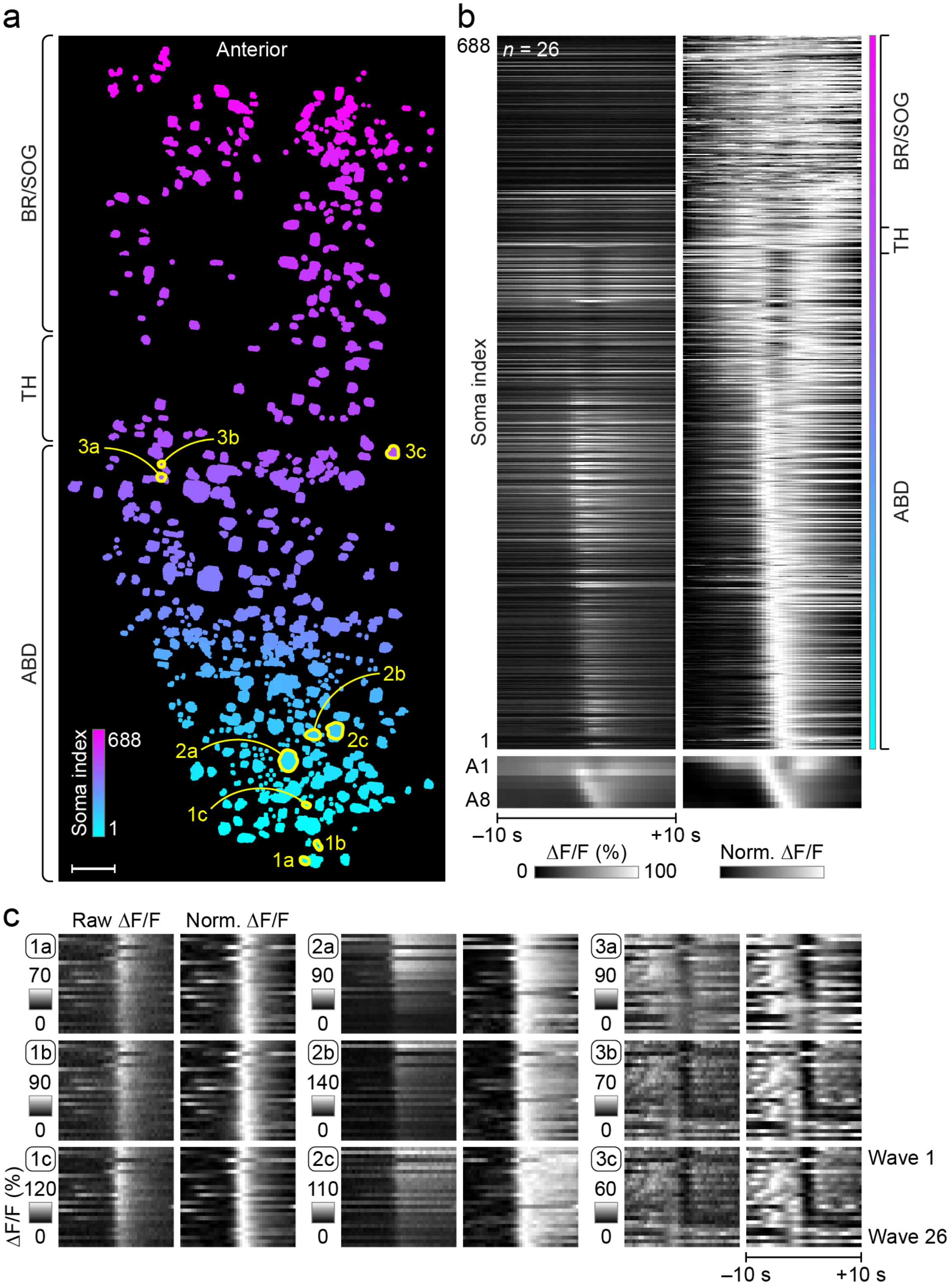

Supplementary Figure 8 | Photobleaching in long-term whole-CNS functional imaging

The plot shows normalized GCaMP baseline intensity (solid lines correspond to average values, shaded areas to standard deviation across the entire CNS) as a function of time during time-lapse volumetric functional imaging. Over the course of 60 min of whole-CNS imaging with one-photon excitation (488 nm excitation wavelength) at 5 Hz, average GCaMP baseline intensity dropped to 78.4% of the initial level. Over the course of 57 min of whole-CNS imaging with two-photon excitation (940 nm excitation wavelength) at 2 Hz, average GCaMP baseline intensity dropped to 54.5% of the initial level. The ratio of fluorescence signal rates was 1.66 to 1 for one-photon vs. two-photon imaging at the onset of the time-lapse imaging experiment.

Supplementary Figure 8

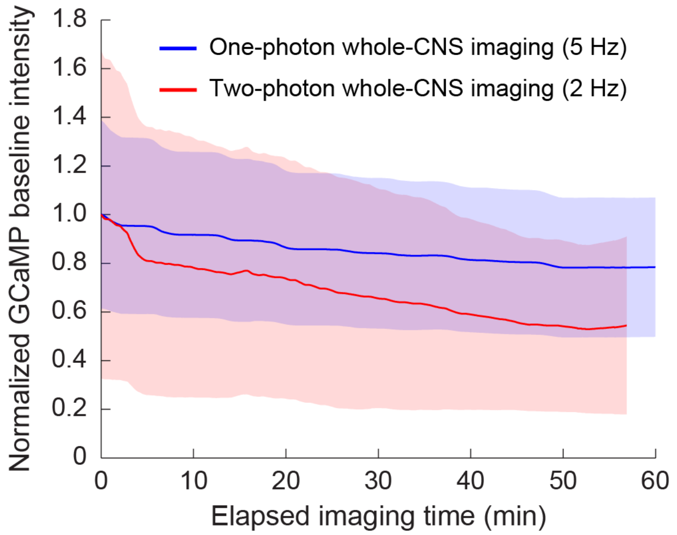

Supplementary Methods

Components of the hs-SiMView Light-Sheet Microscope

In addition to the new hs-SiMView microscope core for multi-view functional imaging, which consists of two piezo-stage detection arrangements, two piezo-stage illumination arrangements, a custom specimen chamber with four flexible, rubber-based objective ports and a four-axis specimen positioning system (please see **Supplementary Data 1** for technical drawings of critical custom components), our custom light-sheet microscope includes a laser illumination unit, two illumination arms, two detection arms, computational hardware for high-speed multi-view image acquisition, a real-time electronics control framework and custom LabVIEW-based software for microscope control. A brief description of these latter modules, which share components with earlier microscope designs[2, 3], is provided below.

The laser illumination unit includes a pulsed Ti:Sapphire laser (Chameleon Ultra II, Coherent), beam-splitting optics (Melles Griot, Linos and Thorlabs) for supplying the two independent scanned light-sheet illumination arms with IR laser beams in a continuously adjustable splitting ratio, a laser array with multiple solid-state and DPSS lasers coupled into a dual-port fiber module (SOLE-6, Omicron), fiber collimators for interfacing with the microscope's illumination arms and dichroic mirrors for directing the visible and infrared laser beams onto the same beam axis in each illumination arm.

Each illumination arm consist of a laser shutter (VS14S2ZM1-100, Uniblitz), a filter wheel (96A351, Ludl) equipped with notch and neutral density filters (Chroma and Melles Griot), a two-axis galvanometer scanner with silver-coated 6 mm mirrors (Cambridge Technology), a custom f-theta lens supporting 488-1100 nm laser wavelengths (66-S80-30T-488-1100nm, Special Optics) and two sets of matched tube lenses and low numerical aperture illumination objectives (Olympus and Nikon; for one- and two-photon excitation, respectively, see below).

Each detection arm consists of a high numerical aperture water-dipping detection objective (16x/0.8 NA, Nikon), a matched tube lens (Nikon), a filter wheel (96A354, Ludl) equipped with band-, short- and long-pass filters (Semrock and AHF), and an sCMOS camera (Orca Flash 4.0 V2, Hamamatsu).

The computational framework for high-speed multi-view image acquisition consists of a high-performance computer workstation for image acquisition and short-term data storage and a high-

capacity server for long-term data storage. The image acquisition workstation is equipped with two Xeon E5-2687W CPUs (Intel) for high-speed dual-sCMOS operation, 192 GB of memory allocated to image ring buffers, 14 SAS hard disks (2.5 XE 900 GB, Western Digital) combined into two RAID-0 arrays using an RS2WG160 SAS RAID controller (Intel) for concurrent streaming from two high-speed cameras and an X520-SR1 10G fiber controller (Intel) for data offloading to the storage server. The storage server is equipped with two Xeon E5649 CPUs (Intel), 96 GB of memory, 36 SATA hard disks (A7K4000 4 TB, Hitachi) combined into two RAID-6 arrays using a 9261-8i RAID controller (LSI) and an X520-SR1 10G fiber controller (Intel).

The real-time electronics framework consists of a PXI-8110 real-time controller equipped with four PXI-6733 high-speed analog output boards, a PXI-8432/2 serial interface board and a PXI-7354 four-axis stepper/servo motion controller (National Instruments), as well as four summing amplifiers (SIM980 in SIM900 mainframe, Stanford Research Systems), a C-809.40 four-channel servo amplifier (Physik Instrumente) and four BNC-2110 shielded connector blocks (National Instruments).

The custom microscope control software was written in LabVIEW (National Instruments) and consists of a 32-bit software layer for communicating with the real-time OS on the PXI-8110 real-time controller (for hardware control and synchronization) and a 64-bit software layer for image acquisition, data streaming and GUI-based user interaction.

High-Speed Volumetric Functional Imaging

Our multi-view microscope offers two approaches to acquiring volumetric data using this arrangement. The first method holds the position of the detection objectives and light-sheets constant and moves the specimen through the focal plane. The acquisition speed in this method is limited by the speed of the mechanical stages that move the specimen and by the mechanical and physiological robustness of the specimen with respect to physical movement[2]. For calcium imaging, the acquisition rate needs to be as fast as possible, so we used the second method, which holds the specimen stationary and makes coordinated movements of the light sheets and detection objectives to move the focal planes through the volume of the specimen. With this method the image acquisition rate is effectively limited only by the frame rate of the cameras. In turn, the frame rate of the camera can be increased by reducing the size of the region of interest (ROI). In our case, with the ROI matched to the size of the larval CNS, the maximum theoretical frame rate

per camera was about 205 Hz. In practice, we allow 20 ms at the end of each z-stack acquisition for the piezoelectric positioners to move the detection objectives from the last plane of a stack to the first plane of the next stack, which reduces the maximum image acquisition rate to 185 Hz per camera.

The isolated *Drosophila* larval CNS was positioned in the center of the field of view of the cameras with the dorsal and ventral surfaces facing the cameras. Using the custom LabVIEW microscope control software 6-8 reference planes were then placed across the specimen, covering both dorsal and ventral parts of the specimen. At each reference plane, focus and light sheet positions required for optimal image quality were measured, using the high-precision piezo stages to define a continuous, linear reference coordinate system. A look-up-table for optimal parameter settings, ensuring co-planarity of both light sheets and both focal planes across the entire specimen, was constructed from these data points and used to optimize image quality during volumetric time-lapse imaging. The volume of the typical third instar CNS was approximately 500 µm x 200 µm x 200 µm, i.e. the 200 µm thickness of the CNS was just within the 250 µm travel range of the piezoelectric positioners carrying the detection objectives. We chose to set the z-step size at 4-6 µm so that each of the neuronal cell bodies would be represented in more than one plane. This setting furthermore considers that the average effective axial resolution across the 3rd instar CNS is 5.9 µm as a result of light scattering (**Supplementary Fig. 6**), i.e. a z-step size significantly smaller than 4 µm would primarily lead to reduced temporal resolution with little improvement in effective spatial resolution. This combination of parameters results in a z-stack containing on average 37 planes per camera that, at 185 Hz, are acquired in 200 ms. The resulting volumetric images are thus acquired at a sustained rate of 5 Hz with cellular resolution for a large fraction of the CNS (**Supplementary Figs. 1** and **3-6**). In our imaging experiments with one-photon excitation, full-volume z-stacks were typically acquired at 200 ms intervals (5 Hz) for 60 min (**Supplementary Movies 1** and **2**). In this setting, the two weakly focused 488 nm laser beams are simultaneously scanned across the sample within 1 ms, using the bi-directional rolling shutter mode of the sCMOS cameras with a 4 ms exposure time per image. Some of the specimens in this study were recorded at 400 ms full-volume z-stack intervals (2.5 Hz), using the same spatial sampling as in the higher-speed recordings. In our imaging experiments with two-photon excitation, full-volume z-stacks were typically acquired at 500 ms intervals (2 Hz) for 60 min (**Supplementary Movies 3-5**), which

produced the best compromise between temporal resolution, signal rate and long-term imaging capability in this scenario (**Supplementary Fig. 8**).

Multi-view imaging provides a key advantage when recording from thick specimens (**Supplementary Fig. 1**). Since each plane is imaged with two cameras from opposite sides, fluorescent light never has to be transmitted through more than half of the thickness of the specimen to reach one of the cameras, which consequently reduces the effect of light scattering caused by the tissue. For each location in the specimen, image data from the better of the two images acquired at the respective plane is retained and these data are compiled into a single z-stack. The resulting volumetric image data has the best resolution at the first and last planes and reduced resolution at planes closer to the center of the specimen, where light scattering along the detection axis is greatest.

Illumination by multiple light sheets provides a similar advantage. As they travel through the specimen, the light sheets are subjected to refraction, absorption and scattering, which leads to aberrations as well as a broadening of the sheets to a larger extent than would be expected for undisturbed Gaussian beams. Thus, the light sheet entering the specimen from the left provides the best spatial resolution and image quality for the specimen's left side while the second light sheet, entering from the right, is best-suited to illuminate the specimen's right side (**Supplementary Fig. 1**). In principle, the best resolution images can be generated by alternately illuminating each plane by one light sheet at a time and then fusing the two images of the plane into a single image. However, the added time required to illuminate the specimen twice at each plane makes this method impractical for high-speed calcium imaging. Instead, we simultaneously illuminated the entire specimen with light sheets from both sides of the specimen. This approach produces images with slightly lower contrast in some regions of the specimen, as the light sheets are scattered as they pass through the specimen and weakly illuminate a thicker slice of the tissue on the far side of the specimen[3], but it also leads to a substantial increase in volumetric imaging speed.

At the conclusion of each GCaMP6s functional imaging experiment, we imaged the larval CNS using a single light sheet at a time. This provided a reference image of the GCaMP labeled CNS with the best possible quality, and was used for spatial registration of all recorded specimens. We repeated this high-quality imaging with a 561 nm laser, which excited the tdTomato reporter expressed in the mushroom bodies and neuropil of the VNC. This red label served as a consistent

landmark between specimens that allowed reliable identification of brain regions and segments of the VNC.

Since the light-sheet microscope illuminates only the plane being imaged and integrates signal over millisecond time-scales for each frame, using low laser power, photo-damage and photo-bleaching are greatly reduced as compared to conventional point-scanning or wide-field microscopy. We found that long recordings of calcium signals can be obtained with low rates of loss of fluorescent signal or changes in the SNR when using one-photon excitation and only slightly higher rates of signal loss for two-photon excitation (**Supplementary Fig. 8**). In this study, we present the results of recordings that typically lasted 60 min, although we have successfully recorded at 5 Hz from *Drosophila* larvae and embryos for up to 4 h.

Spatial Resolution Analysis

To quantitatively assess spatial resolution as a function of the spatial location within the specimen, we imaged nervous systems of 1st, 2nd and 3rd instar animals with a ubiquitous nuclear label (w; His2Av-eGFP; +), using the same microscope configuration as for imaging the GCaMP6s-labeled nervous systems. By labeling all cell nuclei in the animal, the nuclear marker effectively provided blob-like targets suitable for lateral and axial resolution measurements throughout all non-neuropil regions of the CNS and thus allowed us to estimate spatial resolution *in situ* under relevant experimental conditions. Volumetric imaging was performed with an axial step size of 2.0 µm. Three-dimensional spatial resolution maps were then computationally reconstructed from the volumetric images using the automated procedure described below:

In the first step, a blob detector optimized for anisotropic volumetric image data of fluorescently-labeled cell nuclei[4, 5] was applied to the fused multi-view image data sets in order to estimate three-dimensional nuclei centroid positions across the specimen. The detector was tuned to yield low false positive rates in order to maximize data fidelity and minimize the need for object filtering in subsequent steps.

In the second step, using the centroids estimated by the blob detector as initial seeds, a local search with anisotropic search radius matched to the sampling aspect ratio in the image data was performed for each seed position to identify the brightest region in the local voxel neighborhood. Searches that converged on already identified regions were discarded to avoid redundancy.

In the third step, one-dimensional local intensity profiles centered on the voxels identified in the second step were analyzed along the x-, y- and z-axes (with x and y corresponding to the lateral image dimensions and z to the axial image dimension) and σ values of Gaussian curves best describing the observed intensity profiles were determined along all axes and measured separately for positive and negative directions along each axis. In order to obtain lateral and axial full-width half-maximum (FWHM) sizes for each analyzed nucleus, the median value of the four σ values characterizing the x- and y-profiles and the mean value of the two σ values characterizing the z-profile were determined and converted to FWHM measures. To maximize the quality of the pool of data points resulting from this analysis only data points with robust estimates for all four lateral σ values and/or both axial σ values were considered further.

In the fourth step, conservative local resolution estimates were extracted from the local FWHM size measurements by subtracting the average real nucleus size while enforcing theoretical resolution limits arising from the finite lateral sampling and light-sheet thickness. For this purpose, the average real nucleus size was determined as follows. Cell nuclei characterized in the third step and located in the most superficial regions of the specimen (i.e. imaged at diffraction- and sampling-limited resolution owing to the negligible optical path length inside the specimen) were identified and their measured lateral FWHM sizes were averaged. This resulted in average FWHM sizes of 2.47 ± 0.41 µm for 1st instar animals (S.D., n = 158 nuclei), 2.37 ± 0.58 µm for 2nd instar animals (S.D., n = 266 nuclei) and 2.39 ± 0.89 µm for 3rd instar animals (S.D., n = 161 nuclei). These numbers were corrected for the increase in measured object size corresponding to a diffraction-limited resolution of 0.39 µm in order to arrive at an estimate of the average real nucleus size. The average real nucleus size (2.08 µm for 1st instar, 1.98 µm for 2nd instar and 2.00 µm for 3rd instar) was then subtracted from the lateral and axial FWHM size measurements obtained above. Finally, lower resolution limits corresponding to the sampling-limited lateral resolution (0.81 µm) and the central light-sheet thickness (1.50 µm) were applied to select against local resolution underestimates resulting from measurement noise and the non-zero standard deviation of the real nucleus size across the CNS.

The final measurement vectors, containing lateral and axial resolution estimates for several thousand spatial coordinates inside the specimen volume, were used to generate three-dimensional resolution maps (**Supplementary Figs. 4-6**) and global statistics of spatial resolution in non-neuropil CNS regions of 1st, 2nd and 3rd instar animals. Statistics were computed using volume-

weighting (i.e. weighting each data point by the number of voxels inside the specimen volume for which the given data point represented the closest point in space among all measurement vectors, not exceeding a total distance of 30 pixels or 12 μm) to avoid data bias resulting from non-uniform sampling of data points across the specimen volume. The average lateral resolution estimate across non-neuropil CNS regions resulted as 1.00 ± 0.41 μm for 1st instar animals (S.D., n = 2,315), 1.05 ± 0.49 μm for 2nd instar animals (S.D., n = 3,328) and 1.57 ± 0.94 μm for 3rd instar animals (S.D., n = 12,884). The average axial resolution estimate across non-neuropil CNS regions resulted as 2.52 ± 1.34 μm for 1st instar animals (S.D., n = 2,291), 3.15 ± 1.66 μm for 2nd instar animals (S.D., n = 3,388) and 5.91 ± 3.58 μm for 3rd instar animals (S.D., n = 14,938).

Supplementary References

1. Truong, T.V., Supatto, W., Koos, D.S., Choi, J.M. & Fraser, S.E. Deep and fast live imaging with two-photon scanned light-sheet microscopy. *Nat Methods* **8**, 757-760 (2011).
2. Tomer, R., Khairy, K., Amat, F. & Keller, P.J. Quantitative high-speed imaging of entire developing embryos with simultaneous multiview light-sheet microscopy. *Nat Methods* **9**, 755-763 (2012).
3. Ahrens, M.B., Orger, M.B., Robson, D.N., Li, J.M. & Keller, P.J. Whole-brain functional imaging at cellular resolution using light-sheet microscopy. *Nat Methods* **10**, 413-420 (2013).
4. Santella, A., Du, Z., Nowotschin, S., Hadjantonakis, A.K. & Bao, Z. A hybrid blob-slice model for accurate and efficient detection of fluorescence labeled nuclei in 3D. *BMC Bioinformatics* **11**, 580 (2010).
5. Keller, P.J. et al. Fast, high-contrast imaging of animal development with scanned light sheet-based structured-illumination microscopy. *Nat Methods* **7**, 637-642 (2010).
