# Supplementary material for: Whole-central nervous system functional imaging in larval Drosophila
Source: Nat Commun. 2015 Aug 11;6:7924. doi: 10.1038/ncomms8924 (PMC4918770; doi:10.1038/ncomms8924)
Supplement: Supplementary Data 1 — Technical drawings of individual components and complete assemblies of custom four-axis stage and flexure systems in the hs-SiMView light-sheet microscope [file ncomms8924-s2.zip › Assemblies/Assembly - 3-axis Piezo Mount Small.pdf]

REVISION HISTORY				
ZONE	REV	DESCRIPTION	DATE	APPROVED
	0	DO NOT FABRICATE		

GENERAL NOTES:
1. MATERIAL:
2. SPECIAL FINISH: **NONE**
3. SURFACE ROUGHNESS (UNLESS SPECIFIED OTHERWISE): **N/A** (AVERAGE MICRO-INCHES)
4. INTERPRET DIMENSIONS AND TOLERANCES PER ASME Y14.5M-1994
5. DEBURR AND BREAK ALL SHARP EDGES, MAX 0.010" (UNLESS SPECIFIED OTHERWISE)
6. PARTS ARE TO BE CLEAN AND FREE OF OIL, GREASE, AND OTHER CONTAMINANTS
7. DIMENSIONS INCLUDE ANODIZING, ELECTROPLATING, AND CHEMICALLY APPLIED FINISHES IF APPLICABLE

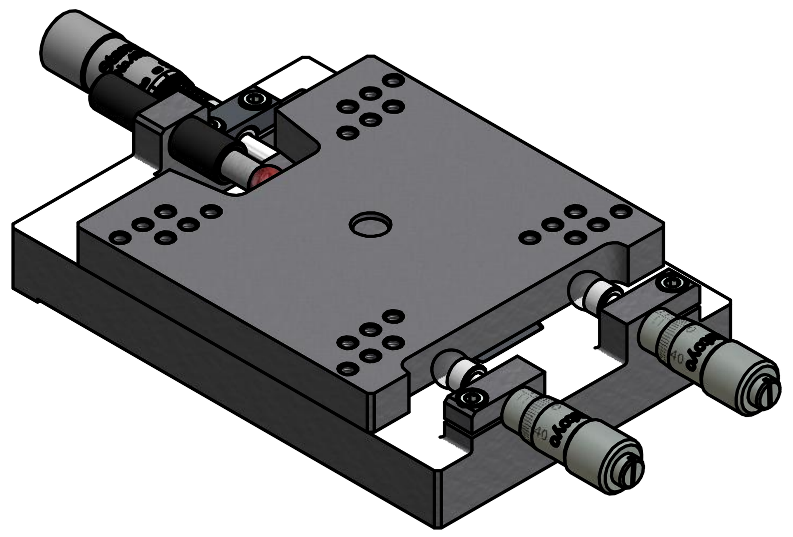

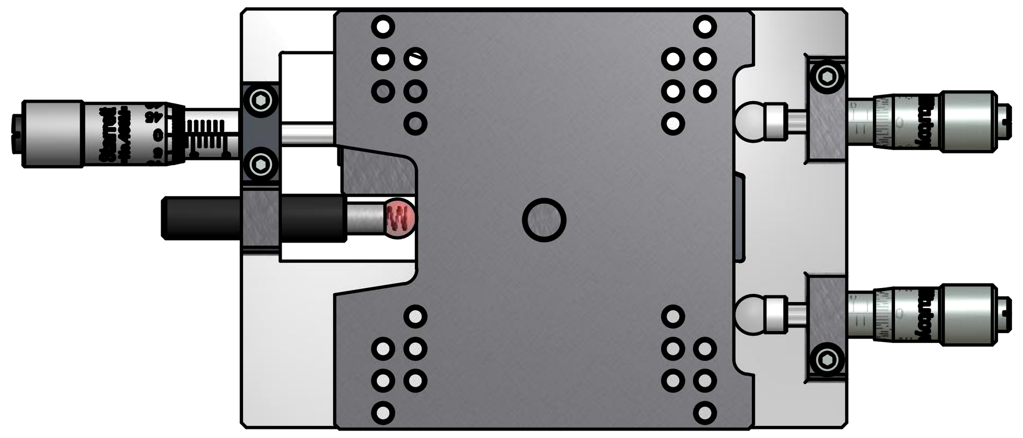

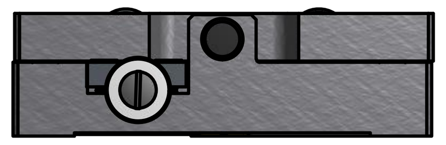

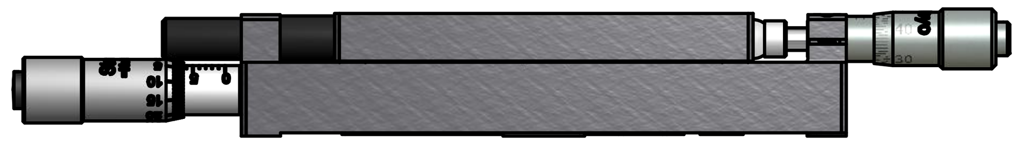

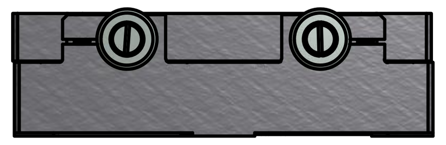

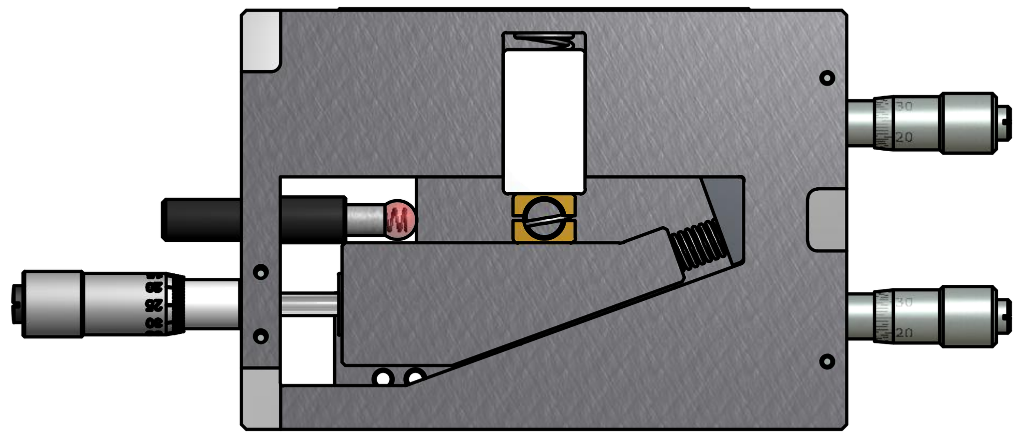

PARTS LIST

ITEM	QTY	FILE NAME	REV	PART NUMBER	VENDOR	DESCRIPTION	A.I. PART #	A.I. VENDOR
1	1	11306.ipt	-		CENTURY SPRING	COIL SPRING	N/A	N/A
2	1	71079S.ipt	-		CENTURY SPRING	COMPRESSION SPRING	N/A	N/A
3	2	MICROMETER TIP.ipt	-	43-829	EDMUND OPTICS	MICROMETER TIP, SAPPHIRE, 6mm DIA	N/A	N/A
4	1	BASE PLATE SMALL.ipt	A	J002552	HHMI: JFRC - ID&F	3-AXIS BASE PLATE	N/A	N/A
5	1	FOCUS WEDGE SMALL.ipt	A	J002553	HHMI: JFRC - ID&F	FOCUS WEDGE, SMALL	N/A	N/A
6	2	PIVOT BUSHING.ipt	A	J002554	HHMI: JFRC - ID&F	SPLIT PIVOT BUSHING	N/A	N/A
7	1	FOCUS SPRING PLUNGER SMALL.ipt	-	J002555	HHMI: JFRC - ID&F	FOCUS SPRING PLUNGER, SMALL	N/A	N/A
8	1	PI-HERA PLATE SMALL.ipt	-	J002556	HHMI: JFRC - ID&F	PI-HERA ADJUSTABLE PLATE	N/A	N/A
9	1	FOCUS MIC CLAMP.ipt	-	J002557	HHMI: JFRC - ID&F	FOCUS MIC CLAMP	N/A	N/A
10	2	SAPPHIRE BALL MIC MOUNT.ipt	A	J002558	HHMI: JFRC - ID&F	SAPPHIRE BALL MIC MOUNT	N/A	N/A
11	1	SPRING PLUNGER.iam	-	J002560	HHMI: JFRC - ID&F	SPRING PLUNGER ASSEMBLY	N/A	N/A
12	1	WEDGE SPRING LOCATOR - SMALL.ipt	-	J002815	HHMI: JFRC - ID&F	WEDGE SPRING LOCATOR - SMALL	N/A	N/A
13	4	MCMASTER 58605K31.ipt	-	58605K31	MCMASTER	DISC MAGNET, NEODYMIUM-IRON-BORON, 1/4" DIA X 0.1" THICK	N/A	N/A
14	4	90128A183.ipt	0	90128A183	MCMASTER	SHCS, M3 X 0.5 X 6mm	N/A	N/A
15	1	98380A539.ipt	0	98380A539 -	MCMASTER	DOWEL PIN, 1/4 X 5/8"	N/A	N/A
16	1	460MB+A.iam	0	460MB+A	STARRETT	MICROMETER HEAD, 12MM	N/A	N/A
17	3	SAPPHIRE WINDOW - W9.58.ipt	-	W9.58	SWISS JEWEL	SAPPHIRE WINDOW	N/A	N/A
18	3	P25SK2.ipt	0		THORLABS	SAPPHIRE DISC	N/A	N/A
19	2	MITUTOYO 148-201FT.iam	0	148-201FT	THORLABS	MITUTOYO MICROMETER HEAD	N/A	N/A

NOTICE:
INFORMATION CONTAINED IN THIS DOCUMENT OR ANY REPRODUCTION THEREOF, IS PROPRIETARY INFORMATION AND PROPERTY OF HOWARD HUGHES MEDICAL INSTITUTE. IT SHALL NOT BE DISCLOSED, COPIED, DUPLICATED OR USED FOR MANUFACTURE, PRODUCTION OR PROCUREMENT, WITHOUT THE EXPRESS WRITTEN PERMISSION OF HOWARD HUGHES MEDICAL INSTITUTE.

(UNLESS SPECIFIED OTHERWISE)
PRIMARY UNITS: INCHES
[SECONDARY UNITS]: MILLIMETERS

PRIMARY TOLERANCES:

X.X	± 0.020
X.XX	± 0.010
X.XXX	± 0.005
X.XXXX	± 0.0005
ANGULAR	± 0.5 DEG

- DO NOT SCALE DRAWING -

THIRD ANGLE PROJECTION:

hhmi Howard Hughes Medical Institute
hhmi janelia Research Campus

3-AXIS PIEZO MOUNT SMALL.iam

SIZE	PART NUMBER	REV	SHEET
D	J002551	0	1 OF 1
