# Supplementary material for: Whole-central nervous system functional imaging in larval Drosophila
Source: Nat Commun. 2015 Aug 11;6:7924. doi: 10.1038/ncomms8924 (PMC4918770; doi:10.1038/ncomms8924)
Supplement: Supplementary Data 1 — Technical drawings of individual components and complete assemblies of custom four-axis stage and flexure systems in the hs-SiMView light-sheet microscope [file ncomms8924-s2.zip › Assemblies/Assembly - Flexure for hs-SiMView Illumination Arm.pdf]

REVISION HISTORY				
ZONE	REV	DESCRIPTION	DATE	APPROVED
	0	DO NOT FABRICATE		

GENERAL NOTES:
1. MATERIAL:
2. SPECIAL FINISH: **NONE**
3. SURFACE ROUGHNESS (UNLESS SPECIFIED OTHERWISE): **N/A** (AVERAGE MICRO-INCHES)
4. INTERPRET DIMENSIONS AND TOLERANCES PER ASME Y14.5M-1994
5. DEBURR AND BREAK ALL SHARP EDGES, MAX 0.010" (UNLESS SPECIFIED OTHERWISE)
6. PARTS ARE TO BE CLEAN AND FREE OF OIL, GREASE, AND OTHER CONTAMINANTS
7. DIMENSIONS INCLUDE ANODIZING, ELECTROPLATING, AND CHEMICALLY APPLIED FINISHES IF APPLICABLE

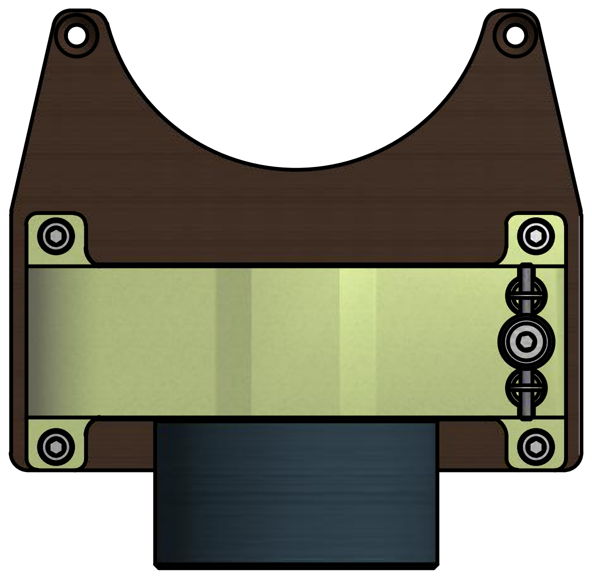

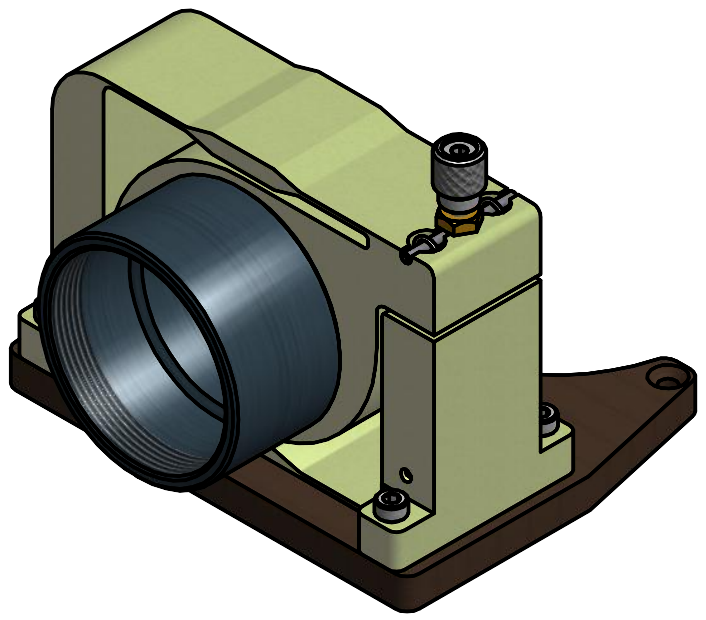

FOR OLYMPUS 4X OBJECTIVE

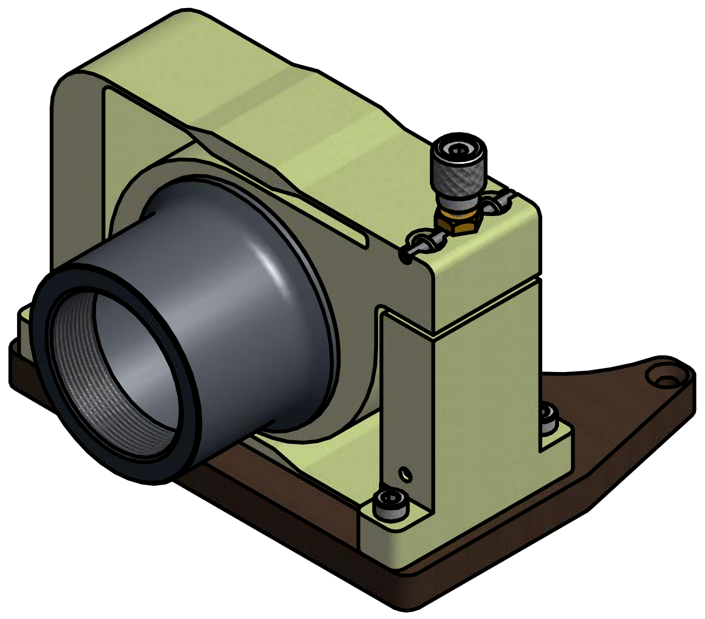

FOR NIKON 10X OBJECTIVE

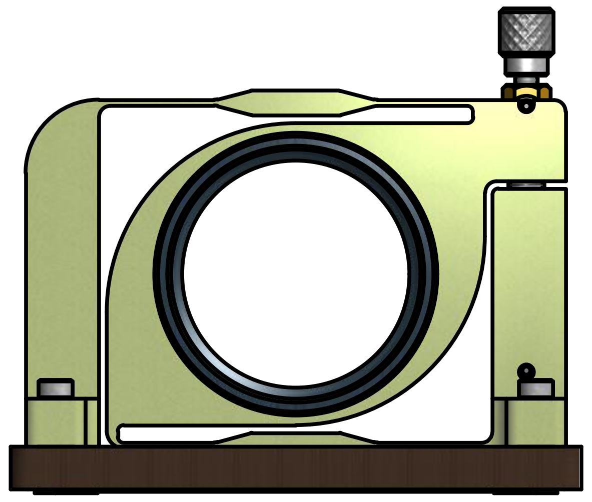

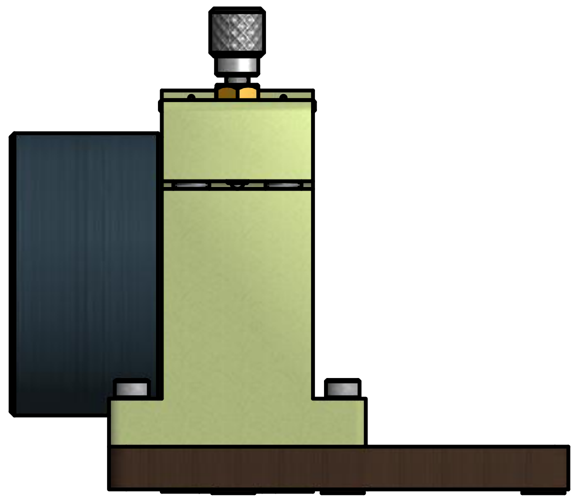

ITEM	QTY	FILE NAME	REV	PART NUMBER	VENDOR	DESCRIPTION	A.I. PART #	A.I. VENDOR
1	1	FLEXURE.ipt	-	J002534	HHMI: JFRC - ID&F	SINGLE AXIS FLEXURE	N/A	N/A
2	1	800 MICRON MOUNT - ILLUMINATION.ipt	A	J002536	HHMI: JFRC - ID&F	800 MICRON PI HERA STAGE ADAPTER FOR FLEXURE	N/A	N/A
3	1	NIKON ILLUMINATION OBJECTIVE ADAPTER.ipt	A	J002707	HHMI: JFRC - ID&F	FLEXURE ADAPTER FOR NIKON 10X ILLUMINATION OBJECTIVE	N/A	N/A
4	1	OLYMPUS ILLUMINATION OBJECTIVE ADAPTER - LONG.ipt	-	J003071	HHMI: JFRC - ID&F	FLEXURE ADAPTER FOR OLYMPUS 4X OBJECTIVE - LONG	N/A	N/A
5	4	91292A014.ipt	-	91292A014	MCMASTER	SHCS, M2.5X10mm	N/A	N/A
6	2	94135K1 - EXTENSION SPRING.iam	0	94135K1	MCMASTER	EXTENSION SPRING, 302 SS, 1.0" L X .312" OD X .035" WIRE	N/A	N/A
7	1	98380A422 - DOWEL PIN.ipt	-	9830A422	MCMASTER	DOWEL PIN, 416 SS, 1/16" X 3/4"	N/A	N/A
8	2	98380A416 - DOWEL PIN.ipt	-	98380A416	MCMASTER	DOWEL PIN, 416 SS, 1/16" X 5/16"	N/A	N/A
9	1	MAS15 - ADJUSTMENT SCREW.ipt	-	MAS15	THORLABS	ADJUSTMENT SCREW WITH KNOB, M3 X 0.25	N/A	N/A
10	1	N250L3 - ADJUSTMENT SCREW NUT.ipt	-	N250L3	THORLABS	FINE ADJUSTMENT NUT INSERT	N/A	N/A

PARTS LIST

NOTICE:
INFORMATION CONTAINED IN THIS DOCUMENT OR ANY REPRODUCTION THEREOF, IS PROPRIETARY INFORMATION AND PROPERTY OF HOWARD HUGHES MEDICAL INSTITUTE. IT SHALL NOT BE DISCLOSED, COPIED, DUPLICATED OR USED FOR MANUFACTURE, PRODUCTION OR PROCUREMENT, WITHOUT THE EXPRESS WRITTEN PERMISSION OF HOWARD HUGHES MEDICAL INSTITUTE.

(UNLESS SPECIFIED OTHERWISE)
PRIMARY UNITS: INCHES
[SECONDARY UNITS]: MILLIMETERS
PRIMARY TOLERANCES:

X.X	± 0.020
X.XX	± 0.010
X.XXX	± 0.005
X.XXXX	± 0.0005
ANGULAR	± 0.5 DEG

- DO NOT SCALE DRAWING -
THIRD ANGLE PROJECTION:

hhmi Howard Hughes Medical Institute
hhmi janelia Research Campus

FLEXURE HS-SIMVIEW ILLUMINATION.iam

SIZE	PART NUMBER	REV	SHEET
C	J002709	0	1 OF 1
