# Supplementary material for: Whole-central nervous system functional imaging in larval Drosophila
Source: Nat Commun. 2015 Aug 11;6:7924. doi: 10.1038/ncomms8924 (PMC4918770; doi:10.1038/ncomms8924)
Supplement: Supplementary Data 1 — Technical drawings of individual components and complete assemblies of custom four-axis stage and flexure systems in the hs-SiMView light-sheet microscope [file ncomms8924-s2.zip › Assemblies/Assembly - Spring Plunger (J002560).pdf]

REVISION HISTORY				
ZONE	REV	DESCRIPTION	DATE	APPROVED
	0	DO NOT FABRICATE		

GENERAL NOTES:
1. MATERIAL:
2. SPECIAL FINISH: **NONE**
3. SURFACE ROUGHNESS (UNLESS SPECIFIED OTHERWISE): **N/A** (AVERAGE MICRO-INCHES)
4. INTERPRET DIMENSIONS AND TOLERANCES PER ASME Y14.5M-1994
5. DEBURR AND BREAK ALL SHARP EDGES, MAX 0.010" (UNLESS SPECIFIED OTHERWISE)
6. PARTS ARE TO BE CLEAN AND FREE OF OIL, GREASE, AND OTHER CONTAMINANTS
7. DIMENSIONS INCLUDE ANODIZING, ELECTROPLATING, AND CHEMICALLY APPLIED FINISHES IF APPLICABLE

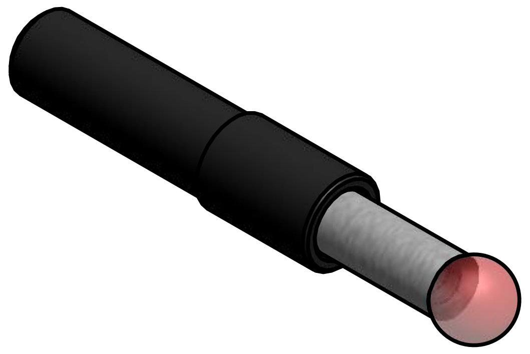

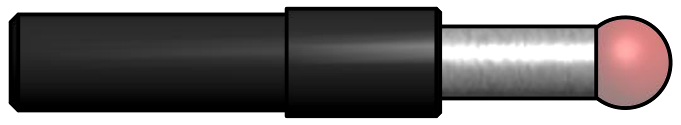

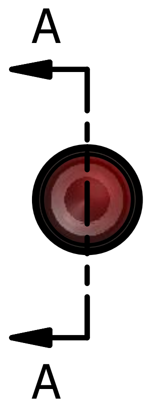

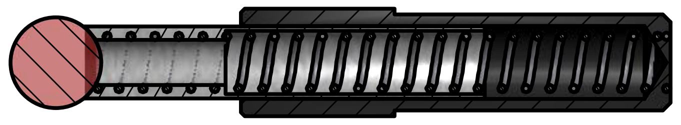

SECTION A-A

ITEM	QTY	FILE NAME	REV	PART NUMBER	VENDOR	DESCRIPTION	A.I. PART #	A.I. VENDOR
1	1	OO-50.ipt	-		CENTURY SPRING	COMPRESSION SPRING	N/A	N/A
2	1	SPRING PLUNGER TIP.ipt	-	43-830	EDMUND OPTICS	SPRING PLUNGER TIP	N/A	N/A
3	1	SPRING PLUNGER SLEEVE.ipt	-	J002561	HHMI: JFRC - ID&F	SPRING PLUNGER SLEEVE	N/A	N/A
4	1	SPRING PLUNGER SHAFT.ipt	-	J002562	HHMI: JFRC - ID&F	SPRING PLUNGER SHAFT	N/A	N/A

PARTS LIST

NOTICE:
INFORMATION CONTAINED IN THIS DOCUMENT OR ANY REPRODUCTION THEREOF, IS PROPRIETARY INFORMATION AND PROPERTY OF HOWARD HUGHES MEDICAL INSTITUTE. IT SHALL NOT BE DISCLOSED, COPIED, DUPLICATED OR USED FOR MANUFACTURE, PRODUCTION OR PROCUREMENT, WITHOUT THE EXPRESS WRITTEN PERMISSION OF HOWARD HUGHES MEDICAL INSTITUTE.

(UNLESS SPECIFIED OTHERWISE)
PRIMARY UNITS: INCHES
[SECONDARY UNITS]: MILLIMETERS

PRIMARY TOLERANCES:

X.X	± 0.020
X.XX	± 0.010
X.XXX	± 0.005
X.XXXX	± 0.0005
ANGULAR	± 0.5 DEG

- DO NOT SCALE DRAWING -

THIRD ANGLE PROJECTION:

hhmi Howard Hughes Medical Institute

hhmi janelia Research Campus

SPRING PLUNGER.iam

SIZE	PART NUMBER	REV	SHEET
C	J002560	-	1 OF 1
