# Supplementary material for: Whole-central nervous system functional imaging in larval Drosophila
Source: Nat Commun. 2015 Aug 11;6:7924. doi: 10.1038/ncomms8924 (PMC4918770; doi:10.1038/ncomms8924)
Supplement: Supplementary Data 1 — Technical drawings of individual components and complete assemblies of custom four-axis stage and flexure systems in the hs-SiMView light-sheet microscope [file ncomms8924-s2.zip › Components/J002557 - Focus Mic Clamp.pdf]

GENERAL NOTES:
1. MATERIAL: **ALUMINUM, 6061-T651**
2. SPECIAL FINISH: **ANODIZE PER MIL-A-8625F, TYPE II, CLASS 2 (BLACK)**
3. SURFACE ROUGHNESS (UNLESS SPECIFIED OTHERWISE): **63 Ra** (AVERAGE MICRO-INCHES)
4. INTERPRET DIMENSIONS AND TOLERANCES PER ASME Y14.5M-1994
5. DEBURR AND BREAK ALL SHARP EDGES, MAX 0.010" (UNLESS SPECIFIED OTHERWISE)
6. PARTS ARE TO BE CLEAN AND FREE OF OIL, GREASE, AND OTHER CONTAMINANTS
7. DIMENSIONS INCLUDE CHEMICALLY APPLIED FINISHES IF APPLICABLE

REVISION HISTORY

ZONE	REV	DESCRIPTION	DATE	APPROVED
	-	INITIAL RELEASE	6/23/2014	coopb

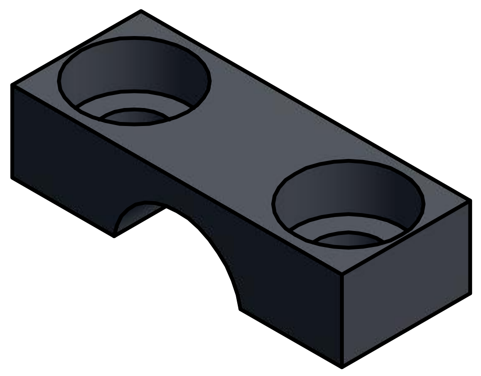

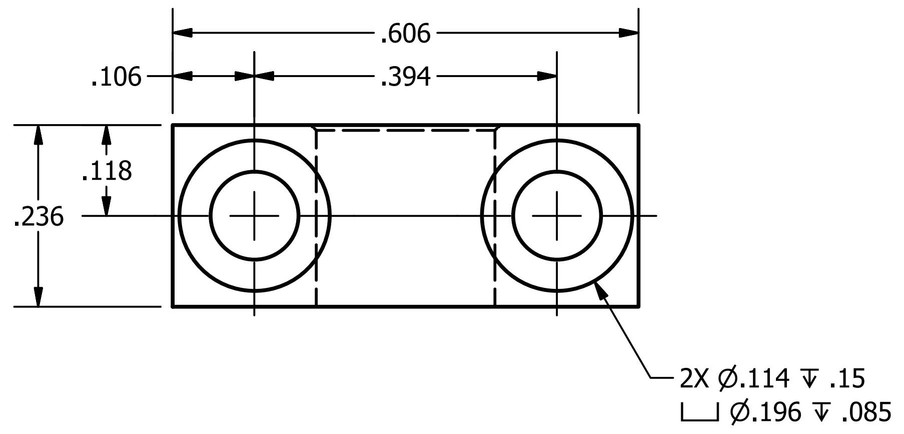

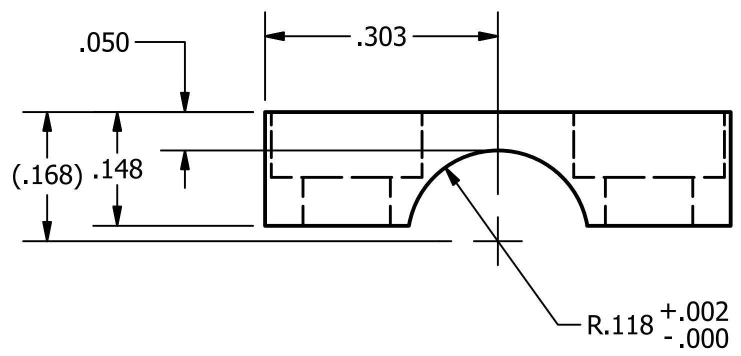

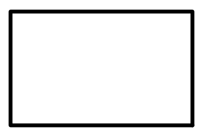

NOTICE:
INFORMATION CONTAINED IN THIS DOCUMENT OR ANY REPRODUCTION THEREOF, IS PROPRIETARY INFORMATION AND PROPERTY OF HOWARD HUGHES MEDICAL INSTITUTE. IT SHALL NOT BE DISCLOSED, COPIED, DUPLICATED OR USED FOR MANUFACTURE, PRODUCTION OR PROCUREMENT, WITHOUT THE EXPRESS WRITTEN PERMISSION OF HOWARD HUGHES MEDICAL INSTITUTE.

(UNLESS SPECIFIED OTHERWISE)
PRIMARY UNITS: INCHES
[SECONDARY UNITS]: MILLIMETERS

PRIMARY TOLERANCES:

X.X	± 0.020
X.XX	± 0.010
X.XXX	± 0.005
X.XXXX	± 0.0005
ANGULAR	± 0.5 DEG

- DO NOT SCALE DRAWING -
THIRD ANGLE PROJECTION:

HHMI
HOWARD HUGHES MEDICAL INSTITUTE

HHMI janelia farm research campus

FOCUS MIC CLAMP.ipt

SIZE	PART NUMBER	REV	SHEET
C	J002557	-	1 OF 1
