# Supplementary material for: Whole-central nervous system functional imaging in larval Drosophila
Source: Nat Commun. 2015 Aug 11;6:7924. doi: 10.1038/ncomms8924 (PMC4918770; doi:10.1038/ncomms8924)
Supplement: Supplementary Data 1 — Technical drawings of individual components and complete assemblies of custom four-axis stage and flexure systems in the hs-SiMView light-sheet microscope [file ncomms8924-s2.zip › Components/J002563 - Focus Wedge.pdf]

GENERAL NOTES:
1. MATERIAL: **STAINLESS STEEL, 440C, HRC 50-54**
2. SPECIAL FINISH: **NONE**
3. SURFACE ROUGHNESS (UNLESS SPECIFIED OTHERWISE): **32 Ra** (AVERAGE MICRO-INCHES)
4. INTERPRET DIMENSIONS AND TOLERANCES PER ASME Y14.5M-1994
5. DEBURR AND BREAK ALL SHARP EDGES, MAX 0.010" (UNLESS SPECIFIED OTHERWISE)
6. PARTS ARE TO BE CLEAN AND FREE OF OIL, GREASE, AND OTHER CONTAMINANTS
7. DIMENSIONS INCLUDE CHEMICALLY APPLIED FINISHES IF APPLICABLE

REVISION HISTORY

ZONE	REV	DESCRIPTION	DATE	APPROVED
	-	INITIAL RELEASE	6/23/2014	coopb
	A	UPDATED FOCUS WEDGE WITH RETURN SPRING AND REDUCED FRICTION	9/17/2014	coopb

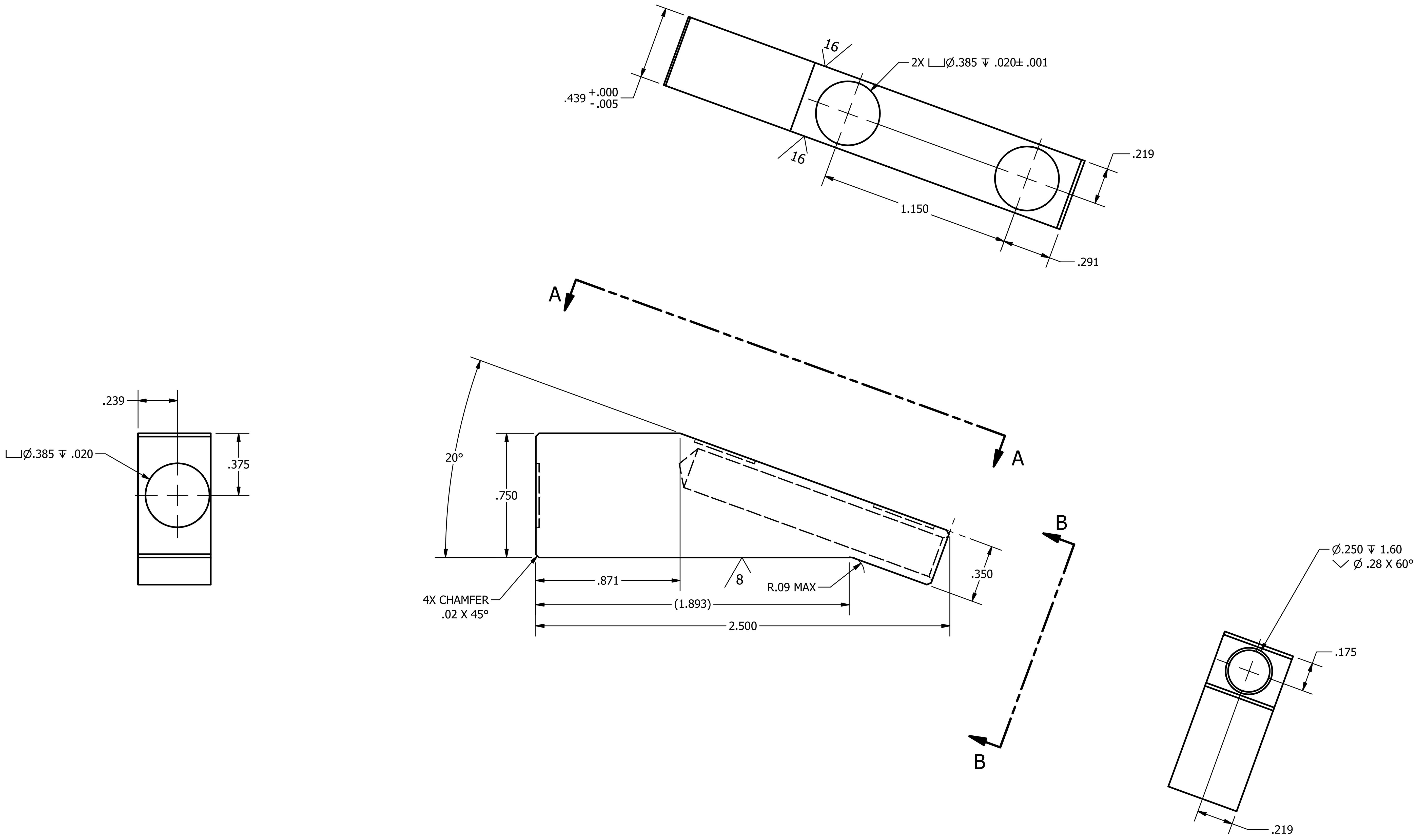

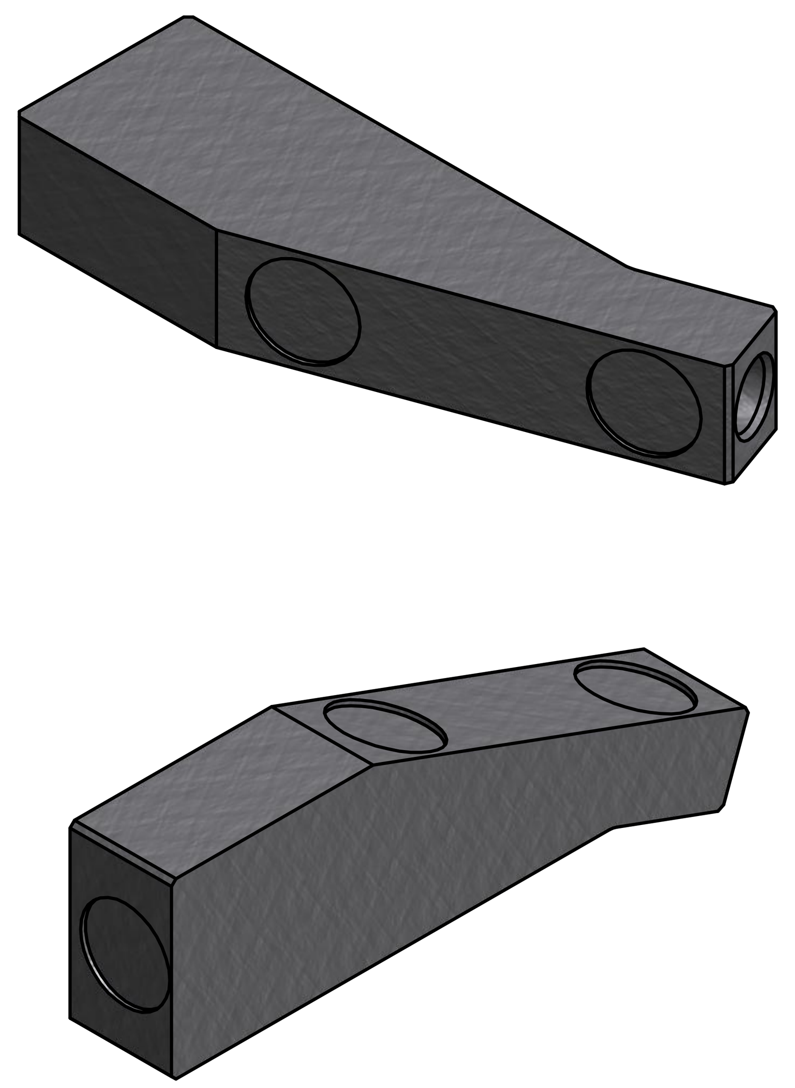

NOTICE: INFORMATION CONTAINED IN THIS DOCUMENT OR ANY REPRODUCTION THEREOF, IS PROPRIETARY INFORMATION AND PROPERTY OF HOWARD HUGHES MEDICAL INSTITUTE. IT SHALL NOT BE DISCLOSED, COPIED, DUPLICATED OR USED FOR MANUFACTURE, PRODUCTION OR PROCUREMENT, WITHOUT THE EXPRESS WRITTEN PERMISSION OF HOWARD HUGHES MEDICAL INSTITUTE.	(UNLESS SPECIFIED OTHERWISE) PRIMARY UNITS: INCHES [SECONDARY UNITS]: MILLIMETERS<br>PRIMARY TOLERANCES:<br>X.X ± 0.020<br>X.XX ± 0.010<br>X.XXX ± 0.005<br>X.XXXX ± 0.0005<br>ANGULAR ± 0.5 DEG<br>- DO NOT SCALE DRAWING -<br>THIRD ANGLE PROJECTION:	HHMI HOWARD HUGHES MEDICAL INSTITUTE — HHMI janelia farm research campus<br>FOCUS WEDGE.ipt<br>SIZE: D · PART NUMBER: J002563 · REV: A · SHEET: 1 OF 1
