# Supplementary material for: Whole-central nervous system functional imaging in larval Drosophila
Source: Nat Commun. 2015 Aug 11;6:7924. doi: 10.1038/ncomms8924 (PMC4918770; doi:10.1038/ncomms8924)
Supplement: Supplementary Data 1 — Technical drawings of individual components and complete assemblies of custom four-axis stage and flexure systems in the hs-SiMView light-sheet microscope [file ncomms8924-s2.zip › Readme.pdf]

Whole central nervous system functional imaging in larval *Drosophila*

William C. Lemon, Stefan R. Pulver, Burkhard Höckendorf, Katie McDole,
Kristin Branson, Jeremy Freeman and Philipp J. Keller

Howard Hughes Medical Institute, Janelia Research Campus

Supplementary Technical Drawings

Technical drawings of individual components and complete assemblies of custom four-axis stage and flexure systems in the hs-SiMView light-sheet microscope

Multi-view volumetric imaging using piezo positioning of the hs-SiMView microscope's detection objectives requires a finely adjustable (micrometer precision) and highly robust (multi-day operation of the imaging system) mechanical solution for relative position and orientation alignment of the detection piezos as well as for optimally matching piezo scan ranges to maximize the microscope's shared multi-view volumetric imaging range. We developed a custom two-part mechanical objective positioning system that serves (1) as an adjustable adapter between detection objectives and the piezo stages and (2) as an adjustable base for mounting piezo stages on the optical table (please see **Supplementary Fig. 2** in the Supplementary Materials). Technical drawings of these critical custom parts are provided in this archive.

Our custom positioning and rotation adjustment system provides micrometer precision for four key degrees of freedom: x-y-z-translation of the detection objective and rotation of the piezo base around the normal vector to the optical table. The custom flexures are manufactured from aluminum and designed to minimize mass and maximize stiffness resulting in minimal added load on the piezo stage (thus optimizing speed). The y-translation, z-translation and rotation degrees of freedom are incorporated in a highly dimensionally stable stainless steel base and facilitated by low-friction jewel-bearing guides. Precise movement is achieved through (1) two differentially adjustable sapphire tipped micrometers opposing a ruby tipped spring mechanism for y-translation and rotation adjustment and (2) a micrometer-positioned low-friction ceramic wedge for z-translation. The x-translation degree-of-freedom is incorporated in the flexure using

a precision adjustment screw opposing both gravity and a spring preload and a highly robust one-piece aluminum frame manufactured by wire electrical discharge machining (Wire EDM) designed to minimize tilt of the optical axis resulting from the objective's weight distribution. Together, these parts allow fine-tuning of the relative position and orientation of the microscope's four objectives with micrometer precision. The ability to perfectly match the limited scan range (250 micrometers) of fast piezo stages with high stiffness is furthermore crucial to maximize the sample volume that can be simultaneously imaged in both detection systems.

<u>Overview of technical drawings:</u>

- **Drawings of complete assemblies** (folder "Assemblies")
  We provide technical drawings of 3-axis piezo mount assemblies for 250 µm piezo stages ("Assembly – 3-axis Piezo Mount Small.pdf") and 800 µm piezo stages ("Assembly – 3-axis Piezo Mount Large.pdf"). The 250 µm high-speed piezo stages are used in the detection arms of the hs-SiMView microscope and are combined with the flexure system shown in "Assembly – Flexure for hs-SiMView Detection Arm.pdf". The 800 µm long-range piezo stages are used in the illumination arms of the hs-SiMView microscope and are combined with the flexure system shown in "Assembly – Flexure for hs-SiMView Illumination Arm.pdf". A technical drawing of the spring plunger assembly used in both types of piezo mounts is provided in "Assembly – Spring Plunger (J002560).pdf" (part number J002560).

- **Drawings of individual components** (folder "Components")
  Names of technical drawings for individual components used in the piezo mount and flexure system assemblies described above start with the part number "J00XXXX", followed by the part name. In total 21 custom-built parts are required to create the piezo mount and flexure assemblies. Technical drawings of each of these components are included in this archive.
