# Supplementary material for: Whole-central nervous system functional imaging in larval Drosophila
Source: Nat Commun. 2015 Aug 11;6:7924. doi: 10.1038/ncomms8924 (PMC4918770; doi:10.1038/ncomms8924)
Supplement: Supplementary Data 2 — Source code for processing multi-view functional imaging data, detecting and classifying fictive locomotor behaviors, and mapping activity timing across the central nervous system [file ncomms8924-s3.zip › Readme.pdf]

Whole central nervous system functional imaging in larval *Drosophila*

William C. Lemon, Stefan R. Pulver, Burkhard Höckendorf, Katie McDole,
Kristin Branson, Jeremy Freeman and Philipp J. Keller

Howard Hughes Medical Institute, Janelia Research Campus

Supplementary Software

Source code for processing multi-view functional imaging data, detecting and classifying fictive locomotor behaviors, and mapping activity timing across the central nervous system

This archive contains our custom software tools for (1) data management of large-scale hs-SiMView image data, including modules for background masking and lossless image compression, (2) multi-view processing of hs-SiMView image data, including modules for multi-view image registration and multi-view image fusion, (3) ΔF/F processing of large-scale functional imaging data, including modules for specimen drift compensation, sliding-window baseline computation and ΔF/F calculation, (4) automatically detecting and classifying fictive locomotor behaviors from functional imaging data and (5) high-resolution mapping of activity timing across the central nervous system. The software has been developed for and tested on functional imaging data produced by high-speed piezo-based simultaneous multi-view (hs-SiMView) light-sheet microscopy.

All algorithms were developed and tested in Matlab (version R2013b, The MathWorks). In addition to the Matlab core installation, the Curve Fitting Toolbox, Image Processing Toolbox, Statistics Toolbox, Optimization Toolbox and Signal Processing Toolbox are required to execute the code. Multi-threaded execution through the included job management scripts furthermore requires the Parallel Computing Toolbox. Software compatibility was verified for PCs with Windows 7 or Windows 8 64-bit operating systems.

All software modules follow a highly memory-efficient design that enables efficient application of the computational framework to multi-terabyte light-sheet microscopy data sets using a single computer workstation. For maximum performance, we recommend a workstation with at least 12

CPU cores clocked at 2 GHz, 64 GB of memory and a fast RAID system for data storage. However, the entire framework can also be effectively used on lower-end systems. Memory requirements scale linearly with the number of requested parallel threads (which is an adjustable parameter in our software modules), and we have successfully used the pipeline on desktop computers equipped with a quad-core CPU and 32 GB of memory.

<u>Overview of software modules:</u>

Part I: <u>Lossless image compression for post-acquisition data management</u>

- **clusterPT.m** (folder "Part 1 – Data Management")

  Description: This program is a job management script for the function **processTimepoint.m**, which removes image artifacts produced by sCMOS cameras and greatly reduces image data size. Core capabilities to this end include sCMOS dead-pixel correction, background masking and lossless image compression.

  Input data structure: Raw input data can be provided either as 3D images in binary format or as single 2D images in TIF format.

  a) When using 3D images in binary format, data should be provided following this naming scheme and directory structure:

  […]/SPC00_TM**ttttt**_ANG000_CM**x**_CHN00_PH0.stack

  In this format, '**ttttt**' corresponds to the time point (5 digits) and '**x**' to the camera index (1 digit).

  b) When using 2D images in TIF format, data should be provided following this naming scheme and directory structure:

  […]/SPM00/TM**ttttt**/ANG000/SPC00_TM**ttttt**_ANG000_CM**x**_CHN00_PH0_PLN**pppp**.tif

  In this format, '**ttttt**' corresponds to the time point (5 digits), '**x**' to the camera index (1 digit), and '**pppp**' to the plane index in the 3D image stack (4 digits).

Irrespective of the choice of the input data format, the program furthermore expects one dark image per camera (i.e. single background images acquired with closed shutter) to be present, using the following naming scheme:

For data format (a): […]/Background_**x**.tif

For data format (b): […]/SPM00/Background_**x**.tif

As above, '**x**' corresponds to the camera index (1 digit).

Finally, **clusterPT.m/processTimepoint.m** has been designed to read experiment meta data, specifically the three-dimensional dimensions of the image stacks, from XML files provided together with the TIF image data.

For data format (a), the program will expect to find one global XML file in the following location:

[…]/ch0.xml

For data format (b), the program will expect to find one XML file per time point in the following locations:

[…]/SPM00/TM**ttttt**/ch0.xml

The code will look for the following type of entry in the XML file:

<info dimensions="**X1**x**Y1**x**Z1**,**X2**x**Y2**x**Z2**" />

Here, '**X1**' and '**X2**' are the horizontal image sizes for cameras 1 and 2 (in pixels, using as many digits as needed), '**Y1**' and '**Y2**' are the vertical image sizes (in pixels) and '**Z1**' and '**Z2**' are the number of image planes in the image stacks.

If XML files are not available for a given imaging experiment or the user wishes to not use this meta data feature, one can instead simply provide empty files at the locations listed above (however, files must be present for the code to execute correctly) and instead provide information about image dimensions directly via the configuration parameter `dimensions` in **clusterPT.m**.

Important configuration parameters: `segmentFlag` (enable/disable background masking), `thresholds` (adaptive threshold(s) used if background masking is enabled).

Related files: **processTimepoint.m**

Relationships with other pipeline modules: **clusterPT.m** generates input data required by **clusterMF.m**.

Part II: Multi-view image registration and fusion

- **clusterMF.m** (folder "Part 2 – Multi-View Image Fusion")

  Description: This program is a job management script for the function **multiFuse.m**, which performs multi-view image registration and fusion for individual time points of an imaging experiment. This program marks the first phase for multi-view image processing of time-lapse experiments. In the next step, **multiFuse.m** is followed by **localAP.m** and **clusterTF.m** to produce the final, temporally smooth multi-view fusion results across the entire time-lapse experiment.

  Important configuration parameters: `maskFactor` (adaptive threshold used if background masking was disabled in **clusterPT.m**), `fusionType` (type of image fusion applied after image registration; options include adaptive blending [default], geometrical blending, wavelet fusion and averaging), `leftFlags`/`flipHFlag`/`flipVFlag`/`frontFlag` (parameters indicating distribution of high-contrast image content in the multi-view data set, based on the relative orientation of cameras, light sheets and specimen), `xOffsets`/ `yOffsets` (for dual-camera acquisition, defines search range for lateral offsets of specimen positions in the image data recorded with the two cameras).

  Related files: **multiFuse.m**

  Auxiliary functions from MathWorks File Exchange: **fInterpolate.mexw64**

  Relationships with other pipeline modules: **clusterMF.m** reads data format generated by **clusterPT.m** and generates input data required by **localAP.m** and **clusterTF.m**.

- **localAP.m** (folder "Part 2 – Multi-View Image Fusion")

  Description: This program is a job management script for the function **analyzeParameters.m**, which is a data crawler for smoothing and interpolating parameters obtained with **clusterMF.m** for the purpose of multi-view image registration and multi-channel/camera intensity matching. **localAP.m** generates look-up-tables with temporally filtered transformation parameters for use by **clusterTF.m**. **localAP.m** thus represents the bridge between initial multi-view fusion of a coarsely-sampled set of time points with **clusterMF.m** and subsequent high-throughput, temporally smooth multi-view fusion of an entire time-lapse experiment with **clusterTF.m**.

  Important configuration parameters: `smoothing` (the second value of this 1×2 vector provides the temporal window size used for smoothing the registration parameters obtained with **clusterMF.m**).

  Related files: **analyzeParameters.m**

  Relationships with other pipeline modules: **localAP.m** reads data format generated by **clusterMF.m** and generates input data required by **clusterTF.m**.

- **clusterTF.m** (folder "Part 2 – Multi-View Image Fusion")

  Description: This program is a job management script for the function **timeFuse.m**, which performs multi-view image registration and fusion for entire time-lapse imaging experiments, based on registration look-up-tables and fusion masks generated by **localAP.m** and **clusterMF.m**, respectively. This program marks the second phase for multi-view image processing of time-lapse experiments.

  Important configuration parameters: `fusionType` (type of image fusion applied after image registration; options include adaptive blending [default], geometrical blending, wavelet fusion and averaging), `leftFlags`/`flipHFlag`/`flipVFlag`/`frontFlag` (parameters defining distribution of high-contrast image content in the multi-view data set, based on the relative orientation of cameras, light sheets and specimen).

  Related files: **timeFuse.m**

  Auxiliary functions from MathWorks File Exchange: **fInterpolate.mexw64**

Relationships with other pipeline modules: **clusterTF.m** reads data format generated by **clusterPT.m**, **clusterMF.m** and **localAP.m**.

Part III: Specimen drift compensation, sliding-window baseline and ΔF/F computation

- **clusterRS.m** (folder "Part 3 – Functional Data Processing")

  Description: This program is a job management script for the function **registerStacks.m**, which compensates for specimen drift in the three-dimensional image data. Drift correction is performed via plane-by-plane image translation. **registerStacks.m** also prepares a median-filtered version of the image data for a subset of time points, which is used for sliding-window baseline calculation with **localCR.m**.

  Important configuration parameters: `references` (selection of time points combined into an average image stack that serves as a reference for global image registration), `dffSampling` (temporal spacing for computation of ΔF/F baseline stacks, provided in unit of time points), `kernelSize` (kernel size of median filter used for computation of ΔF/F baseline stacks).

  Related files: **registerStacks.m**

  Relationships with other pipeline modules: **clusterRS.m** reads data format generated by **clusterPT.m**, **clusterMF.m** and **clusterTF.m** and generates input data required by **localCR.m**.

- **localCR.m** (folder "Part 3 – Functional Data Processing")

  Description: This program is an image processing script that generates a sliding-window baseline estimate for subsequent computation of ΔF/F data with **clusterCD.m**.

  Important configuration parameters: `dffSampling` (temporal spacing for computation of ΔF/F baseline stacks, provided in unit of time points; this parameter should be set to the same value used in **clusterRS.m**), `dffRadius` (radius of time window used for sliding-window baseline calculation, provided in units of `dffSampling`; for example, if `dffSampling` is set to T_1 and `dffRadius` is set to T_2, the sliding-window size for baseline calculation is

$[-T_1 \times T_2, +T_1 \times T_2]$, using a spacing of T_2 time points within this window), `percentile` (percentile used for baseline calculation).

Related files: none

Relationships with other pipeline modules: **localCR.m** reads pre-processed image data generated by **clusterRS.m** and generates input data required by **clusterCD.m**.

- **clusterCD.m** (folder "Part 3 – Functional Data Processing")

  Description: This program is a job management script for the function **calculateDelta.m**, which performs ΔF/F computation for the spatially registered time-lapse image data set.

  Important configuration parameters: `dffSampling` (temporal spacing for computation of ΔF/F baseline stacks, provided in unit of time points; this parameter should be set to the same value used in **clusterRS.m** and **localCR.m**), `subOffset` (1×2 vector that indicates whether input image data need to be background-corrected; if the first value is set to 1, the second value is subtracted from all image stacks), `meanFraction` (fraction of mean reference stack intensity that is added to the denominator in the ΔF/F calculation to limit noise in low-signal regions), `scaling` (1×2 vector providing information about scaling of ΔF/F output data, which allows optimal use of the 16-bit dynamic range of the output data format; the first and second values provide offset and multiplier, respectively, used to adjust the dynamic range of ΔF/F output data), `forceZero` (1×2 vector providing information used for noise suppression at the sample/agarose interface; if the first value is set to 1, this optional feature removes voxels with signal levels below the second value).

  Related files: **calculateDelta.m**

  Relationships with other pipeline modules: **clusterCD.m** reads data format generated by **clusterRS.m** and **localCR.m**.

Part IV: <u>Detection and classification of fictive locomotor behaviors</u>

- **detectWaves.m** (folder "Part 4 – Locomotor Wave Detection")

  Description: This program is a data analysis script for detecting and classifying locomotor waves. In order to use **detectWaves.m**, specify a base data location as `basepath`, and an

individual file by the relative path to its location (`datestamp`) and its file prefix (`datestamp_file`). Each data set must contain at least two files, *name-TIME-SERIES.mat* and *name-ROI-MAP.mat*, where *name-TIME-SERIES.mat* contains a (*n* ROIs × *n* time points) matrix `ts` and *name-ROI-MAP.mat* contains a vector `rois` with linear indices for each signal, a vector `segment` that indicates which segment each signal is from (1 through 8) and a vector `LR` that indicates laterality (0 or 1). With these variables set, the script will generate a scrollable visualization. Please see Section "*Wave Detection from Region of Interest Time Series*" in the **Methods** accompanying the main paper for details on the principles and theory underlying this wave detector.

Important configuration parameters: Please see lines 48-54 in **detectWaves.m** for the main parameters of the script and their brief description.

Related files: **drawDots.m**

Part V: <u>Three-dimensional mapping of activity timing at the single-voxel level</u>

- **computeInformationGain.m** (folder "Part 5 – Activity Timing Mapping")

  Description: This program is an image processing and data analysis script for mapping activity timing across the nervous system at the single-voxel level. Input image data is provided as a set of Matlab files in .MAT format (following the naming scheme dffData**X**.mat, where **X** is an integer number), each containing a four-dimensional matrix named `dffData` that represents an image data window temporally centered on an event of interest (such as a locomotor wave). The first three dimensions of `dffData` are reserved for the spatial dimensions of the image data and the fourth dimension represents time. The activity timing map is then computed using the combined information provided by all data windows. Please see Section "*Mapping of whole-CNS activity timing*" in the **Methods** accompanying the main paper for details on the principles and theory underlying this activity timing mapping.

  Important configuration parameters: `ignoreval` (intensity level in the input image data that indicates missing information, such as for background regions masked off by **clusterPT.m**; voxels with missing information are ignored in the mapping of activity

timing), `mint1` and `maxt1` (time window, defined as [`mint1 maxt1`], used to define dynamic range when setting colors in the output timing maps), `filterwidths_try` ($1 \times n$ vector defining temporal widths of block function, provided in unit of time points, used to compute fit given time).

Related files: **learnIntervalClassifier.m**, **createSubPlots.m**, **myParse.m**, **saveTIF.m**

Appendix A: Auxiliary tools

- **localCP.m** (folder "Part 2 – Multi-View Image Fusion")

  Description: This program is a job management script for the function **collectProjections.m**, which is a data crawler for collecting maximum-intensity projections generated by **clusterMF.m** and **clusterTF.m**. All projections are subsequently combined into image stacks for easy visualization and inspection of time-lapse image data.

  Important configuration parameters: `configurations` (defines data sets analyzed by the data crawler and enables data collection for multiple types of data sets with a single call of the script).

  Related files: **collectProjections.m**

  Relationships with other pipeline modules: **localCP.m** reads data format generated by **clusterMF.m** and **clusterTF.m**.

- **convertData.m** (folder "Part 1 – Data Management")

  Description: This program is a data crawler for converting image data between TIF and KLB file formats. The data crawler searches and converts image data in the entire directory sub-tree relative to the root folder in which the script call occurred.

  Important configuration parameters: `processingMode` (flag for switching between TIF-to-KLB compression and KLB-to-TIF decompression).

  Related files: none

  Relationships with other pipeline modules: **convertData.m** reads/writes TIF and KLB files generated by any module of the processing pipeline (or external software).

Appendix B: Auxiliary functions for reading and writing TIF and KLB image formats

- **readImage.m** (folders "Part 1 – Data Management", "Part 2 - Multi-View Image Fusion", "Part 3 – Functional Data Processing")

  Description: This function is used throughout the processing pipeline for reading various types of image formats.

  Related files: **readKLBstack.mexw64**, **msvcp120.dll**, **msvcr120.dll**

  Auxiliary functions from MathWorks File Exchange: **readTIFstack.m**

  Notes: Additional support for reading KLB headers is provided by the MEX file **readKLBheader.mexw64**.

- **writeImage.m** (folders "Part 1 – Data Management", "Part 2 - Multi-View Image Fusion", "Part 3 – Functional Data Processing")

  Description: This function is used throughout the processing pipeline for writing various types of image formats.

  Related files: **writeKLBstack.mexw64**, **msvcp120.dll**, **msvcr120.dll**

  Auxiliary functions from MathWorks File Exchange: **writeTIFstack.m**
